# Documenting and defining emergent phenomenology: theoretical foundations for an extensive research strategy

**DOI:** 10.3389/fpsyg.2024.1340335

**Published:** 2024-07-10

**Authors:** Olivier Sandilands, Daniel M. Ingram

**Affiliations:** ^1^Emergence Benefactors, Huntsville, AL, United States; ^2^Emergent Phenomenology Research Consortium, New Market, AL, United States

**Keywords:** meditation, psychedelics, emergence, phenomenology, experience, medicine, ethics, emergent phenomena

## Abstract

Meditation, psychedelics, and other similar practices or induction methods that can modulate conscious experience, are becoming increasingly popular in clinical and non-clinical settings. The phenomenology associated with such practices or modalities is vast. Many similar effects and experiences are also reported to occur spontaneously. We argue that this experiential range is still not fully described or understood in the contemporary literature, and that there is an ethical mandate to research it more extensively, starting with comprehensive documentation and definition. We review 50 recent clinical or scientific publications to assess the range of phenomena, experiences, effects, after-effects, and impacts associated with a broad variety of psychoactive compounds, meditative practices, and other modalities or events. This results in a large inventory synthesizing the reports of over 30,000 individual subjects. We then critically discuss various terms and concepts that have been used in recent literature to designate all or parts of the range this inventory covers. We make the case that specialized terminologies are needed to ground the nascent research field that is forming around this experiential domain. As a step in this direction, we propose the notion of “emergence” and some of its derivatives, such as “emergent phenomenology,” as possibly foundational candidates.

## 1 Introduction

### 1.1 Background

Cross-cultural anthropologists tell us that there are “upwards of 4,000 known societies, approximately 90% of which have one or more institutionalized forms of Altered-State of Consciousness induction” (Bourguignon, 1973; Locke and Kelly, 1985). This represents a substantial portion of the global population and of cultures. Yet, these “altered states” (ASCs henceforth) are not well-understood despite their centrality to many cultures, religions, and philosophical systems. They are often pathologized in both traditional and mainstream clinical contexts. Although there are issues with the notion of ASCs, which will be discussed below, this salient fact will serve as a platform on which to ground the present discussion.

On the more challenging and thus clinically-relevant end of the experiential spectrum, some extreme presentations can resemble what, according to current mainstream clinical practice, are mental disorders or episodes. The overlap, equivalence, and separation of these is still unclear.

This is why, in 1993, the Diagnostic and Statistical Manual (DSM) added criteria to exempt those from a diagnosis of mental illness who were experiencing hallucinations, delusions, or other unusual experiences that were expected in their spiritual tradition who also maintained sufficient function and did not meet other criteria for mental illness.^1^

Examining the text of the most recent edition of the DSM (DSM-5-TR, American Psychiatric Association, 2022) itself, we find there is simultaneously a profound upgrade from what came in the DSM-IV and before, but also a significant lack in the degree of depth and detail that the added material presupposes should be available to clinicians either in their clinical education, life experience, or in further supplementary references.

The DSM-5-TR provides few characterizations of the religious, spiritual or culturally-sanctioned experiences which call for modifier labels and may be collectively referred to here as “spiritual exemption criteria.” These are limited and hard to apply, reflecting a lack of awareness of the norms of the various religious or spiritual traditions, but also a lack of research into the experiences themselves, and/or a lack of integration of existing findings into mainstream contexts.

What to define as “hallucinations” and “delusions” is clearly culturally determined, as histories of the topic reveal (e.g., Spittles, 2022). Hence such definitions and the risks, benefits, and alternatives to their adoption require careful consideration. Moreover, the Z65.8 diagnostic code “Religious or spiritual problem,” is framed largely in terms of questions of faith and culture rather than other sorts of experiences.^2^

Other guidebooks on religious or spiritual experiences for healthcare providers do not, to our knowledge, offer a comprehensive presentation of the full range of phenomena and experiences of interest here, nor of the practices or triggers which can lead to these manifestations. Instead, they also mostly focus on other aspects of spiritual or religious life that may be relevant in a therapeutic setting. For instance, the World Psychiatry Association’s Position Statement on Spirituality and Religion in Psychiatry (Moreira-Almeida et al., 2016) proposes that religion and spirituality should play a more important role in psychiatric practice, but its guidelines are quite general, although the second proposition states: “An understanding of religion and spirituality and their relationship to the diagnosis, etiology and treatment of psychiatric disorders should be considered as essential components of both psychiatric training and continuing professional development.”

Similarly, the World Health Organization, Quality of Life Spirituality, Religion and Personal Beliefs Group (2006) has recognized the importance of religion and spirituality on quality of life but does not provide much documentation on the phenomenology nor theory of these experiences. This is also true of major medical textbooks such as Tintinalli et al. (2020) or Rosen’s/Walls et al. (2022) (for emergency medicine). Other works about spiritual competency in clinical contexts tend to propose very general skillsets and often do not much discuss the details of the experiences in question nor ways to manage difficult variants nor cultivate desirable ones — see, e.g., Vieten and Scammell (2015).

In sum, although major international resources like the latest International Classification of Diseases (ICD-11, World Health Organization, 2019) and the DSM-5-TR contain diagnostic/billing codes for “Spiritual or Religious Problems,” there is, to our knowledge, no definitive resource on the experiences and phenomena which the various religions, spiritual traditions, and cultural contexts consider as normal, expected, desirable, or pathological. Yet, without such a resource, how could healthcare workers be expected to adequately respond to the challenging variants of these phenomena, and correctly differentiate them from pathology?

A large and growing body of anecdotal evidence clearly shows that these experiences are *not* limited to specific cultural, religious, or spiritual circumstances or institutions, but are reported to occur:

(1) Within cultural, institutional or practice contexts that do not have concepts or frameworks to recognize or explain them (Lindahl et al., 2017, 2020, 2022a,b). Crucially, the lack of a normative framework, or conflict between unexpected or novel experiences and one’s worldviews or expectations, are often associated with increased difficulty processing or integrating them (Bitbol, 2014; Lindahl et al., 2022a,b);

(2) Outside of any established tradition, either as:

- Intentionally cultivated through various individual and group-level modalities and practices conducive to such experiences, perceptions, or personal changes. The variety of such activities is immense, including but not limited to specific types of internal gestures as in prayer, hypnosis, meditation and contemplation (Grabovac, 2014; Schlosser et al., 2019; Sparby and Sacchet, 2022; Galante et al., 2023), psychoactive substances (Breeksema and van Elk, 2021; Timmermann et al., 2022), movement (Piarulli et al., 2018), breathing exercises, rhythmic music (TaKeTiNa, drumming), chanting, sensory deprivation, electrical stimulation (Piarulli et al., 2018), and more. Many of these are becoming increasingly popular in clinical settings [e.g., mindfulness-based intervention or psychedelic-assisted therapy (Mitchell et al., 2023)] and in secularized and cross-cultural religious or spiritual contexts, or
- As unintended side effects of engaging in such practices (Lindahl et al., 2017). As quoted in Galante et al. (2023): “Increasing evidence shows a wide range of experiences that can arise with regular mindfulness meditation, from profoundly positive to challenging and potentially harmful”;

(3) Unintentionally or seemingly “spontaneously” — emerging from a convergence of causes that we do not currently understand — with various sociocultural, personal, and contextual factors probably playing an important role (Locke and Kelly, 1985; Miller and C'de Baca, 2001; Lindahl et al., 2022b; Timmermann et al., 2022; Yaden and Newberg, 2022). A broad range of such triggers is reported in various literatures, e.g., nature, stress, childbirth, grief, trauma, loss, diet, sleep deprivation, sexual relations, aesthetic and artistic experiences, and more (Sartre, 1957; Locke and Kelly, 1985; Lapassade, 1987; Fink, 1995; James, 2005; Hulin, 2008; Sandilands, 2019; Corneille and Luke, 2021; Yaden and Newberg, 2022).

On the other end of the spectrum, modern research and many religions or spiritual traditions claim remarkable benefits can be achieved through various cultivation methods (James, 2005), beyond what is usually envisioned in most recent research, which often focuses on relaxation, efficiency, stress-reduction, alleviating depression, coping with pain, etc. Although it is important to distinguish between ideals and achievable human goals, this also warrants further investigation.

### 1.2 An ethical mandate

The high prevalence in most times and places of the experiences in question demonstrates their enduring importance for humans. This stands in stark contrast with the paucity of information on these which is available to the public in many mainstream contexts, including contemporary healthcare systems.

Researchers have been calling for a dramatic increase in the *scope* of phenomena that should be carefully considered, as well as in the systematicity, multidisciplinarity, quality, variety, and long-term coordination of research endeavors (Sparby, 2017; Van Dam et al., 2018; Vieten et al., 2018; Galante et al., 2023).

From a medical ethics perspective, as we will detail in a forthcoming article, it is clear there is a mandate to:

- Fill that knowledge gap so as to avoid harming patients presenting with challenging emergent phenomena because of ignorance (thereby respecting the ethical principle of **Nonmaleficence**);
- Promote beneficial effects by exploring the remarkable claims of positive outcomes made by countless secular, religious, or spiritual traditions which cultivate the positive side of these experiences (**Beneficence**);
- Provide information on risks, benefits, and alternatives in clinical but also in all non-medical contexts that promote activities which may lead to the experiences in question (**Autonomy**);
- And abide by the principle of **Justice**^3^ by distributing benefits and burdens equitably.

It follows that thoroughgoing research into this domain, as well as strategies to integrate important findings in the various contexts we outlined, should be developed. In this context, we advocate for the principles of: (1) *ontological neutrality* — suspending ontological judgments and instead focusing on phenomenology and clinical pragmatism —, (2) *neutrality* and *global scalability* regarding terminological and conceptual choices, (3) *high epistemological* and *methodological rigor* for fundamental qualitative and empirical research, and (4) the pragmatic goal of improving human flourishing and reducing suffering.

### 1.3 Aims and method

Given the complexity of the causal processes likely involved, we believe much work is still needed to elucidate these types of experiences and phenomena. We also believe that before solid empirical evidence can be obtained, much descriptive, definitional, conceptual, and review work are required. Indeed, all the current or past research projects we are aware of suffer from various limitations on these fronts.^4^

To help those dealing with the particular categories of experiences and effects which are delineated at length, in an open-ended but still very specific and detailed way, in section 2 below, the creation of a new board-certified clinical specialty may be required.

To do so, one must first define a body of knowledge that the specialty covers which has not been adequately covered by the scopes of previous boards and has demonstrable clinical relevance. The present article aims to formally begin that process, and proposes a functional, clinical, pragmatic approach that may lead to the definition of a specialty, and at the very least will help advance our understanding of this domain.

Such a program should be founded on what is often regarded as the fundamental stepping stone of any scientific endeavor — description and definition (Hoyningen-Huene, 2013; Sparby, 2017). This in turn requires extensive documentation, the development of dedicated terminologies, and contemporary data-driven taxonomies.

The present contribution starts with description, inventorying a selection of 50 recent scientific publications which document phenomena, experiences, after-effects, and impacts in the short to long term associated with various modalities of intentional cultivation, including various psychedelic compounds, meditative and contemplative practices, and therapies explicitly based on such meditative/contemplative techniques or psychedelics, for example clinical applications of mindfulness, as well as similar experiences and effects that occurred outside of those contexts and sometimes without an obvious cause.

We then critically discuss various concepts and terms used in recent research to reference the general domain delineated in the first part of this article.

Finally, based on the observation that, as has been the case for most new fields of knowledge in modern history, professional lexicons with specialized terminologies need to be developed, we introduce the notions of “emergence,” “emergent phenomena,” and related concepts, and discuss their merits, limitations, and relation with other common words such as ASCs, religion, spirituality, and mysticism.

The present article is meant to pave the way to long-term, coordinated, high-quality experimental research, but is not an empirical paper. Rather, it proposes a novel synthesis of existing qualitative data, followed by extensive analytical discussion meant to provide clarification in this notably elusive domain. Its core proposals are descriptive and definitional.

Note that our goal is not to impose our own preferred views, methods, and/or terms, but rather to help expand and move the field forward by integrating clinical, scientific, and spiritual paradigms. We hope that fruitful debates will follow and lead to much needed normalization.

## 2 Documenting the range of phenomena, experiences, effects, and after-effects of interest

### 2.1 Methodology

To help define our domain of interest and document its broad scope, we decided to survey recent scientific publications from our field. To that end, we selected 38 clinical, psychiatric, or qualitative publications on meditation, 37 on psychedelics, and 21 for other induction methods or “spontaneous” experiences, striving to include a diversity of modalities, study-designs, and thematic foci to make sure our sample would provide an accurate picture of this experiential landscape. Because the extant literature has various limitations and is too large to comprehensively review, we have opted for an open-ended approach, manually selecting higher-quality than average publications, rather than conducting a keyword search on selected databases, e.g., following the Preferred Reporting Items for Systematic reviews and Meta-Analyses (PRISMA) guidelines (Page et al., 2021). We did not formally assess the quality of each paper for similar reasons.

Indeed, as discussed at length in Supplementary file S2, we acknowledge that most of these studies have limitations in terms of rigor or precision, including:

- A lack phenomenological and/or meditative expertise of subjects and/or researchers;
- The use of sometimes crude tools, particularly in the case of clinical trials with low-quality scales for outcome measures;
- Uncertain causal attribution;
- Biased interpretations or appraisals;
- Evaluations or appraisals which do not take into account the sometimes non-linear development of some of these phenomena (Fisher, 2019), etc.

We thus excluded publications which (1) did not contain new qualitative information, for instance articles that mostly focused on physiological correlates or on philosophical considerations, by reviewing the methods sections and excluding articles which did not employ first-person methods, (2) only used pre-existing structured questionnaires, since these provide information about prevalence and correlation of specific factors, but no additional phenomenology, or (3) were too specific [e.g., a micro phenomenological study of the experience of mind-wandering during meditation (Petitmengin et al., 2017)].This elicited a corpus of 23, 22, and 15 publications for each of the three groups, respectively.

We then reviewed, extracted and recategorized the qualitative reports of phenomena, experiences, and after-effects and impacts associated with these various modalities in the immediate, short-, mid- and long-terms into a large and synchronic thematic framework that covers a wide variety of functional and experiential dimensions.

Our data-analysis was based on the micro-phenomenological method (Petitmengin, 2006; Petitmengin et al., 2017, 2019). New labels were added to the categorizations found within the publications when the qualitative data seemed to require it. The analytical scheme was frontloaded by synthesizing several theoretical frameworks of a descriptive or functional nature borrowed from classical phenomenology, recent qualitative publications, and biomedical research [e.g., the Research Domain Criteria (Cuthbert and Insel, 2013)], as detailed in Supplementary file S1.

Given our focus, we excluded all non-phenomenological information that could not be generalized to the whole sample, such as demographics, context, intensity, severity, duration, appraisal, prevalence.

Once we approached a form of categorial saturation, meaning analyzing new articles did not seem to add new themes to our inventory, we stopped, ending up with a total of 50 articles. The titles and types of these publications are listed in Supplementary file S2, in a table which also contains information on modality, substance, or precipitating context, and number of participants.

### 2.2 Results

Our main result is a fine-meshed functional and descriptive inventory of experiences, effects, after-effects, and impacts associated with various induction modalities and contexts, representing the aggregated experiences of 30,100 individual subjects, thus covering a vaster phenomenological range than any research initiative we are aware of.

Since the data we synthesized is substantial and would far exceed length requirements, what follows is a short table listing the types of emergent experiences and effects we identified, along with their subdimensions, illustrated by a single sample item. Nota bene: in cases where a given category has several subdimensions, we only include 1 or 2 sample items for the general category, rather than giving a sample for each sub- or sub-subcategory (Table 1).

**Table 1:** Variety of emergent phenomena, experiences, and effects with sample item.

Category (see Supplementary Table S1)	Subdimensions	Sub-subdimensions	Sample item
Aesthetic or numinous			Finding beauty in a difficult experience (Sublimation)
Archetypal, visionary, mythical, or symbolic			Sense of encountering “seemingly autonomous entities,” “nonphysical entities,” “physical beings,” or “presences”
Arousal-related			Difficulty sleeping
Behavioral			Uncontrollable laughter
Cognitive	Attention	Disposition	Effortless attention; Confusion.
		Dynamics	
		Ease	
		Focus	
		Saturation	
		Shape	
	Clarity		
	Intensity		
	Memory		
	Meta-cognition/ meta-awareness		
	Thinking	Activity	
		Clarity	
		Control	
		Ease	
		Speed	
		Structure/organization	
Collective			Sense of a “collective energy”
Contextual*			N/A
Dimensional			Perception of alternate or higher dimensions
Emotional			Amazement
Energetic			Sensing energetic sensations, forces, or power in the body
Existential	Sense of self	Agency and free-will	Sense of connection, identification, or union of the self with aspects or all of one’s environment; Sense that actions are doing themselves
		Dualities	
		Extension	
		Identification	
		Location	
		Ownership	
		Perception/representation of self	
		Self-world boundaries	
	Sense of reality		
Expressive			Difficulty describing the experience
Functional			Episodes of powerlessness
Hedonic valence			Trouble enjoying things
Informational			Sense of receiving non-verbal communication
Intuitive			Wordless understanding
Magical			Sense of experiencing increased synchronicities
Medical			Hospitalization
Meta-emergent			Sense that an experience was “surreal”
Motivational			Increased motivation to practice
Motor	Balance		Involuntary movements
	Bodily control		
	Coordination		
	Movements/Gestures		
	Reflexes		
Paradigmatic	Cosmology (Origins of the world)		Positive philosophical or spiritual insights
	Epistemology		
	General Beliefs or Views		
	Ontology	Selfviews	
		Worldviews	
Perceptual	Capacities		Seeing short to long-lasting, simple to highly complex and multidimensional images
	Content	Hearing	
		Mind	
		Multimodal	
		Proprioception	
		Taste and smell	
		Vision	
	Structure	“Envelope” or temporal shape of perceptions	
		Shape	
		Speed	
Physiological	Cardiovascular		Physical Pain
	Gastrointestinal		
	Muscles and bones		
	Nervous system		
	Sensory systems		
	Skin		
	Thorax and lungs	Breathing	
		Chest	
	Urinary System		
Psychological	Affiliation		De-repression
	Biographical memories		
	Difficulties		
	Ideation		
	Insights		
	Personal narratives/ Psychological Ego		
Semantic			Disintegration of conceptual meaning structures
Sensate	Capacities		Increased color vividness
	Content	Hearing	
		Interoception	
		Mind	
		Proprioception/Kinesthesis	
		Smell/tase	
		Touch	
		Vestibular sensations	
		Visual	
	Structure	Synesthesia	
		Transmodal sensations	
Sexual			Increased libido
Social	Perception/Representation of Self		Increased sense of being positively regarded by others during social interactions
	Perception/Representation of others		
	Sociability		
Spatial			Sense of being outside space
Temporal			Slowing of time perception
Vocational			Changes in career path
Volitional			Sense of being possessed, abducted, or invaded
Wakeful			Intentionally-induced momentary extinction of experience

*This category, identified through the process described in Supplementary Table S1, was not instanciated in our literature review, but is included nonetheless as hypothesized to be useful in future iterations of this inventory.

The full inventory can be found in Supplementary file S2. It contains a full list of reviewed publications with relevant accompanying information, commentary on the quality of reviewed research, discussion of the phenomenological and/or meditative expertise of researchers and subjects, appraisals, etiology, and further breakdown of our methodological choices.

We strongly advise that the reader consult this file, as it is much larger than the table included in the present section, and because, despite some degree of overlap between the categories it is divided into, this extensive list will help define the domain we are interested in, thus grounding the terminological and conceptual considerations which follow.

## 3 Discussion

### 3.1 Defining our domain

Although it should be seen as the first iteration of an open and expansive collection which will be refined and upgraded, the Supplementary file S2 inventory conveys in both broad and very specific terms what we mean by this field we are working to help define, although the latter is not limited to the contents of this inventory.

It must be noted that the phenomena and effects we are interested in, including but not limited to those found in Supplementary file S2, are not modality specific, as there is considerable overlap across induction-methods. What is outlined here seems to be a specific domain of experience and perhaps development that cannot be adequately broken-up on the sole basis of practice-type or context of occurrence. This hypothesis is supported by existing literature, although the latter is currently somewhat scattered throughout traditions and disciplines.

Overall, and as alluded to in our introduction, what many call Altered-States of Consciousness (ASCs) comes closest to the domain we are trying to define here. The notion does convey the intuitive unity of the experiential and practical domain of concern, and recent initiatives such as the Altered-States Database (Schmidt and Berkemeyer, 2018; Prugger et al., 2022) align with our core hypothesis — namely, that the dedicated study of this domain across induction methods and contexts of occurrence is possible and desirable.

However, major issues with the static and vague term of ASCs, detailed in section 3.5.1 below, lead us to the firm conclusion that another name is required.

Crucially, we feel that approaches which rely exclusively on existing psychometric scales do not adequately cover the range of experiences and effects of interest, as these instruments often suffer from limited synchronic and diachronic precision (i.e., macro and micro-phenomenological details of the character and/or temporal unfolding of the “states” being studied), often use ambiguous or vague terms and wordings, and rely on pre-interpretations, theories, and sometimes arbitrary or logically inconsistent statements, to the point that it is sometimes virtually impossible to know what experience a person is actually reporting when responding through a Likert grade. We unpack these specific issues in section 3.5 below.

As discussed in the introductory remarks of inventory Supplementary file S2, these pitfalls are specifically what first-person methods like micro-phenomenology (which we used in our re-analysis) are designed to avoid.

Even though standard statistical tools may find good psychometric properties for such scales, we believe this does not fully compensate for the issues caused by a problematically vague initial description of the phenomena in question — which is one of the core reasons why we believe that better and more extensive description and improved definition of terms are needed.

We believe these various limitations in prevalent concepts and scales, a lack of precision which can usually be extended to the description of induction methods, risk hindering outcomes research (Lundh, 2022; Sparby and Sacchet, 2022). This may have negative downstream implications on the reliability, validity, and replicability of the research that will be informed by it — possibly at the eventual detriment of practitioners and patients.

One of the ambitions of the present inventory is the development of structured questionnaires that are more precise, extensive, and less ambiguous than existing instruments, to then launch large-scale empirical studies employing an array of methods, such as micro-phenomenological interviews, and structured questionnaires based on further iterations of the present inventory.

Now that we have defined our domain by providing extensive, though preliminary documentation, we turn to definition, and critically discuss some of the most common terms used in recent research to designate our domain.

### 3.2 Discussing existing terms

It must be said that many secular and religions or spiritual traditions possess their own vernaculars for experiences and effects that seem to fall within our domain. A few examples are:

1. *Conversion*, *Dark night of the soul*, *Unio mystica*,^5^ *Contemplation*, *Theoria*,^6^ *Theosis*,^7^ *Nepsis*,^8^ *Glorification*^9^ [Christianity — see, e.g., James (2005), Depraz (2008), St. John of the Cross (2010), and Rose (2016)]
2. *Hal*,^10^ *Maqām*,^11^ *Qabd*,^12^ *Fanā’*,^13^ *Wajd*,^14^ *Junun*^15^ [Islam — see Fisher (2022)]
3. *Samādhi*,^16^ *Chakras*,^17^ *Moksha*^18^ [Hinduism — see, e.g., Rose (2016)]
4. *Jhānas*,^19^ *Siddhis*,^20^ *Nirodha*, *Nirodha Samāpatti*^21^ [Buddhism – see, e.g., Sayadaw (1994), Rose (2016), Sparby (2019), and Laukkonen et al. (2023)]
5. *Hitbodedut*,^22^ *Tardama*,^23^ *Halom*, *Hazon*, *Ayin*,^24^ *Bittul*^25^ [Judaism — see, e.g., Fisher (2022)].
6. *Metanoia*,^26^ *Henosis*^27^ [Ancient Greece and Neoplatonism — see Mazur et al. (2021) and Hadot (2002)]

Such notions often have specific canonical characteristics, usually described as *gestalts*,^28^ which could be seen as combinations of the more granular categories in Supplementary file S2 and analyzed in the same way. For instance, in canonical Buddhist texts, states of *jhāna* are often described through a five- or sixfold dimensional matrix of interrelated and covariant volitional, cognitive-attentional, affective and sensate factors.^29^ Different states of *jhāna* [8 or 9 are distinguished — see, e.g., Laukkonen et al. (2023)] have different “signatures” of relative salience of these various components. This is quite similar to the approach advocated for by Vion-Dury and Mougin (2016) — i.e. to characterize various “states” as specific *modalizations*, specific combinations of basic phenomenal dimensions.

However, we will not attempt such an analysis in the present paper, though it will be the subject of a later article. Importantly, traditional systems usually fit these into developmental and etiological theories. These should also be reviewed, although we cannot do this here.

Our focus will rather be the wide variety of analogous terms and typologies which have been used in recent scientific research. Among these there are larger “umbrella” terms, and a plethora of specific experience-types and variants. These are usually differentiated based on their content, structure, context, or the modality or hypothetical cause that induced them.

Broader, collective terms include *spiritual experiences* (e.g., Yaden and Newberg, 2022); *religious experiences* (James, 2005); *spiritual emergence/emergency* (Grof and Grof, 1989; Louchakova, 2006); *spiritually transformative experiences* (Kason, 2019; Brook, 2021); and *altered-states of consciousness* or ASCs (Tart, 1969; Lapassade, 1987), among others. We will discuss these in section 3.5.

For now we will focus on narrower, individual types and names including: *contemplative* and *meditative* experiences (Lindahl et al., 2017); *psychedelic* experiences; *mystical* experiences (Stace, 1987; James, 2005; Streib et al., 2020); *mystical experience with psychotic features* (Lukoff et al., 1995); *transpersonal* experiences (Grof and Grof, 1989); *archetypal* experiences (Jung, 1980; Hillman, 2004); *peak*-, *nadir*- and *plateau*-experiences (Maslow, 2014); *self-actualization* (Maslow, 1943); *spontaneous spiritual awakenings* and *spontaneous kundalini awakening* (Corneille and Luke, 2021); *energy-like somatic experiences* (Cooper et al., 2021); *kundalini* experiences (Woollacott et al., 2021) or *kundalini syndrome* (Benning et al., 2019); *extraordinary* or *anomalous* experiences (Vieten et al., 2018); *non-ordinary states of consciousness* (Aixalà, 2022); *psychic* phenomena and *extrasensory perceptions* (Aixalà, 2022); *unusual abilities* (Locke and Kelly, 1985); *near-death experiences* (Greyson and Stevenson, 1980; Jung, 1989; Charland-Verville et al., 2014; Martial et al., 2019); *out-of-body experiences* (Bünning and Blanke, 2005; Mudgal et al., 2021); *transcendent* states or *transcendental* experiences (Wahbeh et al., 2018); *self-transcendent* states (Hanley et al., 2020); *quantum change* (Miller and C'de Baca, 2001). These often branch out into further sub- or sub-subtypes which we will not go into to remain in scope.

From our perspective, which looks at the experiences and effects documented in the inventory (Supplementary file S2) as a single experiential group, and from a pragmatic, phenomenological, ontologically agnostic, outcomes focused, therapeutic perspective, these terms share various problems, as they are:

(1) *Excessively specific* — many do not convey the general perspective we are adopting. This is not to say that there aren’t common, intuitive *types*^30^of phenomena such as *mystical* or *archetypal*, as we do use such labels in our inventory. Rather, that our own interests here include many labels since we look at the full range of effects, even those which would probably not be seen as *mystical* or *archetypal* by most. Regarding, e.g., “spiritually *transformative* experiences,” and “*self-*actualization”: clearly, not all the experiences we are concerned with here are necessarily reported to be “transformative” or “actualizing.”
(2) *Metaphysically bound* and/or *Interpretative* rather than descriptive. “Kundalini,” “extrasensory perceptions,” “psychic phenomena,” “transpersonal,” “transcendent,” “transcendental,” etc., all imply various degrees of speculation about the ontological nature of an experience, its etiology, or its epistemology. As a single example, *transpersonal* experiences are so named because they are interpreted as the surfacing of memories belonging to a hypothetical psychological dimension which Jung called the “collective unconscious.” The notion thus implies a combination of metaphysical and etiological assumptions which could be debated.
(3) *Modality-bound*, when in practice, there is considerable overlap across modalities in terms of phenomenology.
(4) *Culturally-bound* and/or *Idiosyncratic,* e.g., “nadir experience,” “Kundalini,” “awakening,” “Spontaneous spiritual awakening,” “quantum change.” These words possess specific histories, coming from specific traditions (e.g., “awakening” likely comes from translations of Buddhism), where they often possess a range of complex, highly technical, and sometimes contradictory definitions and criteria that vary by denomination and individual teachers and interpreters.
(5) *Syncretic*, e.g., “Kundalini Syndrome,” “quantum change.”
(6) *Not representative* of the widespread prevalence of such experiences and effects in most cultures in most times and place, as implied by adjectives like “unusual” or “extraordinary.”
(7) *Pathologizing*, or otherwise excessively normative, e.g., “anomalous” or “altered.”
(8) *Imprecise*, descriptively, linguistically, historically, or philosophically, e.g.:

(a) “*Transcendental*” has several meanings:

(i) It may carry a connotation of grandiosity or majesty (such as the *Transcendental Études* by romantic composer and virtuoso Franz Liszt);
(ii) It may relate to specific meditation techniques (such as *Transcendental meditation*);
(iii) It may be used technically: Kantian transcendental philosophy is not about specifically “transcendental” experiences or states (an oxymoron), but about the conditions of possibilities and limits of experience (“transcendental aesthetics”) and knowledge (“transcendental logic”). As quoted in the *Critique of Pure Reason* (Kant, 2007): “I call all knowledge transcendental if it is occupied, not with objects, but with the way that we can possibly know objects even before we experience them.” This has limited overlap with what we are talking about here, and instead was a very influential philosophical innovation, partly a reaction to British empiricism, which shaped the history of post-Kantian thinking, and directly influenced the birth of classical phenomenology — which Husserl called a form of “transcendental philosophy” (Husserl, 1970; Fink, 1995) —, and in recent developments in the philosophy of science (De la Tremblaye and Bitbol, 2022);
(iv) There is such a thing as transcendental mind states in Husserl’s philosophy, and these are linked with accomplishing the *épochè* and the “transcendental reduction” (Bitbol, 2019; Blouin, 2022). But the meaning of these terms is controversial (Barber, 2021; Stone and Zahavi, 2021), and it is doubtful that, even if we managed to agree on the fact that the post-*épochè* transcendental reduction leads to one or several specific “states” of mind, that these would resemble those induced by, for example, Transcendental Meditation, which are quite varied and would hardly all fit under the label “transcendental experience” (Castillo, 1990);
(v) Furthermore, the term transcendental is often confused with the word “transcendent” but is quite different.

(b) *Expressions* using this latter term, such as *Transcendence/Transcendent states/Self-transcendent experiences*, also have many possible meanings, implications, and uses:

(i) While the term sometimes carries “esoteric” connotations, logically speaking, a “transcendent experience” makes little sense, since what is transcendent is in fact “beyond experience,” transcending what we presently experience (which is *immanent*): how could we experience what is beyond experience? Yet this is precisely the implication of the notion of a “transcendent state.” This point is made by some Abrahamic mystical theologians, when they talk about the unknowability of a transcendent or hidden god, *deus in absconditus,* as in church father pseudo-Dionysius’ *Mystical Theology* (Pseudo-Dionysius, 1987).
(ii) A more subtle critique is that “transcendence” is not only characteristic of specific experience types, since it is in some ways inherent to our “natural attitude” (in the Husserlian sense) when relating with the objects and subjects which populate our world. This is a fundamental point made by classical or theoretical phenomenologists (Husserl, 1970; Henry, 2008) and various other philosophical schools (e.g., Madhyamaka). Philosophically speaking, whenever one adopts a realistic worldview that posits the existence of a stable entity *in* or *behind* ordinary mundane phenomena, there is transcendence — because we usually neither reduce “objects” nor other “subjects” to the present incomplete perception we have of them. As quoted in De La Tremblaye and Bitbol (2022): “Any intentional aiming of an object … intends infinitely more than what is intuitively given. (The object) is a Kantian teleological idea (Pradelle, 2008). This ‘infinitely more’ which the expectations of a present consciousness imply, is precisely what we mean when we claim that an object ‘transcends’ consciousness.” Thus, how could this adequately differentiate specific experiences, when it is seen to also characterize most natural worldly perceptions and interactions, barring hypothetical non-intentional or non-dual consciousness (Henry, 2008; Husserl, 2019)?
(iii) It must be mentioned that Maslow used this term to qualify the highest level of self-development in his hierarchy of needs, following “self-actualization” needs (Maslow, 1943). In this context “self-transcendence” seems to refer to an overcoming of previous limitations, including outgrowing narrow egoic identifications, or to various forms of experiences of expansion perceived as being “beyond” other modes of experience. This is understandable, yet following our analyses, the term still appears as too specific, historically charged, and ambiguous to be a good candidate.

Thus, we have not found any existing term that is entirely appropriate *for our goals*. These are of course fine terms on their own and in their own contexts. However, we see the need for a dedicated terminology as has occurred with the development of most fields of knowledge (e.g., medicine or mathematics) to facilitate universal communication.

### 3.3 Emergent phenomena, experiences and effects

We hypothesize that if our understanding of the domain we have delineated is to be developed and scale globally, a key step is to develop dedicated terms that avoid the issues we outlined above that might prevent global adoption. The flip side of this thesis is our assertion that adoption of problematic terminology was and is a major factor in the failure of every previous attempt to integrate knowledge of the experiences in question into the global clinical and mental health mainstreams.

We also hypothesize that determining optimal clinically and scientifically operationalizable definitions and taxonomies of these phenomena, effects, and transformations that scale globally is a challenge which will require extensive basic and applied research, as well as global linguistic and conceptual negotiations and compromises.

As a first step in this direction, we propose to refer to these effects and experiences (as listed in the inventory), as “emergent phenomena, experiences and effects” (EPEEs henceforth), or sometimes “emergent experiences,” or “emergent phenomena,” which, depending on how they are related to, may be felt to be “emergent crises,” “emergent benefits,” or simply “emergence.”

This choice came from a long-term, collective process of trying to find the most scientifically descriptive, non-ontological, accurate, scalable term for this broad category of experiences, processes, and effects. We did not invent the term “emergence,” as it has been used in other disciplines (e.g., complexity science) for a long time. But we did notice a parallel with the way these disciplines use this term, noting that these experiences seem to “emerge” from an extremely complex system in ways that currently cannot be predicted by their currently known constituent parts or other underlying principles. We also noted a synchrony with Transpersonal Psychology and its notion of “spiritual emergence” but realized that it might not always be clear why certain effects might necessarily be described as “spiritual,” as discussed in section 3.2, hence our choice. We would like to thank and acknowledge Dr. Kathryn Devaney, who initially proposed the term, and the other early members of the consortium of researchers who coalesced around this shared concern to form Emergent Phenomenology Research Consortium^31^ for their valuable insights and innovations in initial and essential linguistic discussion. This terminology has started being used in recent academic literature (Salguero, 2023; Wright et al., 2023).

Here a *phenomenon* refers to a specific subjective sensation, perception, or object, a *gestalt* (see the definition above) which does not include the entirety of the field of experience or of a given duration of time. An *experience* is more “complete” — it includes the totality of the lived world of the subject for a given duration. Also, the term *phenomenon* is often used to point to a more “objective” event than the term *experience*, which tends to point to the *subjective* pole. An *effect*, in this context, can refer to a phenomenon or experience, when these are apprehended through the lens of their hypothetical *cause*, of which they are an effect. It is also used when studying the *impacts*, on various experiential or functional domains, of a given *phenomenon* or *experience* seen as the cause of these impacts, which can also be referred to as *after-effects*.

#### 3.3.1 Emergent practices or modalities

We will refer to the intentional cultivation modalities associated with EPEEs as “emergent practices,” though this term realistically must encompass the circumstances that make their arising more likely, even if they were not intentional practices and occurred spontaneously. Intentionality is not necessary for a modality to increase the likelihood of emergent phenomena. Examples would include someone practicing meditation in a secular materialist tradition who was unaware of and uninterested in emergent phenomena and effects, or someone taking a psychedelic recreationally.

#### 3.3.2 Process of emergence

Contrary to the synchronic phenomenology contained in the inventory, what we will call the “Process of emergence” includes a diachronic, developmental, or progressive element, however potentially non-linear, often relating to a series of emergent phenomena which have marked the subject, or perhaps produced some sort of transformation, regardless of the valuation of that transformation. Various developmental systems have been proposed for this sort of process over the millennia, as well as for the possible predicted sequences in which these might lead to a process of emergence.

We will now review potential arguments in favor of these linguistic choices.

### 3.4 Advantages and disadvantages of these terms

#### 3.4.1 Advantages

From a clinical lens, we hypothesize that these terms are *scalable*, in the sense that they:

- Are simple, clear, and sufficiently free of cultural baggage to be used within clinical and scientific contexts,
- Are not proprietary, nor from a particular orthodoxy,
- Carry fewer connotations than many words such as “spiritual,” “religious,” or “mystical” (see below for a detailed discussion).

From a philosophical perspective, they are closer to an ideal of ontological neutrality, meaning they are relatively devoid of metaphysical content, assumptions, or implications. Obviously, there is a limit to “neutrality,” and some argue “methodological agnosticism” is in fact impossible or a hindrance (Blum, 2018). However, the reality of much of clinical experience is very aligned with the notion of ontological or methodological agnosticism, and this seems to be functional in practice. Good clinicians, and good emergent teachers, often avoid this issue in working with people to meet them where they are, establish therapeutic relationships, work with their assumptions and cultural conditioning, to promote good outcomes.

These terms avoid cultural appropriation or bias, and syncretism. From an anthropological lens, it is a neutral etic^32^ framework that allows the study of various religious or spiritual traditions with specific, emic cultural frameworks, while maintaining their integrity. Syncretism — the often imprecise and rough fitting amalgamation of various traditions, religious and spiritual frameworks and philosophies into a whole, is off-putting and often offensive to both orthodox traditions and beyond, as demonstrated by over 140 years of syncretic solutions failing to gain any significant traction in the clinical and scientific mainstream, thus posing a massive barrier to the ethical scaling of the research and study of this field.

We explicitly aim to avoid directly contradicting the core assumptions of major scientific and orthodox traditions, and instead adopt a clinically pragmatic stance which focuses on what frameworks add value to care, improve outcomes and human flourishing.

Other potential advantages are that the terms “emergence” and derivates add historical and epistemic connectedness, as they are auspiciously coherent with previous usages of similar terms to refer to the same general process, e.g., with the notion of *spiritual emergence* (Grof and Grof, 1989).

Semantically, they preserve and convey something of the upward movement which has often traditionally been associated with what we call the process of emergence, with the vocabulary of elevation being present in many spiritual tropes of the various religious or spiritual traditions, e.g., the ascent of mount Sinai (Judaism–Christianity); the ascent of mount Carmel or *Epéktasis* as “ascent toward God” in John Climacus (Christianity); *anabasis*/*catabasis* as the elevation/descent of the spirit in the Greek Mystery Schools; the *ascent* of Muhammad toward god in Quran, LIII, 4–10 (Islam); the contemplative process of *rising up* the jhānas, with the rarest and most refined of these modalizations being referred to as the *higher* jhānas (Buddhism); the movement toward “highest values” which defines spirituality according to Pargament et al. (2013); Plotinian “mystical ascent” (Mazur et al., 2021, chap. 4); and so on. We do not mean to imply that every “emergent process” necessarily leads “upward” to something better or higher, just to point the linguistic resonance with numerous traditional characterizations of the ultimate trajectory of these processes.

Etymologically, *ex-* means “out” and *mergō* “to dip, to immerse, to plunge.” This conveys something of the possible beneficial nature of these experiences and of the associated emergence process, which are often traditionally associated with soteriologies^33^ and interpreted as a process of purification of coming out of some undesirable moral or experiential predicament or state (sin, ignorance, dukkha, etc.) and even something of the notion of “conversion” or being “twice-born” (James, 2005).

Metaphorically, they reflect the possibly sudden mode of onset of EPEEs and discontinuous tipping points in emergent processes described in some traditions [e.g., Britton et al. (2014) argue that the course of meditative progress suggests a nonlinear multiphasic trajectory] — emerging from water happens gradually but there are discontinuities such as the precise moment when one begins to surface, or the moment when one is completely out of the water.

#### 3.4.2 Disadvantages

Clearly, there is overlap with uses in other disciplines which may create confusion. The terms “emergence” and “emergent phenomena” (or perhaps more commonly, “emergent properties”) are indeed used in various disciplines such as in math, biology, cognitive science, economy, philosophy, and more. But this is also the case with, e.g., the word “organic,” which can qualify organic chemistry (all chemistry involving carbon), organic food (food that is grown without chemical fertilizers), organic art (spontaneous artistic expression), organic processes (processes of development that occur spontaneously/naturally in living beings), etc.

While there is indeed a semantic commonality, these are quite different meanings. Yet, few people would report any issues with this polysemy, as it is easy to differentiate between these various meanings based on context. Perhaps those who still feel the conflict with complexity science could simply use terms such as “emergent experience” or “emergent phenomenology.”

Another issue is that new words like we propose may not be seen favorably by those who have adopted other terminology in their work or practice over the years (or centuries). As we said, the solution here might be to consider these new terms (and further terminologies which may be proposed by others) as defining an *etic* framework, the use of which could remain limited within the community who work in the specialized field of research we believe is being defined here, and let *emic* frameworks be untouched by this and freely reject or adopt these terms. These questions are open for debate.

### 3.5 Clarifying thematic contours

As we alluded to earlier in this article, there are four central notions we wish to discuss in more detail to clarify their relationship with the way we conceptualize EPEEs. These are: (1) Altered-states of consciousness (ASCs), (2) Spirituality/Spiritual experiences, (3) Mysticism/Mystical Experiences and (4) Religion/Religious experiences.

#### 3.5.1 Emergence and altered states of consciousness

As we alluded to in the introduction and when discussing the Altered-State Database, the notion of altered states of consciousness can be limiting and problematic when approaching EPEEs in the way we wish to. It may apply broadly to *some* of them, but when looked at in more detail, terminological issues with each word that composes the expression “altered” “states” of “consciousness” appear.

Indeed, the term *state* implies a rigidity or stability which is not a good description of the rapidly changing nature of experience (Le Blanc-Louvry, 2015; Vion-Dury and Mougin, 2016). Some more seemingly stable “states” sometimes arise, and that is of course reflected in the common usage of this term, but that is not always the case. It may sometimes cover up the often rich detail of the temporal shape of some or all of these “stable states,” including, e.g., the Buddhist jhānas. As a case in point, recent micro-phenomenological investigations into these “states” demonstrate that, in practice, they are developmentally and experientially richer and more complex than a formulaic description conveys (Sparby, 2019).

The notion of “*alteration*” also implies a baseline stable state that is then departed from or altered, but it is impossible to precisely identify such a reified, baseline state, since our levels of vigilance, attention, attitudes, paradigms, etc. constantly modulate the contents of our experience. In short, the phenomenal features of what Husserl called the “living present” is a “field of actuality” (De La Tremblaye and Bitbol, 2022) and changes continuously. This also poses important issues at a philosophical level (Vion-Dury and Mougin, 2016). Again, this may work in everyday language, but not in the context of a dedicated, systematic, precise technical lexicon.

Many traditions consider what some might consider as “*altered*” states to, in fact, represent natural and even expected manifestations of the process of emergence (Grabovac, 2014). This includes the development of, e.g., attentional expertise. Hence, the adjective seems inadequate in this context too, as is not in keeping with this specific developmental perspective and set of expectations, in the same way that it would be strange to call an adult an “altered baby” or adolescence an “altered state of consciousness” (with no disrespect implied to parents who do come to this functionally descriptive conclusion). Furthermore, not all emergent phenomena are associated with an emergent process: to illustrate this point, we can mention how not all psychedelic experiences, which might be powerfully “altered,” will necessarily trigger what we might consider a process of emergence. Not all emergent development is caused by intense or exotic experiences, either (Yaden and Newberg, 2022).

Conversely, some emergent traditions consider that the hypothetical “baseline state” that would be *altered* to become ASCs is in fact itself the “altered” and deluded one: for instance, the essence of mind schools of Tibetan Buddhism (Mahāmudrā and Dzogchen) consider the “ordinary mind” (*sems*) as a distortion or misinterpretation of “awake awareness” (*rigpa*) or the “natural state” (Bkra-shis-rnam-rgyal and Tweed, 2001; Bdud-ʼjoms-glin˙-pa and Wallace, 2016).

Furthermore, the notion is clearly not practically sufficient to refer to many experiences and effects reviewed in the inventory (Supplementary file S2), including but not limited to:

- Energy-like somatic experiences (e.g., sensations of a “vibration cascading through the body,” or “extremely painful energy flows” as reported in Cooper et al., 2021);
- Shifts in metaphysical beliefs, which can be triggered by meditation (Lindahl et al., 2017) and/or psychedelics (Timmermann et al., 2022), and influence outcomes (Lindahl et al., 2022a);
- Motor effects such as involuntary movements [Cooper et al. (2021) report “jumpiness,” “whole-body jerking,” “spontaneous […] back-bending,” and “shaking”], which are commonly reported spontaneous side-effects of meditation practice;
- Changes to basic wakefulness levels (Britton et al., 2014);
- Functional effects such as increased or decreased executive or cognitive function (Brook, 2021);
- Profound vocational and life-trajectory impacts (Nicholson, 2014);
- Rarer instances such as voluntarily induced “cessations of consciousness” which are clearly neither phenomena, experiences, nor states, since they are by definition devoid of any experiential content, but rather “events,” which can only be characterized somewhat indirectly (Berkovich-Ohana, 2017; Winter et al., 2020; Chowdhury et al., 2023; Laukkonen et al., 2023).

Evidently, such reports are not states *per se*, but they are not really “*of consciousness*” either, since they cover domains as varied as physiology, sensations, kinesthesis, behavior, function, baseline vigilance levels, etc. We could extend the definition of “consciousness” to include all these aspects into a pervasive “field of consciousness,” as do Vion-Dury and Mougin (2016), but then, what else could there be a state of? This would render the adjective redundant.

#### 3.5.2 Emergence, religion, and spirituality

As quoted by Lukoff et al. (1998), the *Thesaurus of Psychological Index Terms* (Walker, 1991) states that religiosity “is associated with religious organizations and religious personnel” (p. 184) whereas spirituality refers to the “degree of involvement or state of awareness or devotion to a higher being or life philosophy. Not always related to conventional religious beliefs” (p. 208).

In the more recent American Psychological Association handbook of psychology, religion and spirituality, Pargament et al. (2013) provide similar definitions:

*“We define spirituality as ‘the search for the sacred.’ […] The term sacred is used inclusively here to refer not only to concepts of God and higher powers but also to other aspects of life that are perceived to be manifestations of the divine or imbued with divine-like qualities, such as transcendence, immanence, boundlessness, and ultimacy* (Pargament and Mahoney, 2005). *Virtually any part of life, positive or negative — including beliefs, practices, experiences, relationships, motivations, art, nature, and war — can be endowed with sacred status. […] By search, we are referring to an ongoing journey, a process that begins with the discovery of something sacred.”*

Their definition of *religion* is the following:

*“Building on the work of* Hill et al. (2000) and Pargament (1997), *we define religion as ‘The search for significance that occurs within the context of established institutions that are designed to facilitate spirituality.’ The term search refers once again to the ongoing journey of discovery, conservation, and transformation. In this case, however, the destination of the search is ‘significance’: a term that encompasses a full range of potential goals, including those that are psychological (e.g., anxiety reduction, meaning, impulse control), social (e.g., belonging, identity, dominance), and physical (e.g., longevity, evolutionary adaptation, death) as well as those that are spiritual* (Pargament, 1997; Mahoney and Thelen, 2010*; see Part II of this volume*)*. Religion occurs within the larger context of established institutions and traditions that have as their primary goal the facilitation of spirituality. In contrast to the modern tendency among scholars to dissociate spirituality from religion, our view is that spirituality represents the function most central to institutional religious life. It is the spiritual character of its mission that makes religious institutions distinctive; no other social institution has spirituality as its primary goal* (Mahoney and Thelen, 2010).*”*

Clearly, questions of “transcendence, immanence, boundlessness, and ultimacy” are recurring and perennial themes in some who report undergoing a process of emergence or non-developmental EPEEs. However, our phenomenological and clinical interests include a vast range of manifestations which are not always associated with these themes.

Many who experience emergent phenomena are indeed on a “quest,” and thus again there is some overlap with the notion of spirituality, but by all means, not all EPEEs represent “the discovery of something sacred” (Pargament et al., 2013) to those undergoing them, nor do they represent the beginning of a “process,” although, in the case of the “process of emergence,” as we conceive of it here, there is overlap.

Now, more crucially: it is true that each experience and effect described in the inventory could possibly be viewed or related to as *spiritual* in nature. But so could any aspect of life, as, per Pargament’s above definition, “virtually any part of life […] can be endowed with sacred status,” and spirituality is “the search for the sacred.” What is crucial to realize here is that, from this perspective, speaking of specifically spiritual experiences, with hypothetical essential phenomenal features, makes little sense, since the spiritual dimension of experiences depends entirely on *how one relates to them* at a reflexive level. Thus, strictly speaking, and using this definition, it follows that at the *pre-reflexive* level (Husserl, 2012; Vermersch, 2012, p. 138), there *are not necessarily any intrinsically spiritual experiences.* This label is one degree of elaboration too high. This is not in any way to disparage the use of such labels if they are of value, just to point out that, particularly when attempting to spread practical knowledge in various contexts, the insistence on the necessity of such labels may pose barriers to success.

This critique extends to all compounded expressions found in section 3.2 above, such as “spiritual emergence,” “spiritual experiences,” “spontaneous spiritual awakenings,” “spiritually transformative experiences,” etc.

Similarly, *religious* experience seems to be usually defined by its collective, social, and institutional context. This is a very common way of viewing religion. Yet, the same irresolvable contradiction immediately follows as in the previous paragraph. In theory, per Pargament’s definition, *virtually any experience could be seen as religious*, if related to as existing in a specifically collective/institutional context that seeks to produce significance and to foster the search for the sacred — spirituality — including shaking hands with someone or cutting one’s hair. In fact, this seems to be the case, as there are many practices broadly appraised as “religious” which entail exactly these sorts of actions (i.e., cutting one’s hair a certain way, or specific greeting ceremonies), and yet it seems difficult to relate these with the experiences and effects we speak about throughout the present article.

In short, the criteria that define spirituality and religion are not primarily phenomenological, but mostly *cultural* and *contextual*, related to *individual* and *collective meaning-making*, respectively. Meaning making is part of where *the specific value of spirituality and religion lies*. This is an important observation. It follows that, since our perspective is phenomenological, and our themes of interest are *specific experiences and effects*, we are interested in the pre-reflexive level associated with specific experiences and less about how they are related at the reflexive level. However, for our purposes here, we question the necessary and unquestioned use of labels such as spiritual or religious to broadly qualify this set of described and observed experiences and effects, because it:

- Dilutes the specific meaning of religion and spirituality,
- Sometimes adds confusing cultural overlays on EPEEs, and
- Impedes the globally scaling and practical application of a rich field of scientific and academic study and clinical practice to broad human detriment.

### 3.5.3 Emergence and mystical experience

As for mystical “states of consciousness,” the influence of William James’ seminal work, Var*ieties of Religious Experience*, and that of W.T. Stace in *Philosophy and Mysticism* is such, that theirs seems the most adequate source to look at. These conceptualizations are what modern scales such as the Mystical Experience Questionnaire (MEQ) and Mysticism Scale (M-scale) are based on, and these are the ones used in many clinical trials seeking to assess the effects and outcomes associated with psychedelic use; thus, their clinical relevance is significant.

Let us begin with James. Lectures XVI and XVII in the series which make up *The Varieties of Religious Experience* treat at length of the topic of “mystical phenomena” (p. 378) because, according to James, “personal religious experience has its root and center in mystical states of consciousness” (p. 379). Here, James sets himself the task of showing the “reality of the states in question,” and the “paramount importance of their function” (*ibid.*) He proposes a definition of the states comprised in the “mystical group” (p. 384) as characterized by “four marks”: (1) Ineffability, (2) Noetic quality, (3) Transiency and (4) Passivity (pp. 380–384).

In *Mysticism and Philosophy* (Stace, 1987), W.T. Stace provides a summary of the common characteristics of what he calls “extrovertive” and “introvertive” mystical experiences.

Extrovertive mystical experiences involve:

- “Unifying vision – all things are one,
- An apprehension of the One [“The Divine” or “Ultimate Reality”] as an inner subjectivity, or life, in all things,
- A sense of objectivity or reality,
- Blessedness, peace, [bliss], etc.
- Feeling of the holy, sacred, or divine,
- Paradoxicality, and
- Alleged ineffability.”

Introvertive mystical experiences involve:

- “The unitary consciousness – the One, the void [“The Divine” or “Ultimate Reality”];
- pure consciousness
- Non-spatial, non-temporal sense of objectivity or reality
- Blessedness, peace, [bliss], etc.
- Feeling of the holy, sacred, or divine
- Paradoxicality, and
- Alleged ineffability.”

The aforementioned scales directly derive from these two definitions. Indeed, the Mystical Experience Questionnaire (MEQ) comprises 30 or 43 question items, depending on the version, divided into 6 domains (Barrett et al., 2015). These are:

1. A sense of unity,
2. Transcendence of time and space,
3. Ineffability and paradoxicality,
4. A sense of sacredness,
5. A noetic quality, and.
6. A deeply-felt positive mood.

The Mysticism Scale (M-scale) comprises three domains (Streib et al., 2020):

1. Extrovertive, with 2 sub-domains of Inner Subjectivity and Unity,
2. Introvertive, with 3 sub-domains of Timelessness/Spacelessness, Ego loss, Ineffability, and.
3. Interpretation with 3 sub-domains of Positive affect, Sacredness, and a Noetic quality.

From our present perspective, critical comparison of these scale domains and James’ and Stace’s criteria with our inventory of contemporary scientific literature, as well as with traditional texts, reveals the profound limitations of the ways this notion is defined by these authors, with:

1. The *de facto* exclusion of experiences and effects of a challenging nature such as the “Dark Nights” — of the “Soul,” “Senses,” or “Spirit” (Kornfield, 1993; St. John of the Cross, 2010; Lutkajtis, 2019c), often tellingly called “Mystical Nights” — which certainly cannot be characterized by a positive mood, nor necessarily by a short duration, nor any of the other criteria we just listed. However, these are a common and essential phase of spiritual development according to canonical Christian texts. Notice, that there are *kinds* of dark nights. One may think this is specific to some Christian authors. But a similar point can be made with the Buddhist notion of the “*Dukkha ñanas*” (Pali), meaning “Knowledges of Suffering,” a normative developmental phase within the context of Theravada Buddhist meditation theory (Kornfield, 1993; Mahāsī Sayadaw, 1994; Grabovac, 2014; Lutkajtis, 2019a,b, 2020). As their name suggests, these “stages” of deepening meditation include experiences of “fear,” “misery,” “disgust,” etc. Yet, in this traditional framework, they are preceded and followed by stages which could fit many or all of the above criteria. Also, notice again that several variants and phases of “knowledges of suffering” are traditionally distinguished. Even if one chose to consider that later commentarial texts like the *Vissudhimagga* (Buddhaghosa, 1991) where this model was elaborately described and expanded on beyond its roots in the *Abhidhamma* and *Khuddaka Nikāya*, are “inauthentic,” one could respond that some texts which are canonical in *all* branches of Buddhism [the Pali canon (Ñanamoli and Bodhi, 1995)] contain ample evidence that “mystical experience” is not *just* what James and Stace described. Beyond these two traditions, grief and trauma (Yaden and Newberg, 2022) and generally speaking suffering-related experiences acting as triggers of spiritual development is a well-documented phenomenon. In fact, one may even say that in various traditional texts, mystical life begins with suffering and systematically involve negative experiences, sometimes to extreme degrees. This is reflected in Joseph Campbell’s *monomyth*, or Hero’s journey, a universal model of the unfolding of narrative and mythological stories in world literature, where the hero enters the Underworld or Abyss, or undergoes some profoundly challenging Quest, before coming back transformed or renewed to the known world (Campbell, 2008). Obviously, challenging EPEEs abound in our inventory, such as negatively valenced and sometimes terrifying psychedelic experiences (“bad trips”). Yet these may have long-term positive effects (as well as further negative effects) on one’s spirituality and life in general, be existentially and paradigmatically transformative, and result in beneficial long-term outcomes. All these examples show that we should not frame “mystical” experiences and generally EPEEs as only “pleasant” and ignore or fail to properly categorize and study non-pleasant, sometimes challenging EPEEs, at the risk of fueling what has been described as the “overly positive presentation of meditation in the media” (Lutkajtis, 2019c). One may argue that contemporary research has developed specific instruments to that effect (e.g., the Challenging Experience Questionnaire) but it is also clear that appraising all experiences which do not fit a simplistic definition of mystical experience as “challenging experiences,” “adverse effects” or “adverse events” (Farias et al., 2020), is problematic.
2. These criteria also exclude what some have termed “Interactive-relational” experiences, i.e., seeming encounters with various types of entities or presences — which do not necessarily involve a sense of inner or outer unity, nor any other of these criteria. Such experiences were one of the most common type of experiences across all modalities in our inventory [see, e.g., Greyson and Stevenson (1980), Hartley and Daniels (2008), Charland-Verville et al. (2014), Maqueda et al. (2015), Camlin et al. (2018), Vieten et al. (2018), Sparby (2020), and Lawrence et al. (2022)]. This also means a large portion of world literature, such as prophetic, oracular, or visionary texts; archetypal psychology (Jung, 1980; Hillman, 2004); the effects of many psychedelic compounds (such as LSD, 5-Meo-DMT, Ibogaine, N,N-DMT, or *Salvia Divinorum*); the effects of various meditation practices; and even spontaneously occurring such experiences, or these that are reported in various contexts like having an accident or going through coma — these would all be disqualified as mystical experience. Yet to experiencers, these may be felt as deeply “mystical,” profound, and transformative. More importantly, from a pragmatic, human, as well as clinical point of view, such experiences are often causal and may have a wide range of impacts on real world personal, family, and societal outcomes.
3. As we just showed, beyond excluding important experience types, Jamesian and Stacian characteristics of mystical experiences *lack differentiating power*, as they could in fact correspond to several forms of emergent phenomena which are distinguished both in Christian mystical theology, and Buddhist meditative theory. For instance, within the developmental models we just presented, these could correspond, within Carmelite theory of mystical development, either to the initial fruits of practice for beginning meditators before they enter the unpleasant and challenging purification of the Dark Nights, and/or to later phases of practice. Similarly, within buddhist stages of insight meditation, “mystical experience” fits well with either the very early phases of meditation, or to the all-important fourth stage of “Arising and Passing Away,” which immediately precedes the “*Dukkha ñanas*,” or to the one that immediately follows these “*Dukkha ñanas*” (called “Equanimity”). The 4, 8 or 9 *jhānas* would likely all qualify as mystical experiences, yet they each represent specific, differentiated experiences. It is highly likely that this comparison could be extended to almost all other mystical traditions. Thus, as with most current taxonomies of what we call EPEEs, recent definitions of mystical experience lack a sense of a differential diagnosis of possible states and stages that might present very similarly in some particulars but yet possibly be developmentally quite different.
4. Overall, the “transiency,” “ineffability,” “noetic,” and “passivity” criteria in James do not stand scrutiny with either traditional, or contemporary qualitative research. Indeed:

- The question of *ineffability*, might be an invalid criterion all together. Difficulty articulating a novel experience happening at the pre-reflexive level is a well-known fact of life. That is a basic premise of phenomenological research, with qualitative methods such as micro-phenomenology having been designed, precisely, to help experiences make “what experiences are like” explicit (Petitmengin, 2006; Husserl, 2012; Vermersch, 2012; Petitmengin et al., 2017; Petitmengin et al., 2019). The (perfectly legitimate) sense that talking about a given event would somehow diminish its value, leading to a form of privacy or secrecy, is not exactly the same thing as that experience being impossible to express.
- As for *transiency*, traditional knowledge and recent research shows that various meditative states, sometimes highly refined and rare, such as Buddhist *Nirodha Samāpatti* can last quite a long-time (in the case of NS, Laukkonen et al. (2023) report a duration of 90 min, and the commentarial literature, such as the *Visuddhimagga*, reports such attainments may last for 7 or more days). Further, if one considers the “Dark Nights” and “*Dhukka ñanas*” as variants of mystical states, then these could actually last for months, years, or decades.
- The *noetic* criterion — meaning the experience involves a sense of “knowing,” or of perceiving something “true” — although sometimes seemingly very valid, should be used with discernment, as it can be misleading or even deceptive, and some (though not all!) forms of “noesis” are commonly described as pitfalls in many traditions. For instance the “Arising and Passing Away” stage we mentioned is described in some Buddhist mediation manuals as involving the arising of a “*brilliant light*,” “strong mindfulness,” “**keen, strong, and lucid *knowledge***,” “strong *faith*,” “*rapture* in its five grades,” “*tranquillity* of mind,” “a very sublime feeling of *happiness*,” “*energy*,” “strong *equanimity*,” and “a subtle *attachment*” (Sayadaw, 1994). Yet these are clearly noted as “ten corruptions of insight” and “mistaking what is not the path for the path” (Sayadaw, 1994). The prescription here is to “purify” these corruptions by noticing that “The brilliant light, and the other things […] are not the path” and that “Delight in them is merely a corruption of insight” (Sayadaw, 1994). This applies to the noetic component, but also to other phenomenal features which are commonly attributed to mystical experiences by, e.g., Stace. Similar warnings around the early fruits of meditative practice can be found in Christian manuals (e.g., St. John of the Cross, 2010). In fact, classical mystical texts like the *Cloud of Unknowing*, would likely make the case that high mystical states rather involve a sense of *unknowing*, or *not knowing* (Wolters, 2001). The value of the sense of “knowing” in EPEEs is also questioned in contemporary research on psychedelic experiences (e.g., Timmermann et al., 2022 argue that “the ability of psychedelics to induce noetic feelings of revelation may enhance the significance and attribution of reality to specific beliefs, worldviews, and apparent memories […] might exacerbate the risk of iatrogenic complications that other psychotherapeutic approaches have historically faced, such as false memory syndrome”),
- The *passivity* criterion seems less ambiguous, but is nevertheless contradicted by the canonical description of, e.g., the first *jhāna*, which as we have seen involves intentionally “aiming” and “maintaining” one’s attention on the meditation object. Yet many would call the first jhāna a mystical experience.

The same terminological issues as outlined in section 3.2 apply, including:

*Idiosyncratic* use of the term “mystical.” Indeed, if we turn to classical early European mystical texts, such as those by late-antiquity author Plotinus, we find that they describe “mystical ascent,” the phases of which are described in Mazur et al. (2021), as culminating in a total “annihilation” into the “One” called *henosis* (Greek for “union”), which is followed by a “desubjectification.” Similarly, the last stage of the “path to God” as described by 13th-century German Mystics like Meister Eckhart or his student Tauler, is called a “modeless good” (De Libera, 1996), referring not as much to an “experience” with specific phenomenal features, but as a going beyond all manifest appearances (including going beyond feelings of bliss, of sacredness, of unity, of transcendence, etc.). Early mystical theologies like that of 5th- or 6th-century church father pseudo-Dionysius the Areopagite (Pseudo-Dionysius, 1987) — a ubiquitous influence on medieval contemplatives — and later ones such as that by Marler and Rorem (1996) and St. John of the Cross (2010), respectively describe *unio mystica*^34^ as “an obscure and luminous darkness” and a “going forth from the flesh.” The same is true of other Abrahamic traditions, as described in Fisher (2022). All of these quintessentially mystical experiences seem to bear more resemblance with the Buddhist notion of “cessation of consciousness” than with Jamesian or Stacean “mystical experience,” which likely should be correlated with phenomena and experiences encountered at earlier phases of these developmental models. Note that in Theravada texts, even such “cessations of consciousness” are classified into various types depending on their setup, mode of entrance, and phenomena and effects after the exit.

Furthermore, the term “mystical” is to some degree c*ulturally-bound.* Why would the Latin-originated expression of “mystical experience” be adequate to speak about the experience of Buddhist, Muslim, or Hinduist contemplatives? Why not use their terms instead? Cultural and historical boundaries must be respected, but it must also be acknowledged that insisting on using them in research, clinical or scientific contexts creates problems, not only culturally, but also regarding scaling globally into clinical, public health, and scientific contexts.

Additional history and criticism of these definitions and the psychometric instruments that derive from them can be found in Taves (2020). The author also reminds us that, “When William James criticized the contemptuous disregard with which scientists treated the ‘mass of phenomena generally called mystical’ in 1890, he presupposed a widely accepted definition of mysticism that included ‘*divinations, inspirations, demoniacal possessions, apparitions, trances, ecstasies, miraculous healings and productions of disease, and occult powers*’” (Taves, 2020). Furthermore, “James also linked such experiences with drugs” (Taves, 2020), writing that, “The drunken consciousness is one bit of the mystic consciousness,” and with certain “pathological conditions” (James, 2005). It is only later that he, alongside others, adopted a “narrower definition” (Taves, 2020), crystallizing in Stace’s limiting, contradictory, and biased definition of mysticism, which would inform 20th century thinking and research. Clearly, James’ original view of mysticism was closer to what we call EPEEs. Thus, in many ways, we are returning to the original Jamesian spirit.

In sum, we see EPEEs as a specific group that is differentiated from the notions of ASCs, religious, spiritual, or mystical experiences (in the modern sense) in specific and crucial ways that should not be overlooked.

Moreover, most classifications of EPEEs that we are aware of, usually lack precise differentiating criteria along various axes, including but not limited to:

- Sequence (what is described or predicted to occur before and after),
- context,
- Neurophysiological correlates,
- Micro-phenomenological details,
- Consequences,
- Function (capabilities gained, modified, or lost), and
- The possibility of polyphasic presentations and non-linear developmental trajectories that might untangle such possible sources of confusion.

All of these should be part of a more mature and realistic clinical perspective.

For these reasons and because of the complex history, ambiguities, and difficulty in finding consensual definitions of terms like ASCs, religious, spiritual, or mystical experiences, it appears to us more desirable to leave traditional (emic) terms in their traditional context. One might argue that perhaps the issues come from the specific definitions we chose, and that updating these would yield something less problematic. But it seems to us that the problems are in some ways intractable.

Given human’s deep needs for meaning, EPEEs often have been personally or socially interpreted in various ways. A given manifestation may be given various appraisals, e.g., a single EPEE may be alternatively interpreted as signs of “possession,” “kundalini syndrome,” “meditative attainment,” or “functional neurological disorder.” It is highly likely that these frames, whether sacralizing or pathologizing, have an impact on outcomes (Lutkajtis, 2020a).

We need a broad, overarching, adaptable, scalable framework that can approach EPEEs without mobilizing such specific lenses necessarily, and instead leave space for what happens in real clinical and social situations. As noted above, misunderstanding, questioning, or contradicting meanings assigned to EPEEs, or, conversely, not having normalizing meanings and taxonomic, descriptive, and diagnostic labels to offer, may have implications for the therapeutic or teaching relationship. Creating expansile, adaptable, scalable frameworks and teaching skills that allow the benevolent and sophisticated navigation of the range of meanings in an ethical and culturally sensitive way is broadly understood in clinical situations to promote good outcomes, which is our primary concern here.

The confusion which persists in contemporary research demonstrates how much work is needed in this area. This issue is made more pressing by the fact that many people are currently engaging in what we call emergent practices *outside* of any institutionalized context, e.g., outside a personal spiritual quest. Yet they *do* often stumble upon EPEEs, even when recreationally using psychedelic substances, or when seeking simple stress-reduction by engaging in mindfulness-based interventions or meditation apps. They may end-up lost and without a map — as may well be any teachers of emergent practices, clinicians, or other mental health professionals they encounter as part of this process. In this context, it is vital we seek to integrate spiritual, clinical, and scientific paradigms to promote beneficial outcomes and avoid harmful ones.

#### 3.5.4 The boundary question

An outstanding, critical concern, is about the exact boundaries of what qualifies as EPEEs or not.

At the “center,” where the effects are strong, and the impact significant and obvious, this is a relatively easy question. Dramatic “openings,” major “shifts,” powerful changes in perception: clearly these need to be included.

However, at the periphery, where, for example, for some, noticing thoughts are something they can observe might be a subtle shift (whereas it might be a dramatic revelation and/or existential challenge for others) and seem only slightly worthy of the designation “emergent phenomena.”

This is very much like defining the reasonable boundaries of the testable knowledge to be learned to be board certified in the medical field of, e.g., Emergency Medicine. From a certain point of view, care providers should know at least a little about nearly everything in medicine; yet, this being impossible, some lines must be drawn regarding what one can reasonably be required to know.

Thus, medical education focuses on core competencies, with the dual criteria that one must learn about the most frequently encountered problems, as well as those critical yet rare conditions that one might see once in a career or never but that it is absolutely required that one at least have some basic knowledge of. Cases falling outside this range may then be learned about with the assistance of a more knowledgeable colleague or specialist.

Similarly, for practical purposes, we begin at the relatively clear “center,” and will leave it for the progressing conversation to tease out which subtle effects are worthy of inclusion, from a clinical, pragmatic, and outcomes-based point of view — which likely be a long and complex conversation.

With this sort of pragmatism in mind, it seems possible to define a specialty focusing on EPEEs as an expansile, and perhaps, ultimately, somewhat vaguely bounded body of knowledge at the far edges.

## 4 Conclusion

In summary, we propose that our area of focus throughout this article constitutes a specific domain of human experience. EPEEs as we have defined them are not limited to any specific discipline, but could be variously approached through their physiological, phenomenological, anthropological, symbolic, functional, aesthetic, linguistic, and many other dimensions.

This is comparable to how fields like psychiatry cut across many disciplines, since psychiatric conditions and effects might involve numerous neurological, biochemical, social, functional, developmental, genetic/epigenetic, behavioral, metabolic, hormonal, symbolic, environmental, financial, legal, among other potential factors and consequences from many domains of concern both clinical and non-clinical which must be at least moderately well understood by numerous disciplines.

From a clinical perspective, we propose that the field of EPEEs, while potentially impacting and involving nearly every domain and system of medicine, is sufficiently complex and defined, likely requiring a unique enough set of skills and knowledge not adequately covered by existing specialties, to constitute its own specialty. We posit that the experiment of defining and creating this specialty would likely solve numerous problems currently faced by current research.

After years of careful discussion and reviews, exploration of ethical considerations and apparent mandates, and research, we believe in and advocate for using the term “emergence,” as well as its derivatives such as “emergent processes” and “emergent phenomena.”

We argue that these optimally balance scientific, philosophical, historic, anthropological, linguistic, conceptual, and globally clinically scalable considerations and so could be used as umbrella terms to describe this specific yet broad body of knowledge, research, and practice.

We understand and appreciate that advocating for specific terminological choices over this contentious field may invoke some challenging feelings and discussions, but we also feel strongly that the lack of such a non-problematic, precise, scalable, and appropriate umbrella terms has been such a significant impediment to the global study and scaling of this field as to be a substantial detriment to everyone involved in the process of emergence, and so feel this push is ethically warranted.

We recognize that this conclusion is reached by positing a set of optimal criteria which must be met (outlined in our introduction), and that this set of criteria itself is worthy of discussion and debate. We also look forward to the possibility that those more capable than us might come up with a term that not only meets all the criteria we propose, but also does this in a way that exceeds the term “emergence,” and invite those who care about this topic to engage in this ongoing conversation.

## Data availability statement

The original contributions presented in the study are included in the article/Supplementary material, further inquiries can be directed to the corresponding author.

## Author contributions

OS: Writing – original draft, Conceptualization, Data curation, Formal analysis, Methodology, Project administration, Supervision, Validation, Writing – review & editing. DI: Conceptualization, Funding acquisition, Resources, Supervision, Validation, Writing – review & editing.

## Footnotes

^1^For further explanations on the background and rationale for this addition, see Lukoff et al. (1995).

^2^E.g. “distressing experiences that involve loss or questioning of faith, problems associated with conversion to a new faith, or questioning of spiritual values that may not necessarily be related to an organized church or religious institution” (p. 835).

^3^For more information about these four pillars of medical ethics, see, e.g., Varkey (2021) or Beauchamp and Childress (1979).

^4^See for example the 1,000+ scientific articles and books we have gathered in a public Zotero collection we call the Emergence Research Database: https://www.zotero.org/ebresearch/collections/TAKNGM64.

^5^“Mystical union.”

^6^A greek term usually translated as a state “contemplation.”

^7^Following *Theoria, Theosis* is the culmination of the practice of *hesychasm* in orthodox Christianity and can be translated as “glorification.”

^8^A form of heightened awareness and vigilance.

^9^See note 8.

^10^An “intermediary absorptive state.”

^11^A “station” or “stage” on the “mystical path.”

^12^A “constriction.”

^13^Variously meaning a “vision,” “union,” the “annihilation” of the “self” or a “cessation of consciousness.”

^14^“Ecstasy.”

^15^Functional impairment or “madness” related with spiritual practice.

^16^Unification the field of consciousness, or concentration.

^17^This translates to “wheel,” and refers to important physiological “energetic centers” in traditional Hindu anatomy.

^18^A state, or stage, of liberation.

^19^States of increasingly deep absorption.

^20^Powers.

^21^Both are forms of cessation (Pali *nirodha*) of consciousness and feelings.

^22^Similar to the buddhist notion of access-concentration (Sayadaw, 1994), this refers to the “state of concentration necessary to successfully engage in specific contemplative practices, not just the result of them” (Fisher, 2022).

^23^This and following terms, respectively, mean “slumber,” “dream” and “vision” — all referring to a form of “prophetic” contemplation.

^24^A “place” of nothingness, “wherein all of one’s physical presence is transcended” (Fisher, 2022).

^25^“Nullification” or “annihilation,” a technical term relating to states of absorption” (Fisher, 2022).

^26^A radical change in life orientation.

^27^A greek term used by Plotinus to designate the highest level of “mystical ascent,” which translates literally to “union.”

^28^I.e. “a salient unity or intrinsic organization of diverse phenomenal features, based on reciprocal part-whole interactions” (Parnas, 2012).

^29^These are *vitakka* (applied attention), *vicāra* (sustained attention), *pīti* (rapture), *sukha* (joy), *ekaggata* (one-pointedness) and *upekkhā* for the fourth and higher *jhānas* (Hagerty et al., 2013) — each of these has equivalents in the inventory.

^30^On the spontaneous phenomenalization of our experiences into types, see Schuetz (1959) and Fernandez (2019).

^31^See https://theeprc.org.

^32^When studying a given ethnic group, anthropologists use the terms “emic” to refer to an attitude which mobilizes frameworks and views *internal* to a given cultural group, and “etic” when researchers instead use external concepts and notions.

^33^Doctrines of salvation, often in a Christian context, but also can be used for general contexts, as we do here.

^34^Mystical union.
