# Supplementary material for: Documenting and defining emergent phenomenology: theoretical foundations for an extensive research strategy
Source: Front Psychol. 2024 Jul 10;15:1340335. doi: 10.3389/fpsyg.2024.1340335 (PMC11304085; doi:10.3389/fpsyg.2024.1340335)
Supplement: Supplementary file 1 [file Data_Sheet_1.PDF]

# Synthesizing Models of Experience and Function

| Theoretical Phenomenology | | | | Qualitative Research | | | Biomedical Research | | Inventory Categories |
|---|---|---|---|---|---|---|---|---|---|
| Existentials as "phenomenological domains", "transcendental," "essential," or "ontological" structures of human subjectivity. Heidegger, 1962 (1) - Fernandez, 2019 (2) | Vion-Dury & Mougin, 2016 (3a), Le Blanc (3b) - Covariant and copresent "manners" or "presentations" or "guises" of the field of consciousness, which together constitute and characterize specific temporary "modalisations" | Husserl, "4 modes of consciousness", time consciousness, field of actuality and motivation (Bitbol, 2014, p. 128 *et seq.* (9a); De La Tremblaye & Bitbol, 2022, (9b); Husserl, 2019 (9c) | "Fundamental features of consciousness" - Parnas & Zahavi (7) | Seven "experiential domains" - Lindahl *et al.*, 2017 (4a) | Seven experiential "themes" in meditative experience - Sparby, 2020 (4b) | | Research Domain Criteria (RDoC) Functional Dimensions*** - Cuthbert & Insel, 2013 (5) - NIMH, 2023 (6) | | Phenomenal and Functional Dimensions of EPEEs**** (8) |
| | | | | | Main Category | Subcategory | Main Category | Subcategory | |
| Selfhood | Ipseity/Identity | | Self-awareness | Sense of Self | Self | | | | Existential > Sense of self |
| Intersubjectivity | | Welcoming (disposition to relate to alter egos) | Intersubjectivity | Social | Perception | Intersubjectivity (Subject-Subject perception) | 4) Systems for Social Processes | 4.4) Perception and Understanding of Others | Social |
| | | Co-presence | | | | | | 4.3) Perception and Understanding of Self | |
| | | | | | | | | 4.2) Social Communication | Collective |
| | | | | | | | | 4.1) Affiliation and Attachment | |
| Affectivity | Emotions | | | Affect | Affect | | | | Emotional |
| Understanding | Intellection | | | Cognition | Cognition | Knowledge<br>Meta-Cognition | 3) Cognitive Systems | | Cognitive > Meta-cognition / meta-awareness |
| | | | | | | Thinking | | | Cognitive > Thoughts |
| | Attention* | Attention | | | | Attention | | 3.1) Attention | Cognitive > Attentional |
| | Perception | | | Perception | Perception | Vision<br>Non-modality-specific perceptions<br>Auditory<br>Taste and Smell<br>Touch<br>Space perception<br>Synaesthesia | | 3.2) Perception | Perceptual |
| | Memory | Protentions | | | Cognition | Memory | | 3.3) Declarative Memory | Cognitive > Memory |
| | | Retentions | | | | | | 3.6) Working Memory | |
| | | Motivation | | Conation | | | | | Motivation |
| | | | | | | | | 3.5) Cognitive Control | Volitional |
| | | | | | | | | 3.4) Language | Expressive |
| | | | | | | | | | Semantic |
| Temporality | | Time-consciousness | Time-consciousness | | | | | | Temporal |
| | | Protentions | | | | | | | Sequential |
| | | Retentions | | | | | | | |
| Spatiality | | Field of actuality | | | | | | | Spatial |
| | | | | | | | | | Dimensional |
| Intentionality | | Intentional consciousness | Intentionality (object-directedness) | | | | | | Perception > Subject-to-object |
| | | | | | | | | | Perception > Subject-to-subject |
| | Impressional consciousness** | Impressional consciousness | Experiential awareness | | | | | | Sensate |
| | | | Embodiment | Somatic | Embodiment | | | | Existential > Sense of Embodiment |
| | | | | | | | | | Sensate > Proprioceptive/Kinesthetic |
| | | | | | | | | | Sensate > Tactile |
| | Imagination | | | | | | | | Sensate > Mental |
| | | | | | | | | | Perceptual > Mental |
| | | | | | | | 5) Arousal/Regulatory Systems | 5.1) Arousal | Arousal |
| | Vigilance* | Vigilance | | | Sleep | | | 5.3) Sleep and wakefulness | Wakefulness |
| | | | | | | | | 5.2) Circadian Rhythm | Physiological |
| | | | | | | | 1) Negative Valence Systems<br>2) Positive Valence Systems | | Hedonic Valence |
| | | | | | | | 6) Sensorimotor Systems | 6.1) Motor Actions | Motor/Behavioral |
| | | | | | | | | 6.2) Agency and ownership | Existential > Sense of Self > Agency |
| | | | | | | | | | Existential > Sense of Self > Sense of ownership |
| | | | | | | | | 6.3) Habit | Behavioral |
| | | | | | | | | 6.4) Innate motor patterns | Motor |
| | Interactions with the environment | | | | Environment | | | | Contextual |
| | | | | | Cognitive | Conviction | | | Paradigmatic (Self and worldviews) |
| | | | | | | | | | Functional |
| | | | | | | | | | Psychological |
| | | | | | | | | | Vocational |
| | | | | | | | | | Aesthetic/Numinous |
| | | | | | | | | | Energetic |
| | | | | | | | | | Sexual |
| | | | | | | | | | Informational |
| | | | | | | | | | Intuitive |
| | | | | | | | | | Archetypal/Visionary/Mythical/Symbolic |
| | | | | | | | | | Magical |
| | | | | | | | | | Medical |
| | | | | | | | | | Meta-emergent |

Text in blue are additions based on reviewing articles or books that document emergent phenomena and required novel categories.

* In Vion-Dury *et al.'s* model, attention and vigilance have special, regulatory functions, being both "regulators of modalisations of consciousness" - Attention is seen drawing on Husserlian phenomenology and ancludes Intentionality ; has a shape and width which will modulate the overall modalisation.and vice-versa.

** This term is used in opposition with "intentional consciousness" by Husserl in his *Lessons for a phenomenology of time-consciousness*, and refers to the "texture" or sensate "matter" (hylè) of all phenomena; Michel Henry later gave foundational importance to this notion in his so-called hyletic, material, or non-intentional phenomenology.

*** The "constructs" and domains of this framework are included based on two criteria : "there [are] two requirements for adding a construct to the matrix: first, 'There must be strong evidence for the validity of the suggested construct itself [as a behavioral function]'; second, 'There must be strong evidence that the suggested construct maps onto a specific biological system, such as a brain circuit'" (Cuthbert & Insel, 2013, p. 6). Thus it is far more limited in some ways than other models, since it is not descriptive of essential features of human subjectivity, but reflects "functional systems" with identified "biological correlates".

**** This scheme aims to integrate all previous models, thus covering both phenomenological and functional domains from e.g. classical/theoretical/transcendental phenomenology and biobehavioral fuinctional approaches like the RDoC. However, notice that it is in fact more comprehensive than a simple synthesis of all models presented here would be, and tailored to work for the analysis of our specific domain of interest.


**References** : (1) Heidegger, M. (1962). Being and time. (J. Macquarrie & E. Robinson, Trans.). New York: Harper Perennial Modern Classics - (2) Fernandez, A. V. (2019). Phenomenology and Dimensional Approaches to Psychiatric Research and Classification. Philosophy, Psychiatry, & Psychology, 26(1), 65–75. https://doi.org/10.1353/ppp.2019.0004 - (3a) Vion-Dury, J., Mougin, G.,. Modalisations of the consciousness field: a phenomenological and morphodynamic approach. PSN - psychiatrie, sciences humaines, neurosciences, 2016. hal-01580235 - (3b) Le Blanc-Louvry, I., (2015). Epaisseur spatialisante de la conscience. Thèse de Philosophie de l'Université de Rouen. - (4a) Lindahl, J.R., Fisher, N.E., Cooper, D.L. Rosen, R.K., Britton, W.B. (2017). The Varieties of Contemplative Experience: A Mixed-Methods Study of Meditation-Related Challenges in Western Buddhists. PLOS ONE 12(5): e0176239. https://doi.org/10.1371/journal.pone.0176239 - (4b) Sparby, T. (2020). Body, Soul, and Spirit: An Explorative Qualitative Study of Anthroposophic Meditation and Spiritual Practice. Religions, 11(6), 314. https://doi.org/10.3390/rel11060314 - (5) Cuthbert, B. N., & Insel, T. R. (2013). Toward the future of psychiatric diagnosis: The seven pillars of RDoC. BMC Medicine, 11(1), 126. https://doi.org/10.1186/1741-7015-11-126 - (6) NIMH » Development and definitions of the RDoC domains and constructs. (n.d.). Retrieved June 19, 2023, from https://www.nimh.nih.gov/research/research-funded-by-nimh/rdoc/development-and-definitions-of-the-rdoc-domains-and-constructs - (7) Parnas, J., & Zahavi, D. (2002). The Role of Phenomenology in Psychiatric Diagnosis and Classification. In M. Maj, W. Gaebel, J. J. López-Ibor, & N. Sartorius (Eds.), Psychiatric Diagnosis and Classification (pp. 137–162). John Wiley & Sons, Ltd. - (8) https://hypernotes.zenkit.com/i/UFIY1UO1cp/REKfkBv-1/emergent-phenomena?v=M6pP_Tb7W6 - (9a) Bitbol, M. (2014). *La conscience a-t-elle une origine ? Des neurosciences à la pleine conscience, une nouvelle approche de l'esprit*. Editions Flammarion. - (9b) De la Tremblaye, L., & Bitbol, M. (2022) Towards a phenomenological constitution of quantum physics: a QBist approach. *Mind and Matter*, 20 (1). pp. 35-62.
- (9c) Husserl, E., *The Phenomenology of Internal Time-Consciousness* (Second printing). (2019). Indiana University Press.
