# Supplementary material for: Documenting and defining emergent phenomenology: theoretical foundations for an extensive research strategy
Source: Front Psychol. 2024 Jul 10;15:1340335. doi: 10.3389/fpsyg.2024.1340335 (PMC11304085; doi:10.3389/fpsyg.2024.1340335)
Supplement: Supplementary file 2 [file Data_Sheet_2.PDF]

A General Inventory of the Emergent Phenomena, Experiences, Effects, After-effects and Impacts which are Documented in Recent Scientific and Clinical Literature

Table of References

Meditation Publications[1]	Type	Practice	N =
[a] Aizik-Reebs, A., Shoham, A., & Bernstein, A. (2021). First, do no harm: An intensive experience sampling study of adverse effects to mindfulness training. *Behaviour Research and Therapy*, *145*, 103941. https://doi.org/10.1016/j.brat.2021.103941	Clinical study using experience sampling	Mindfulness	71
[b] Ataria, Y., Dor-Ziderman, Y., & Berkovich-Ohana, A. (2015). How does it feel to lack a sense of boundaries? A case study of a long-term mindfulness meditator. *Consciousness and Cognition*, 37, 133–147. https://doi.org/10.1016/j.concog.2015.09.002	Case study (elicitation interview method)	Mindfulness	1
[c] Britton, W. B., Lindahl, J. R., Cahn, B. R., Davis, J. H., & Goldman, R. E. (2014). Awakening is not a metaphor: the effects of Buddhist meditation practices on basic wakefulness. *Annals of the New York Academy of Sciences*, 1307, 64–81. https://doi.org/10.1111/nyas.12279	Review	Buddhist meditation NOS[2]	N/A
[d] Castillo, R. J. (1990). Depersonalization and Meditation. *Psychiatry Interpersonal & Biological Processes*, 53(2), 158–168. https://doi.org/10.1080/00332747.1990.11024497	Review + Qualitative interviews	Transcendental meditation; Concentration on visual object	6
[e] Cooper, D. J., Lindahl, J. R., Palitsky, R., & Britton, W. B. (2021). "Like a Vibration Cascading through the Body": Energy-Like Somatic Experiences Reported by Western Buddhist Meditators. *Religions*, *12*(12), 1042. https://doi.org/10.3390/rel12121042	Qualitative study (semi-structured interviews)	Buddhist meditation NOS	42
[f] Easterlin, B. L., & Cardeña, E. (1998). Cognitive and Emotional Differences between Short- and Long-Term vipassanā Meditators. *Imagination, Cognition and Personality*, 18(1), 69–81.	Qualitative study (experience sampling)	Meditation NOS	43
[g] Farias, M., Maraldi, E., Wallenkampf, K. C., & Lucchetti, G. (2020). Adverse events in meditation practices and meditation-based therapies: a systematic review. *Acta Psychiatrica Scandinavica*, 142(5), 374–393. https://doi.org/10.1111/acps.13225	Systematic review	Meditation NOS	6,703
[h] Full, G. E., Walach, H., & Trautwein, M. (2013). Meditation-Induced Changes in Perception: An Interview Study with Expert Meditators (Sotapannas) in Burma.	Qualitative study (semi-structured	Theravadā śamatha and	18

[1] One can note that contrary to psychedelic research, what exactly is being done to produce effects is ill defined, and sometimes, a given article will compound vastly different practice style into a single "meditation" category. Another limitation is that there is an under-representation of Christian, Islamic, Jewish, and other practices, in favor of Buddhist and mindfulness-type practices.

Mindfulness, 4(1), 55–63. https://doi.org/10.1007/s12671-012-0173-7	interviews)	vipassanā Meditation	
[i] Grabovac, A. (2015). The Stages of Insight: Clinical Relevance for Mindfulness-Based Interventions. *Mindfulness*, *6*(3), 589–600. https://doi.org/10.1007/s12671-014-0294-2	Review/theory	Buddhist meditation NOS	N/A
[j] Hanley, A. W., Dambrun, M., & Garland, E. L. (2020). Effects of Mindfulness Meditation on Self-Transcendent States: Perceived Body Boundaries and Spatial Frames of Reference. *Mindfulness*, 11(5), 1194–1203. https://doi.org/10.1007/s12671-020-01330-9	Clinical trial for NPI	Mindfulness meditation	45
[k] Kaselionyte, J., & Gumley, A. (2017). “It’s like a charge – either fuses you or burns you out”: an interpretative phenomenological analysis of extreme mental states in meditation context. *Mental Health, Religion & Culture*, *20*(10), 986–1001. https://doi.org/10.1080/13674676.2017.1422237	Qualitative study (Interpretative Phenomenological Analysis)	Meditation NOS	3
[l] Kaselionyte, J., & Gumley, A. (2019). Psychosis or spiritual emergency? A Foucauldian discourse analysis of case reports of extreme mental states in the context of meditation. *Transcultural Psychiatry*, *56*(5), 1094–1115. https://doi.org/10.1177/1363461519861842	Systematic review of case reports + Foucaldian discourse analysis of case reports	Meditation (various): Transcendental Meditation; Qigong; yoga NOS; Arica meditation; Bikram yoga; siddha yoga; pranic healing meditation; kundalini meditation; kundalini yoga; zen meditation; unspecified.	32
[m] Kuijpers, H. J. H., Van Der Heijden, F. M. M. A., Tuinier, S., & Verhoeven, W. M. A. (2007). Meditation-Induced Psychosis. *Psychopathology*, 40(6), 461–464. https://doi.org/10.1159/000108125	Case study	Hindu Meditation NOS	1
[n] Laukkonen, R. E., Sacchet, M. D., Barendregt, H., Devaney, K. J., Chowdhury, A., & Slagter, H. A. (2023). Cessations of consciousness in meditation: Advancing a scientific understanding of nirodha samāpatti. In *Progress in Brain Research* (p. S0079612322001984). Elsevier. https://doi.org/10.1016/bs.pbr.2022.12.007	Case study	Buddhist śamatha/vipassanā meditation	1
[o] Lindahl JR, Kaplan CT, Winget EM, Britton WB. (2014). A phenomenology of meditation-induced light experiences: Traditional Buddhist and neurobiological perspectives. *Frontiers in Psychology*, 4 : 973	Qualitative study (semi-structured	Buddhist meditation NOS	28

https://doi.org/10.3389%2Ffpsyg.2013.00973	interviews)		
[p] Lindahl, J.R., Fisher, N.E., Cooper, D.L. Rosen, R.K., Britton, W.B. (2017). The Varieties of Contemplative Experience: A Mixed-Methods Study of Meditation-Related Challenges in Western Buddhists. *PLOS ONE* 12(5): e0176239. https://doi.org/10.1371/journal.pone.0176239	Qualitative study (semi-structured interviews)	Buddhist meditation NOS	60+32
[q] Lindahl, J. R., & Britton, W. B. (2019). "I have this feeling of not really being here": Buddhist meditation and changes in sense of self. *Journal of Consciousness Studies*, 26, 157–183.	Qualitative study (semi-structured interviews)	Buddhist meditation NOS	49
[r] Nave, O., Trautwein, F.-M., Ataria, Y., Dor-Ziderman, Y., Schweitzer, Y., Fulder, S., & Berkovich-Ohana, A. (2021). Self-Boundary Dissolution in Meditation: A Phenomenological Investigation. *Brain Sciences*, *11*(6), 819. https://doi.org/10.3390/brainsci11060819	Qualitative study (micro-phenomenological interviews)	Self-boundary Dissolution Meditation	46
[s] Poulin, M. J., Ministero, L. M., Gabriel, S., Morrison, C. D., & Naidu, E. (2021). Minding Your Own Business? Mindfulness Decreases Prosocial Behavior for People With Independent Self-Construals. *Psychological Science*, 32(11), 1699–1708. https://doi.org/10.1177/09567976211015184	Qualitative study (structured questionnaire)	Mindfulness	691
[t] Sparby, T. (2019). Phenomenology and Contemplative Universals: The Meditative Experience of Dhyana, Coalescence, or Access Concentration. *Journal of Consciousness Studies* 26 (7-8):130-156.	Qualitative case study (micro-phenomenological self-interview)	Jhāna meditation	1
[u] Sparby, T. (2020). Body, Soul, and Spirit: An Explorative Qualitative Study of Anthroposophic Meditation and Spiritual Practice. *Religions*, 11(6), 314. https://doi.org/10.3390/rel11060314	Qualitative study (semi-structured interviews)	Anthroposophic meditation	30
[v] Vieten, C., Wahbeh, H., Cahn, B. R., MacLean, K., Estrada, M., Mills, P., Murphy, M., Shapiro, S., Radin, D., Josipovic, Z., Presti, D. E., Sapiro, M., Chozen Bays, J., Russell, P., Vago, D., Travis, F., Walsh, R., & Delorme, A. (2018). Future directions in meditation research: Recommendations for expanding the field of contemplative science. *PLOS ONE*, 13(11), e0205740. https://doi.org/10.1371/journal.pone.0205740	Qualitative study (online survey)	Meditation NOS	1,120
[w] Wahbeh, H., Sagher, A., Back, W., Pundhir, P., & Travis, F. (2018). A Systematic Review of Transcendent States Across Meditation and Contemplative Traditions. *EXPLORE*, *14*(1), 19–35. https://doi.org/10.1016/j.explore.2017.07.007	Systematic review	Christian Contemplative Prayer; Christian practice NOS; Transcendental Meditation;	672

		Vedic Yoga; vipassanā; Buddhist practice NOS; Loving kindness; other NOS	
			Sub-total N = 9603-119 = 9484[3]

Psychedelics Publications	Type	Compound	N =
[1] Barnett, B. S., Sweat, N. W., & Hendricks, P. S. (2023). Case report: Prolonged amelioration of mild red-green color vision deficiency following psilocybin mushroom use. *Drug Science, Policy and Law*, *9*, 205032452311725. https://doi.org/10.1177/20503245231172536	Case report	Psilocybin mushrooms	1
[2] Barrett, F. S., Bradstreet, M. P., Leoutsakos, J.-M. S., Johnson, M. W., & Griffiths, R. R. (2016). The Challenging Experience Questionnaire: Characterization of challenging experiences with psilocybin mushrooms. *Journal of Psychopharmacology (Oxford, England)*, *30*(12), 1279–1295. https://doi.org/10.1177/0269881116678781	Qualitative study (online survey)	Psilocybin mushrooms	1,993
[3] Bouso, J. C., Andión, Ó., Sarris, J. J., Scheidegger, M., Tófoli, L. F., Opaleye, E. S., Schubert, V., & Perkins, D. (2022a). Adverse effects of ayahuasca: Results from the Global Ayahuasca Survey. *PLOS Global Public Health*, 2(11), e0000438. https://doi.org/10.1371/journal.pgph.0000438	Qualitative study (online survey)	Ayahuasca	10,836
[4] Breeksema, J. J., Kuin, B. W., Kamphuis, J., van den Brink, W., Vermetten, E., & Schoevers, R. A. (2022). Adverse events in clinical treatments with serotonergic psychedelics and MDMA: A mixed-methods systematic review. *Journal of Psychopharmacology* (Oxford, England), 36(10), 1100–1117. https://doi.org/10.1177/02698811221116926	Systematic review	Serotonergic psychedelics and MDMA	598
[5] Breeksema, J. J., Niemeijer, A., Kuin, B., Veraart, J., Kamphuis, J., Schimmel, N., van den Brink, W., Vermetten, E., & Schoevers, R. (2022b). Holding on or letting go? Patient experiences of control, context, and care in oral esketamine treatment for treatment-resistant depression: A qualitative study. *Frontiers in Psychiatry*, 13, 948115. https://doi.org/10.3389/fpsyt.2022.948115	Qualitative study (semi-structured interviews)	Esketamine	17
[6] Camlin, T. J., Eulert, D., Thomas Horvath, A., Bucky, S. F., Barsuglia, J. P., & Polanco, M. (2018). A phenomenological investigation into the lived experience of ibogaine and its potential to treat opioid use disorders. *Journal of Psychedelic Studies*, *2*(1), 24–35. https://doi.org/10.1556/2054.2018.004	Qualitative study (semi-structured interviews)	Ibogaine	10

[3] Studies [e], [p], [q] and perhaps [o] were conducted based on the interviews of a single cohort, so we will consider that their aggregated contribution is N=93 and subtract 119 from the sub-total.

[7] Carbonaro, T. M., Bradstreet, M. P., Barrett, F. S., MacLean, K. A., Jesse, R., Johnson, M. W., & Griffiths, R. R. (2016). Survey study of challenging experiences after ingesting psilocybin mushrooms: Acute and enduring positive and negative consequences. *Journal of Psychopharmacology*, *30*(12), 1268–1278. https://doi.org/10.1177/0269881116662634	Qualitative study (online survey)	Psilocybin mushrooms	1,993
[8] Colbert, R., & Hughes, S. (2023). Evenings with Molly: Adult Couples' Use of MDMA for Relationship Enhancement. *Culture, Medicine, and Psychiatry*, *47*(1), 252–270. https://doi.org/10.1007/s11013-021-09764-z	Qualitative study (In-depth unstructured interviews)	MDMA	16
[9] Dos Santos, R., Enyart, S., Bouso, JC., Pares, Ò., Hallak, J. (2018). "Ayahuasca turned on my mind's eye": Enhanced visual imagery after ayahuasca intake in a man with "blind imagination" (aphantasia). Journal of Psychedelic Studies. 2. 1-4. 10.1556/2054.2018.008.	Case report	Ayahuasca	1
[10] Kurtom, M., Henning, A., & Espiridion, E. D. (n.d.). Hallucinogen-persisting Perception Disorder in a 21-year-old Man. *Cureus*, 11(2), e4077. https://doi.org/10.7759/cureus.4077	Case Report	LSD	1
[11] Lawrence, D. W., Carhart-Harris, R., Griffiths, R., & Timmermann, C. (2022). Phenomenology and content of the inhaled N, N-dimethyltryptamine (N, N-DMT) experience. *Scientific Reports*, *12*(1), 8562. https://doi.org/10.1038/s41598-022-11999-8	Qualitative study based on spontaneous self-reports	N,N-DMT	3,778
[12] Liu, Y., Lin, D., Wu, B., & Zhou, W. (2016). Ketamine abuse potential and use disorder. *Brain Research Bulletin*, 126(Pt 1), 68–73. https://doi.org/10.1016/j.brainresbull.2016.05.016	Review	Ketamine	N/A
[13] Lutkajtis, A., & Evans, J. (2023). Psychedelic integration challenges: Participant experiences after a psilocybin truffle retreat in the Netherlands. *Journal of Psychedelic Studies*, 6(3), 211–221. https://doi.org/10.1556/2054.2022.00232	Qualitative study (semi-structured interviews)	Psilocybin truffle	9
[14] Mallaroni, P., Mason, N. L., Reckweg, J. T., Paci, R., Ritscher, S., Toennes, S. W., Theunissen, E. L., Kuypers, K. P. C., & Ramaekers, J. G. (2023). Assessment of the Acute Effects of 2C-B vs. Psilocybin on Subjective Experience, Mood, and Cognition. *Clinical Pharmacology & Therapeutics*, *114*(2), 423–433. https://doi.org/10.1002/cpt.2958	Double blind, placebo controlled study	2C-B	22
[15] Maqueda, A. E., Valle, M., Addy, P. H., Antonijoan, R. M., Puntes, M., Coimbra, J., Ballester, M. R., Garrido, M., González, M., Claramunt, J., Barker, S., Johnson, M. W., Griffiths, R. R., & Riba, J. (2015). Salvinorin-A Induces Intense Dissociative Effects, Blocking External Sensory Perception and Modulating Interoception and Sense of Body Ownership in Humans. *International Journal of Neuropsychopharmacology*, *18*(12), pyv065. https://doi.org/10.1093/ijnp/pyv065	Randomized placebo-controlled trial, qualitative data obtained through spontaneous reports	Salvinorin-A	8

[16] McNamee, S., Devenot, N., & Buisson, M. (2023). Studying Harms Is Key to Improving Psychedelic-Assisted Therapy—Participants Call for Changes to Research Landscape. *JAMA Psychiatry*. https://doi.org/10.1001/jamapsychiatry.2023.0099	N/A	N/A	N/A
[17] Palhano-Fontes, F., Barreto, D., Onias, H., Andrade, K. C., Novaes, M. M., Pessoa, J. A., Mota-Rolim, S. A., Osório, F. L., Sanches, R., Dos Santos, R. G., Tófoli, L. F., de Oliveira Silveira, G., Yonamine, M., Riba, J., Santos, F. R., Silva-Junior, A. A., Alchieri, J. C., Galvão-Coelho, N. L., Lobão-Soares, B., … Araújo, D. B. (2019). Rapid antidepressant effects of the psychedelic ayahuasca in treatment-resistant depression: a randomized placebo-controlled trial. *Psychological Medicine*, 49(4), 655–663. https://doi.org/10.1017/S0033291718001356	Parallel-arm, double blind randomized placebo-controlled trial, qualitative data obtained through clinical scales	Ayahuasca	29
[18] Ranganathan, M., Schnakenberg, A., Skosnik, P. D., Cohen, B. M., Pittman, B., Sewell, R. A., & D'Souza, D. C. (2012). Dose-Related Behavioral, Subjective, Endocrine, and Psychophysiological Effects of the κ Opioid Agonist Salvinorin A in Humans. *Biological Psychiatry*, *72*(10), 871–879. https://doi.org/10.1016/j.biopsych.2012.06.012	Double-blind, randomized, crossover, counterbalanced study, qualitative data obtained through clinical scales	Salvinorin-A	10
[19] Simonsson, O., Hendricks, P. S., Chambers, R., Osika, W., & Goldberg, S. B. (2023). Prevalence and associations of challenging, difficult or distressing experiences using classic psychedelics. *Journal of Affective Disorders*, 326, 105–110. https://doi.org/10.1016/j.jad.2023.01.073	Qualitative study (survey)	Classic psychedelics	250
[20] Srirangam, S., & Mercer, J. (2012). Ketamine bladder syndrome: an important differential diagnosis when assessing a patient with persistent lower urinary tract symptoms. *BMJ Case Reports*, 2012, bcr2012006447. https://doi.org/10.1136/bcr-2012-006447	Clinical case report	Ketamine	1
[21] Timmermann, C., Kettner, H., Letheby, C., Roseman, L., Rosas, F. E., & Carhart-Harris, R. L. (2021). Psychedelics alter metaphysical beliefs. *Scientific Reports*, 11(1), 22166. https://doi.org/10.1038/s41598-021-01209-2	Qualitative study (prospective online survey)	Psychedelics NOS	536[4]
[22] Wardle, M. C., & De Wit, H. (2014). MDMA alters emotional processing and facilitates positive social interaction. *Psychopharmacology*, 231(21), 4219–4229. https://doi.org/10.1007/s00213-014-3570-x	Double-blind controlled clinical trial, qualitative data obtained through clinical scales	MDMA	36

[4] We excluded the participants who had never used psychedelics (N=330).

Sub-total N = 20,145

Other/Spontaneous	Type	Cause/Context	N=
[α] Charland-Verville, V., Jourdan, J.-P., Thonnard, M., Ledoux, D., Donneau, A.-F., Quertemont, E., & Laureys, S. (2014). Near-death experiences in non-life-threatening events and coma of different etiologies. *Frontiers in Human Neuroscience*, *8*, 203. https://doi.org/10.3389/fnhum.2014.00203	Retrospective qualitative study	Coma/Accident NOS	190
[β] Greyson, B., Broshek, D. K., Derr, L. L., & Fountain, N. B. (2015). Mystical experiences associated with seizures. *Religion, Brain & Behavior*, *5*(3), 182–196. https://doi.org/10.1080/2153599X.2014.895775	Qualitative study (spontaneous reports + questionnaires)	Seizure	100
[γ] Greyson, B., & Stevenson, I. (1980). The phenomenology of near-death experiences. *American Journal of Psychiatry*, *137*(10), 1193–1196.	Retrospective qualitative study (spontaneous reports, questionnaires, interviews and medical records)	Being near death (NDE)	78
[δ] Hartley, J., & Daniels, M. (2008). A grounded theory investigation into negative paranormal or spiritual experience, based on the 'diabolical mysticism' of William James. *Transpersonal Psychology Review*, *12*(1), 51–72.	Qualitative study (semi-structured interviews)	Spontaneous NOS	7
[ε] Taylor, S. (2022). When Seconds Turn Into Minutes: Time Expansion Experiences in Altered States of Consciousness. *Journal of Humanistic Psychology*, *62*(2), 208–232	Qualitative study (spontaneous reports with some follow ups)	Accident/Emergency NOS	96
Sub-total N = 471			
Total N = 30,100			

For each item in the below inventory inventory, the article which reports it is referenced in parentheses after the item, along with the associated hypothetical cause. If several publications reported similar-enough phenomenology to warrant the merging of two descriptors or types into a single element, then the reference numbers and modalities were all listed within a single parenthesis after the given, common effect-type.

We invite the reader interested in exploring the phenomenology reported in specific articles to search the present document (ctrl/cmd+*f*) using the article of interest's unique reference number (*i.e.* [x]). This will allow one to navigate the entire document easily.

Introductory remarks

As explained in its related article, this inventory represents the synthesis of qualitative data found in the 50 publications listed in the above table, which taken together amount to a sample of 30,100 participants. The present section expands the discussion on factors which limit the results contained in this inventory.

Quality of research

Much clinical or qualitative research on these experiences is of relatively low quality, with many using structured questionnaire-type approaches, which are often quite "inductive" of content, imprecise, and general. Structured interviews are often a reflection of the researcher's own phenomenological expectations and interpretations and wording which may not always adequately reflect a person's actual unique experience. They allow "quantitative" analysis (statistical methods can be used on questionnaire responses), but rarely provide rich phenomenological descriptions, which is what we are looking for here. Some papers included here are of better quality, but the lack of uniformity of the literature is apparent.

Many do not at all address the question of distinguishing between "experience" and "interpretation[5]". They rarely include procedures to attempt to bypass this (*i.e.*, to induce a state of *époché* or suspension of preconceptions and interpretations (Bitbol, 2019) in their subjects), and much less so for the researchers themselves. This stands in stark contrast with methods such as the micro-phenomenological interview, which should be considered of higher phenomenological quality, as they (1) avoid suggesting content; (2) induce a state of *époché* in both the interviewer and interviewee; (3) explore the fine, pre-reflexive details of specific experiences in great depth, and (4) only later, after having explored specific experiences with several interviewees, proceed at attempting to identify structural invariants in individual descriptions and then general structures (Petitmengin, 2006 ; Petitmengin *et al.*, 2019). Questionnaire items based on such in-depth phenomenological descriptions could be developed and would probably be of much higher quality.

A case-in-point here is Castillo (1990) — a qualitative exploration of depersonalization/derealization experiences in meditation practitioners. Although a very interesting text, this article, which extensively quotes direct interview excerpts, exhibits a strong disparity between the experience reports and the labels or appraisal assigned to them. For instance, one hardly sees why statements such as: (1) "things take on a slightly warmer and glossier appearance — like dew," (2) "there is a definite aliveness in the environment, almost as if you're aware that there is consciousness in everything", and (3) "it's like there's all kind of little moving things. Objects are not as solid. They're more like millions of little spots" should all be construed as experiences of "derealization", and yet that is what the article does. This is one of the reasons why, whenever an article quoted direct interview excerpts, we reanalyzed these directly.

RCTs in particular are often very bad in terms of phenomenology, their analysis is often stereotypical and lacks subtlety, as they typically employ psychometric scales as outcome measures. In such cases, we learn little about the actual phenomenology of the experiences of the study participants, since a scale is a

[5] This is considered an important distinction in phenomenological research and was also stressed by *e.g.* Stace in his work on "mystical experiences."

predetermined binary yes/no phenomenological matrix. This makes some sense, since RCTs are about testing causal hypotheses and thus need simple categories that can be quantified and subjected to various statistical methods. However, it seems to us that many of the psychometric instruments used in such research lack phenomenological subtlety and depth. Reviewing existing scales and showing the limitations that affect most of them would be an interesting project to take on, especially because clinical trials for novel agents are currently being conducted mostly using such questionnaires.

Phenomenological and meditative expertise

The question of the phenomenological expertise of the subjects and/or researchers — that is to say, the expertise in verbally describing experiences at the pre-reflexive level — is also rarely addressed in existing research. Thus, it is difficult to know if different people mean the same thing by *e.g.* "altered sense of identity" or "body parts disappearing" when they are responding "yes" to such a question, and what their degree of phenomenological expertise is.

Some first- and second-person methods are "powerful" (Olivares *et al.*, 2015), but conducting interviews requires skill on the part of the interviewer to yield good results, just as many scientific experiments require training on the part of the person conducting them. This has rarely been pointed out, as such methods are relatively new, but there are many similarities between the micro-phenomenological interview method and the clinical interview (see the *Bates' Guide To Physical Examination And History Taking* chapter on this topic [Bickley *et al.*, 2017]), although the former is usually deeper and finer-grained. It follows that one may, in fact, suppose that clinical case reports will tend to contain better qualitative data than poorly done, questionnaire-based qualitative studies. From a modern medical perspective though, all of this remains at the level of anecdotal evidence.

In the case of meditation research, the question of the meditative expertise of subjects is also rarely addressed, but it plays a crucial role. It takes training to become adept at adequately describing experiences without filtering them through pre-interpretations, theories, vernacular language, or beliefs (Petitmengin *et al.*, 2017).

Our experience has been that many publications which include direct quotes of experience reports often either (1) analyze them in a misleading way, or (2) overlook aspects (sometimes many, and sometimes important) of the information contained in the description. We have not systematically assessed the quality of research, but this would be a worthy line of further work on the present data.

Thus, without modifying existing analytical contents, we have sometimes added new descriptive elements based on qualitative quotes included in publications which had gone unanalyzed. Finally, we have also restructured the thematic categories that were presented in most publications into a unified, more detailed, thematic framework.

It would be beneficial if researchers and participants underwent some training in phenomenological and qualitative methods in future clinical research.

Valuation vs. Pleasantness: the consequences of assessing specific phenomena as "adverse-events"

Most research focuses on descriptions without necessarily assessing the overall interpretation and valuation of the experience by the subject, either in the short, medium, or the long term (which should be the case in future research). For instance, an *unpleasant* experience that was initially related to as abnormal or "adverse," may later be re-appraised as overall valuable, desirable, or salutary by the same person. Conversely, some pleasant experiences may eventually be appraised as negative and undesirable either by the practitioner, their teachers, mental health practitioners, family members, friends, community members, or by their or another tradition.

There can even be a non-linear developmental trajectory to the way a specific experience is related to. In fact, many contemplative and philosophical traditions (such as Stoicism, Buddhism, Christianity, etc.) stress the importance of actively transforming one's view of or relationship to challenging experiences of this kind (see Sparby, 2022).

Furthermore, several authors have pointed out that the way a specific event is appraised or valued, either during or after it, can have consequences on outcomes (Timmermann et al., 2022; Lindahl *et al.*, 2022a and 2022b).

Causal attribution

Reports here are associated by participants to some practice they engaged in. The causal attribution should of course be seen with a dose of healthy skepticism, since practitioners do not usually systematically attempt to determine causal relationships. Obviously, for the person experiencing these, strong immediate temporal associations with practices and circumstances are convincing evidence for causality, often reported to have a “noetic” quality — that of certain knowledge — and, while this may be so in fact, a perceived sense of certainty should not necessarily be mistaken for an actual certainty of attribution or causation.

Breaking down experiences into their components: Phenomena, *Gestalts*, Signs, and Symptoms

An issue which arises when breaking things down into components like we do here is that one loses the "gestalt" quality of specific experiences, which present multiple co-occurring dimensions interwoven in a single whole which, in reality, cannot be separated and are integral to it. Although most of the experiences we are concerned with are not psychiatric *per se*, it is still helpful to mobilize the following notion:

> "The notion of Gestalt refers to a salient unity or intrinsic organization of diverse phenomenal features, based on reciprocal part-whole interactions. In this framework, psychiatric symptoms and signs cannot be considered as mutually independent, atomic features that become individuated (i.e., identified as this or that particular symptom) ‘in themselves,’ independently of their experiential context (Parnas, 2012, p. 67).”

Within the field of psychiatry, approaching patients' experience in this way is referred to as the *core gestalt* approach.

However, it seems possible to us that complex, multidimensional experiences can, for certain purposes, such as neurophenomenology, underlying physiology, and practical systems of taxonomy, be analyzed into several aspects and elements, while respecting the integrity of a given whole experience, and, in fact doing so may help clarify which of the phenomenal features of a "gestalt" actually are. As Fernandez (2019) argues: "[...] A phenomenological-dimensional approach can complement and support a core gestalt approach [...]. Conceptually distinguishing domains and dimensions of human subjectivity as a guide for phenomenological research does not entail that these domains and dimensions are separate from each other, or otherwise unrelated to a holistically organized subjectivity."

For instance, the following report…

> "Another thing was that I didn’t feel like I had any personal space — like, a sense of a boundary-ness. [...] If you came close to me, I’d just feel you in a painful way — it’s like you’re inside of me or something. So if anybody had any feelings or emotions... Of course, I was too whacked out to be able to tell what they were, but I could feel it. So I was very permeable in that way. It was not a pleasant experience, either". (Lindahl and Britton, 2019)

…May be seen as comprising several thematic aspects, such as:

(1) changes to *existential phenomenal features*, *i.e.* the sense of boundaries between "self" and "world" — feeling "permeable" ("I didn’t feel like I had any personal space - like, a sense of a boundary-ness"), feeling like others are inside oneself ("it’s like you’re inside of me or something"),

(2) increased *emotional and perceptual sensitivity* — *i.e.* increased empathy ("if anybody had any feelings or emotions [...] I could feel it") and sensitivity (to the point of being painful: "If you came close to me, I’d just feel you in a painful way"),

(3) changes related with arousal (hypoarousal) ("I was [...] whacked out"), although this interpretation should be confirmed by more contextual information, as implying tiredness from “whacked out” might be erroneous, and, instead, might imply some strong alteration, confusion, disorientation, etc.

(4) *psychological* dimensions — *i.e.* lack of insight into emotional percepts ("I was too whacked out to be able to tell what they were"), and

(5) an unpleasant *hedonic valence* ("It was not a pleasant experience", "painful").

This clarifies the picture of the description. It is also useful to distinguish between the difficult character of this experience, and the changes to the sense of self-world boundaries, because other reports describe similar changes to self-world boundaries as very pleasant. Other "symptoms" (such as the possible tiredness or confusion and hypersensitivity) may help distinguish between this and other types of existentially impactful experience.

In this way, functionally, what we are doing here is compiling an inventory of signs and symptoms that can inform a potential differential diagnosis and thus opens the possibility of lending clinical value.

Broad, gestalt-like phenomena and experiences can then be “reconstructed” as clusters of salient, functionally relevant, dimensional phenomenal features, much in the way that clinicians approach their patients’ experience. This approach can also be used to describe the diachrony of specific experiences of different scales by introducing a time component — how various dimensions evolved over *e.g.* the duration of a practice session, or over a month-long retreat.

Inventory

During practice, or while psychoactive compound(s) or other precipitating factors are still active

Aesthetic[6,7] or Numinous[8]

Finding beauty in a previously difficult experience (in this case, re-experiencing a past trauma "with a sense of the beauty of it") (Buddhist meditation NOS, [e])

Intense sense of **Gracefulness** (N,N-DMT, [11])

Mild to extreme experiences of beauty or the sublime, either specific (such as perceiving "beautiful colors" [N,N-DMT, [11]), or not (Jhāna meditation, [t]; N,N-DMT, [11]; Ibogaine, [6]).

> **Sense of Beauty** (Jhāna meditation, [t]; Ibogaine, [6]; N,N-DMT, [11])
> Light (Ibogaine, [6])
> Extreme/intense (N,N-DMT, [11])
> **Experience of the Sublime**, extreme (N,N-DMT, [11])

Intense sense of **Purity** (N,N-DMT, [11])

Transfigured perception of "the world" or "life", *e.g.* Perceiving "life" as "a golden world" (Spontaneous NOS, [δ]) or **Perceiving the visual world as a "Magical landscape"** infused with "the experience of location and non-location" (Meditation NOS, [b])

---

6 The adjectives serving as category headings implicitly denote kinds of emergent experiences, phenomena or effects (EPEEs) — *i.e.*, aesthetic *EPEEs*, archetypal *EPEEs*, and so on.
7 Having to do with a sense of beauty, of awe, of the sublime, etc.
8 A term used by Rudolf Otto and later by the likes of C.G. Jung to refer to feelings of "sacredness", "divinity" or "mystery" — be it *augustum* (majestic) or *tremendum* (awe-inspiring).

Archetypal, Visionary, Mythical or Symbolic[9]

Experiences involving **Nature archetypes[10]** (Concentration on visual object, [d]; Anthroposophic Meditation, [u]; NDE, [γ])

Feeling like **One became nature** (Concentration on visual object, [d]) or **became one with nature** or the periphery or specific objects (such as **rocks** or **minerals**) (Anthroposophic Meditation, [u]), sense of **Unity with nature** (NDE, [γ])
Plants (Anthroposophic Meditation, [u])
Sense that "**Nature is breathing**" or that one is able to feel Nature breathe (Concentration on visual object, [d])
Sensing nature as a whole (Anthroposophic Meditation, [u])

Experiencing an **Overwhelming sense of holiness and feeling one has tapped into "universal mind"** (Meditation (various), [l])

Feeling like one is in a state of **"Union with God"** (NOS Christian practice, [w]), sensing "God within oneself" (NDE, [γ])

Sense of **Connection "with a teacher who was not physically present"** (Meditation NOS, [v])

Sense of **Encountering "seemingly autonomous entities," "nonphysical entities," "physical beings" or "presences"** (Meditation NOS, [v]; Anthroposophic Meditation, [u]; Ibogaine, [6]; N,N-DMT, [11]; Salvia divinorum, inhaled, [15]; Coma/Accident NOS, [α]; NDE, [γ]; Spontaneous NOS, [δ]). These extremely varied interactions are described as ranging from extremely positive, to neutral, to extremely negative.

Reported Types of presences

Aliens, celestial beings, and extra-terrestrials (N,N-DMT, [11]; Spontaneous NOS, [δ])

Alien encounter (Spontaneous NOS, [δ]; N,N-DMT, [11]) or **Celestial beings** NOS (N,N-DMT, [11])
Beings made of light or energy (N,N-DMT, [11]; NDE, [γ])
Jeweled beings (N,N-DMT, [11])
Geometric, fractal, or hyperdimensional entities (N,N-DMT, [11])
Grey aliens (N,N-DMT, [11])

---

[9] Experiences of and alterations in relationship to "archetypes", "symbols", "images" and "myths" in the psychological, Jungian sense. Our criteria for differentiating between novel visual sensations (e.g. simple visual impressions), novel perceptual content of a visual nature (e.g. perceiving some specific visual thing), and visionary experiences (e.g. being immersed in a seemingly real alternate reality), is the degree of elaboration and immersiveness of the experience. See also Horváth, L., Szummer, C., & Szabo, A. (2018). Weak phantasy and visionary phantasy: the phenomenological significance of altered states of consciousness. *Phenomenology and the Cognitive Sciences*, *17*(1), 117–129. https://doi.org/10.1007/s11097-016-9497-4.
[10] Not "natural entities" or "beings" as below.

Angels (Meditation NOS, [v]; Anthroposophic Meditation, [u]; N,N-DMT, [11]; Spontaneous NOS, [δ]), **Archangels** (Anthroposophic Meditation, [u]) or **Seraphim** (N,N-DMT, [11])

Angelic phenomenology:

Light (Anthroposophic Meditation, [u])

Goodness (Anthroposophic Meditation, [u])

Localized sensed presence (Anthroposophic Meditation, [u])

Animals, Animal-based and creature-based entities (N,N-DMT, [11]; Spontaneous NOS, [δ])

Insectoid, praying mantis, or arachnoid entities (N,N-DMT, [11])

Feline entities (N,N-DMT, [11])

Octopus-like, squid-like, and jellyfish-like entities (N,N-DMT, [11])

Reptilian or serpentine entities (N,N-DMT, [11])

Seal — E.g., perceiving a seal "talking in the water" (Spontaneous NOS, [δ])

Tentacled entities (N,N-DMT, [11])

Archetypal feminine entity (N,N-DMT, [11]; Ibogaine, [6]) — *e.g.*:

Goddess or feminine deity (N,N-DMT, [11])

Gaia (N,N-DMT, [11])

Mother Ayahuasca (N,N-DMT, [11])

Mother Nature (N,N-DMT, [11])

Female entity or feminine presence NOS (N,N-DMT, [11]; Ibogaine, [6])

A "girl" (Ibogaine, [6])

I remember seeing a girl, like a girl's hand holding my hand. (Ibogaine, [6])

A Doppelgänger or shadow (Anthroposophic Meditation, [u]), a "**dark spirit**" like a "black fog" (Ibogaine, [6])

It's a dark spirit ... almost like fog, but like black fog ... it was dark, but it wasn't like it wanted to hurt you, it was no bullshit, it wasn't here to play games it was here to do what it was supposed to do ... (Ibogaine, [6])

Ghosts or Poltergeists (Spontaneous NOS, [δ])

Gods (Ibogaine, [6]), a God presence (Meditation NOS, [v]), **Divine beings** (Meditation NOS, [v]), **Deities, divine beings, demi-gods** (N,N-DMT, [11]) — *e.g.*:

Ancient Egyptian deities and entities (N,N-DMT, [11])

Buddhist deities (including **Buddha**) (N,N-DMT, [11])

God (N,N-DMT, [11])

Gods

"I saw things that I can’t even explain. Lots of lights, and all kinds of Gods" (Ibogaine, [6])

Hindu deities (N,N-DMT, [11])

Jesus Christ (N,N-DMT, [11]; Anthroposophic Meditation, [u])

Mary Magdalene (N,N-DMT, [11])

Guides (Meditation NOS, [v]; Ibogaine, [6])

An "indian man" guiding one (Ibogaine, [6])

Higher powers (Meditation NOS, [v])

A Jester, joker, or clown (Salvia divinorum, inhaled, [15]; N,N-DMT, [11])

“There were magical beings in that world, wearing garish dresses, similar to the clothes of a royal court jester” (Salvia divinorum, inhaled, [15])

Threatening presence, demons or negative figures (Anthroposophic Meditation, [u]; Meditation NOS, [v]; N,N-DMT, [11])

The **Devil** (N,N-DMT, [11])

Satan (N,N-DMT, [11])

Demons (N,N-DMT, [11])

Death-type entities (N,N-DMT, [11])

Various Mythological beings (Salvia divinorum, inhaled, [15]; N,N-DMT, [11]; Ibogaine, [6])

Magical Beings (Salvia divinorum, inhaled, [15])

Machine elves (N,N-DMT, [11])

Elves NOS (N,N-DMT, [11])

Faerie, sprites, nymph, spirits, or wisps (N,N-DMT, [11])

Giants, trolls, gnomes, imps, or goblins (N,N-DMT, [11])

Therianthropes, chimeras, and hybrids (N,N-DMT, [11])

Dragons (N,N-DMT, [11]), A "big dragon" (Ibogaine, [6])

Witches, wizards, or a **bearded individual** (N,N-DMT, [11])

Visitors (Meditation NOS, [v])

Other entity descriptors (Anthroposophic Meditation, [u]; Salvia divinorum, inhaled, [15]; N,N-DMT, [11]; Ibogaine, [6]; Coma/Accident NOS, [α]; NDE, [γ]; Spontaneous NOS, [δ])

“**Beings of nature**” (Anthroposophic Meditation, [u])

Children (N,N-DMT, [11]; Spontaneous NOS, [δ])

Apparition of a schoolboy (Spontaneous NOS, [δ])

Apparition of a stillborn child (Spontaneous NOS, [δ])

Conductor or entity with a top-hat or tuxedo (N,N-DMT, [11])

Culturally specific entities
Asian or Thai entities (N,N-DMT, [11])
Greek, Germanic, or Norse entities (N,N-DMT, [11])
Indigenous Meso-/South American entities (N,N-DMT, [11])
"The **Guardian of the threshold**" (Anthroposophic Meditation, [u]), a **Gatekeeper**, golem, or guard (N,N-DMT, [11])
Humanoid (N,N-DMT, [11])
A **Living acquaintance** (who wasn't present) (NDE, [γ])
A **Mystical being**, a close presence (Coma/Accident NOS, [α])
Other life forms (Salvia divinorum, inhaled, [15])
Robot or **machine entity** (N,N-DMT, [11])
Religious figure or a **Monk** (Coma/Accident NOS, [α]; NDE, [γ]; Spontaneous NOS, [δ])
Shadowy, silhouette, dark, or black entities (N,N-DMT, [11])
Shaman, **guide**, **nurse**, or **caretaker** (N,N-DMT, [11])
Faceless (N,N-DMT, [11])
Blue, white, red, green, or golden entity (N,N-DMT, [11])
Face-only or masked entity (N,N-DMT, [11]; Ibogaine, [6])
Tall slender entity (N,N-DMT, [11])
An **Unidentified stranger** (NDE, [γ])
Other descriptors
Self-aware (N,N-DMT, [11])
Intelligent (N,N-DMT, [11])
Benevolent (N,N-DMT, [11])
Petitionable (N,N-DMT, [11])
All-knowing (N,N-DMT, [11])
Sacred (N,N-DMT, [11])
Possessing Agency (N,N-DMT, [11])
Eternal (N,N-DMT, [11])

Sense that one **Communicated/interacted with an entity**[11] (N,N-DMT, [11]; Ibogaine, [6]; Salvia divinorum, inhaled, [15])
Types of interactions
Positive interactions (N,N-DMT, [11]; Ibogaine, [6])
"One of the faces came over and was like, 'everything's fine, just lay down, you're gonna be okay.'" (Ibogaine, [6])
benevolent (i.e., kind, compassionate, altruistic, etc.) interaction (N,N-DMT, [11])
comforting, protecting, or outwardly caring interaction

[11] Here we make a distinction between this less elaborated perceptual phenomena such as simply "hearing a voice"; however, clearly, an immersive experience of interacting and communicating with another "being" — whatever the ontological status we assigned to that — involves perceptual and sensate elements. The demarcation isn't binary but rather on a continuum.

welcoming (N,N-DMT, [11])
loving or embracing (N,N-DMT, [11])
dancing, singing, or partying (N,N-DMT, [11])
Healing (N,N-DMT, [11])
sexual, intimate, or erotic interaction (N,N-DMT, [11])
happy, friendly, or excited interaction (N,N-DMT, [11])
being provided nourishment (N,N-DMT, [11])
affusion-type or aspersion-type action or the entity pouring liquid on the subject (N,N-DMT, [11])
Positively judgmental entity interaction (N,N-DMT, [11])

Neutral interaction (Ibogaine, [6])
"It’s a dark spirit ... almost like fog, but like black fog ... it was dark, but it wasn’t like it wanted to hurt you, it was no bullshit, it wasn’t here to play games it was here to do what it was supposed to do ..." (Ibogaine, [6])
entity was playful or mischievous (N,N-DMT, [11])
entity touched or interacted with the subject's forehead or chest (N,N-DMT, [11])

Negative or difficult entity interaction (N,N-DMT, [11]; Ibogaine, [6])
"I remember specifically seeing a blackish face, it had horns on it, it had this tongue out, and it was bright red, but its eyes were just staring at me ... it scared the shit out of me ..." (Ibogaine, [6])
Negatively judgmental interaction (N,N-DMT, [11])
menacing, maliciousness, evil, threatening, violent, attacking, intimidating, or bullying entity (N,N-DMT, [11])
angry, frustrated, unfriendly, unhappy, disappointed, or sad entity (N,N-DMT, [11])
rejecting, denying, or unwelcoming encounter (N,N-DMT, [11])
being tortured or raped by an entity (N,N-DMT, [11])
being torn apart or eaten/consumed by an entity (N,N-DMT, [11])
Perceiving a presence/being as **forces that pulled one's body or talked to one**, creating auditory "hallucinations" (Salvia divinorum, inhaled, [15])

Companion-type, pedagogical, or guide-type interaction (N,N-DMT, [11])
entity guiding, touring, or showing (N,N-DMT, [11])
entity beckoning or summoning (N,N-DMT, [11])
entity tested or offered a choice (including option to live or die) (N,N-DMT, [11])
entity controlled or altered the visuals of the experience (N,N-DMT, [11])

Medical-type interaction (N,N-DMT, [11])
entity examining, observing, scanning, or analyzing the subject (N,N-DMT, [11])
implantation of a device (N,N-DMT, [11])
surgery, procedure, operation, injection, or experimentation (N,N-DMT, [11])
bodily exploration or probing (N,N-DMT, [11])

Open or closed-eyes, complex, multimodal and immersive **Visions or Scenarios** (Meditation NOS, [v]; Christian Contemplative Prayer, [w]; Ibogaine, [6]; N,N-DMT, [11]; Salvia divinorum, inhaled, [15]; Spontaneous NOS, [δ]). These can range from feeling like one is " fighting the devil" (Ibogaine, [6]); experiencing "many visions" (*ibid.*); psychologically liberating, seeming interactions with deceased relatives (Ingested Ayahuasca, [9]); and a tremendous (in fact, infinite) variety of unusual perceptual contents, such as:

Reported Contents
Feeling like one is fighting the devil (Ibogaine, [6])
I fought the devil… he was telling me to give up and die, but I didn't want to and I somehow beat him. And that I thought was my addiction at the time (Ibogaine, [6])
Experience of being led to a campfire (Ibogaine, [6]) or that "one was guided by an indian man" (Ibogaine, [6])
I was in the woods, and there was this Indian man, who had led me to a campfire, and there was a bunch of other Indian men around and they were all doing their hopping dance that Indians do around the fire (Ibogaine, [6])
Perceiving a seal "talking in the water" who asks 'are you ready to go along this path?' (Spontaneous NOS, [δ])
Landscape and landscape features (N,N-DMT, [11])
alien, fractal, or golden landscape (N,N-DMT, [11])
urban landscape (N,N-DMT, [11])
Fields (N,N-DMT, [11])
a jungle, forest, or ancient forest (N,N-DMT, [11])
beach, ocean, lake, or island (N,N-DMT, [11])
Astronomical themes (N,N-DMT, [11])
stars, (N,N-DMT, [11])
planets, (N,N-DMT, [11])
celestial bodies, (N,N-DMT, [11])
galaxies, and (N,N-DMT, [11])
outer space NOS (N,N-DMT, [11])
Machinery, clockwork, gears, or wheels (N,N-DMT, [11])
Ancient and/or cultural-specific architectural themes (N,N-DMT, [11])
ancient Egyptian typology (N,N-DMT, [11])

indigenous Meso-/South American (i.e., Aztecan, Mayan, etc.) (N,N-DMT, [11])
ancient Greco-Roman or Norse (N,N-DMT, [11])
Carnival imagery (N,N-DMT, [11]; Salvia divinorum, inhaled, [15]), circus or playhouse (N,N-DMT, [11])
"There were magical beings in that world, wearing garish dresses, similar to the clothes of a royal court jester" (Salvia divinorum, inhaled, [15])
Vehicle (N,N-DMT, [11])
Spaceship (N,N-DMT, [11])
Pyramids (N,N-DMT, [11])
Place of worship (N,N-DMT, [11])
Spherical objects (N,N-DMT, [11])
Globes and orbs (N,N-DMT, [11])
Cubes or containers (N,N-DMT, [11])
Helices, deoxyribonucleic acid (DNA), spirals, or hourglass-shapes (N,N-DMT, [11])

Perceiving a novel or alien language (N,N-DMT, [11])

Seeing one's life acted in front of oneself, life review (Ingested Ayahuasca, [9]; Coma/Accident NOS, [α]), sense of **Being judged or held accountable for one's life** (NDE, [γ])

Sense of **Encountering and/or Interacting with a deceased relative or other spirit**[12] (N,N-DMT, [11]; Ibogaine, [6]; Ingested Ayahuasca, [9]; Coma/Accident NOS, [α]; NDE, [γ])
"I felt my grandma, I just felt her presence everywhere and I realized that she was all around the whole time" (Ibogaine, [6])
Psychologically liberating, seeming interactions with deceased relatives (Ingested Ayahuasca, [9])
Sense of having visions of relatives (dead or alive) (N,N-DMT, [11]; Ibogaine, [6])
Sense of encountering and communicating with deceased spirits (Coma/Accident NOS, [α]; NDE, [γ])

Sense of **Entering, disappearing, being taken to, breaking through or falling into another world** (Ibogaine, [6]; Esketamine, [5]; N,N-DMT, [11]; Salvia divinorum, inhaled, [15]; Ingested Ayahuasca, [9]; Coma/Accident NOS, [α]; NDE, [γ]; Spontaneous NOS, [δ]; Epileptic Seizure, [β])
Feeling as if one is **Falling into darkness** (Epileptic Seizure, [β])
Experiencing **other worlds of multiple colors** (Salvia divinorum, inhaled, [15])
"I was in an immense forest full of fluorescent trees"
Sense of being on a different planet, e.g. "I felt like I was on a different planet..." (Salvia divinorum, [18]), of **being in two realities at the same time** (Salvia divinorum,

[12] This is distinguished from the previous effect as suggested by Yaden and Newberg (2022).

inhaled, [15]) or of **traveling to other dimensions** (Salvia divinorum, inhaled, [15]), *e.g.* "I have left my body and traveled to another world or dimension"

Sense that a "presence" is "trying to take the left side of the body too" (Salvia divinorum, inhaled, [15])

Feeling like one is in **hell** or **heaven** or **nirvana** (Ibogaine, [6]; N,N-DMT, [11])

Feeling like one is in outer space (Ibogaine, [6])

Feeling like one is flying through canyons and mountains (Ibogaine, [6])

Feeling like one is in the woods (Ibogaine, [6])

Feeling like one is sent back to when they were first born (Ibogaine, [6])

"It sent me back to when I was very first born" (Ibogaine, [6])

Feeling like one is inside the womb (Ibogaine, [6])

"I felt like I was inside the womb" (Ibogaine, [6])

Feeling like one is floating up the atmosphere (Ibogaine, [6])

I was able to float up in the atmosphere (Ibogaine, [6])

Feeling that one is standing in a big grassy field with blue sky (Ibogaine, [6])

Feeling that one is going down or up an underground tunnel

Sense of being inside a room (N,N-DMT, [11])

being in the "waiting room" (N,N-DMT, [11])

Reports of a sterile, clean, or functional room (including a medical, operating, or examination rooms) (N,N-DMT, [11])

geometric, fractal, or multi-coloured room (N,N-DMT, [11])

white or bright room (N,N-DMT, [11])

Sense of being inside a domed or large sphere (N,N-DMT, [11])

Sense of being in a different place than where physically present (Ingested Ayahuasca, [9])

Sense of "spiraling through a colorful tunnel" (Ingested Ayahuasca, [9])

Sense of drifting through a dark environment that looks like a tunnel (Coma/Accident NOS, [α])

Sense of discovering another world described as idyllic (Coma/Accident NOS, [α])

Sense of entering some other unearthly world or environment (Coma/Accident NOS, [α]; NDE, [γ])

Sense of coming to a border or a point of no return (Coma/Accident NOS, [α]; NDE, [γ])

Sense of breaking through some sort of veil (Spontaneous NOS, [δ])

Sense of passing through a tunnel (NDE, [γ])

Sense of **Perceiving "the light of wisdom" or "the light of eternal life"** (NOS Buddhist practice, [w])

Sensing **Something greater than oneself** (NOS Christian practice, [w])

Sense of having **Visions of "previous lives"** (Meditation NOS, [k]; N,N-DMT, [11])

Arousal-related[13]

High Arousal

High arousal with negative effect

Agitation (Meditation NOS, [g])

Difficulty sleeping (Ibogaine, [6]; Classical Psychedelics in lifetime users, [19])

Restlessness (Meditation NOS, [g])

High arousal with positive affect (MBSR/Buddhist meditation NOS, [c]; Jhāna meditation, [t]; Meditation NOS, [g]; 2C-B, Ingested, [14]; Salvia divinorum, [18]; MDMA, ingested, [22]; Spontaneous NOS, [δ]; Accident/Emergency NOS, [ε])

Feeling **less "drowsy"** (Salvia divinorum, [18])

High energy (Jhāna meditation, [t]), Massive "spiritual energy" (Spontaneous NOS, [δ])

Increased arousal, wakefulness or alertness

Intense arousal (Meditation NOS, [g])

Greater wakefulness (MBSR/Buddhist meditation NOS, [c])

Heightened alertness (Accident/Emergency NOS, [ε])

Markedly increased arousal (MDMA, ingested, [22])

Significant increase in vigor (2C-B, Ingested, [14])

Significant increase in elation (2C-B, Ingested, [14])

Low Arousal

Exhaustion

Feeling exhausted (Epileptic Seizure, [β])

Experiences on the border of sleep and awake

Sleep-like, shallow **Torpor** (MBSR/Buddhist meditation NOS, [c])

Fatigue or **Tiredness** (Meditation NOS, [g]; Buddhist meditation NOS, [q]; late MDMA, [4]; Psilocybin, ingested, [14]; Epileptic Seizure, [β])

Feeling "whacked out" (Buddhist meditation NOS, [q])

Sleepiness (Meditation NOS, [g]; Epileptic Seizure, [β])

[13] Alterations in the quality, mode, and level of arousal itself, including experiences on the borders of sleep and waking (e.g. hypnagogic experiences). What may happen in various forms of sleep, including in dreams and deep sleep, such as lucid dreams and lucid deep sleep, as well as some of the parasomnias, is included under the "wakefulness" heading.

Behavioral[14]

Chanting aloud (Ingested Ayahuasca, [9])

Crying — *e.g.* out of sadness (Buddhist meditation NOS, [p]) or out of happiness or joy (Meditation NOS, [k]; Ingested Ayahuasca, [9]; N,N-DMT, [11])

Laughing (Buddhist meditation NOS, [p]; Salvia divinorum, inhaled, [15]), **Uncontrollable laughter** (Salvia divinorum, inhaled, [15])

Physical aggression or violence towards self or other (Psilocybin, [7]; LSD, [10])
    while blacked-out (LSD, [10])

Playfulness — Slightly increased sense of "playfulness" (MDMA, ingested, [22])

Prolonging practice for days (Concentration on visual object, [d])

Risky behavior
    Putting oneself or others at risk of physical harm (Psilocybin, [7])

Suicide attempt (Psilocybin, [7])

[14] Changes in personal behaviors.

Cognitive[15]

General

Becoming more centered (Anthroposophic Meditation, [u])
Confusion (Psilocybin, ingested, [14]; 2C-B, Ingested, [14]; *Salvia divinorum*, inhaled, [15]), sometimes extreme (N,N-DMT, [11]) and/or **Comprehension difficulty** (Ibogaine, [6]; Salvia divinorum, inhaled, [15]), *e.g.* "I remember thinking 'I can't wrap my head around what's happening'" (Ibogaine, [6]), not being able to understand what is happening (Salvia divinorum, inhaled, [15])
Delayed reaction times (Psilocybin, ingested, [14]; 2C-B, Ingested, [14])
Feeling "**Spacey**" (Epileptic Seizure, [β])
Feeling **Dazed** (Epileptic Seizure, [β])
Significant impairments in global cognitive function (Psilocybin, ingested, [14]; 2C-B, Ingested, [14])
Mental stillness (Buddhist meditation NOS, [p])
Very high levels of mindfulness (Theravadā śamatha and vipassanā Meditation, [h])

Novel Content

Development of **Trust in thinking** (Anthroposophic Meditation, [u])
Fulfilling sense of knowing (Transcendental meditation, [d]) (Anthroposophic Meditation, [u]) or **Heightened sense of knowing** (NOS, [w])
Insights (Anthroposophic Meditation, [u]; Jhāna meditation, [t])
- **Into "what processes underlie everyday cognition"** (Anthroposophic Meditation, [u])
- **Into how thoughts arise** (Anthroposophic Meditation, [u])
- **Into the nature of thoughts** (Anthroposophic Meditation, [u])
- Insights (Jhāna meditation, [t])

Loose associations (Meditation NOS, [g])
Trying to "figure everything out" (Meditation (various), [l])
Indecipherable thoughts (Epileptic Seizure, [β])
Single words repeated in one's thoughts (Epileptic Seizure, [β])

Structural modulations[16]

Attention

General

Decreased concentration (Psilocybin, ingested, [14]; 2C-B, Ingested, [14])

[15] Alterations in our processing abilities and the quality of cognition itself.
[16] Where appropriate, we differentiate between *structural modulations* and *novel content* - *eg*, "increased auditory sensitivity" is a structural modulation which is not content-specific, while "perceiving a hissing sound" is novel content. This reflects the distinction made, within the field of micro-phenomenology, between *structural* propositions and *content* propositions (Petitmengin *et al*., 2019). In some ways, this also reflects the traditional philosophical distinction between the *transcendental* in the technical, kantian sense (Kant, 2007), and the *empirical*.

Deep concentration (Theravadā śamatha and vipassanā Meditation, [h])

Distractedness, *e.g.* "..felt distracted by background sounds" (Salvia divinorum, [18])

Increased absorption — *e.g.* "I felt mesmerized by the pattern on the door" (Salvia divinorum, [18])

Increased or **Heightened Attention** (MBSR/Buddhist meditation NOS, [c]; Accident/Emergency NOS, [ε];) **Increased ability to direct and sustain attention on an object** (Anthroposophic Meditation, [u]) or to **observe one's thoughts, feelings and body sensations** (Vipassanā, [w]; Meditation NOS, [f])

Extreme attention to detail, sound and movement (Accident/Emergency NOS, [ε])

Heightened awareness (Accident/Emergency NOS, [ε])

Reduced ability to sustain and control attention to bodily sensations (Salvia divinorum, inhaled, [15]), **Decreased attention** (Meditation NOS, [g])

Sense of attentional momentum (Jhāna meditation, [t])

Disposition

Focused and dynamic (Self-boundary dissolution meditation techniques, [r])
Wide and dynamic (Self-boundary dissolution meditation techniques, [r])
Wide and static (Self-boundary dissolution meditation techniques, [r])
Formless (Self-boundary dissolution meditation techniques, [r])

Focus

Trouble focusing/Poor concentration (Buddhist meditation NOS, [e]; [i])
Distraction (Meditation NOS, [g])
Becoming more focused (Anthroposophic Meditation, [u])
Magnet-like focus or **gravity** (Jhāna meditation, [t])`

Shape

Panoramic, inclusive perspective (Buddhist meditation NOS, [i])

Ease

Effortless attention (Buddhist meditation NOS, [i]; Anthroposophic Meditation, [u]; Jhāna meditation, [t])

Saturation

Confusion at increased amount of sensory information coming in (post-retreat) (Buddhist meditation NOS, [q])

Clarity

Bright mind (Buddhist meditation NOS, [p])
Cognitive disorganization (Meditation NOS, [g])
Confusion (Meditation NOS, [g]; Epileptic Seizure, [β]), feeling of things not quite making sense (Meditation NOS, [k])

Intensity

Increased intensity of awareness (Meditation NOS, [v])

Memory

Amnesia (Meditation NOS, [g]; N,N-DMT, [11])
Partial amnesia (N,N-DMT, [11])
Full amnesia and "blackouts" (N,N-DMT, [11])
Diminished immediate and delayed spatial memory recall (Psilocybin, ingested, [14]; 2C-B, Ingested, [14])
Increased **False memories** (Meditation NOS, [g])
Memory issues (Buddhist meditation NOS, [e]; Meditation NOS, [g])

Meta-cognition/Meta-awareness[17]

Alterations in the sense of knowing, e.g. sound is heard but not 'known' (Meditation NOS, [b])

Delayed recognition of what is happening (Transcendental meditation, [d])

Loss of awareness to being under the influence of a drug (N,N-DMT, [11])

Increased or decreased **Meta-cognition** (Buddhist meditation NOS, [p]), **Awareness of awareness** (Meditation NOS, [v]) or **Meta-awareness** (Vipassanā, [w])

Meta-awareness easier to maintain, more balanced (Jhāna meditation, [t])

[17] Sparby (2020): "the ability to be aware that one is attending to something and what one is attending to at a given time and potentially act in a beneficial way"

Uncovering aspect of consciousness that one was previously unaware of, which can contain difficult psychological material (Anthroposophic Meditation, [u])

Thinking

Ability to stop the thought process at will (Anthroposophic Meditation, [u])

Ability to think clearly (Anthroposophic Meditation, [u])

Cessation of mental activity[18] (Transcendental meditation, [w]; Vedic Yoga, [w]) or **Absence of thought** (Transcendental meditation, [w])

Difficulty thinking (Classical Psychedelics in lifetime users, [19])Confusion (N,N-DMT, [11]; Ibogaine, [6])

Increased **Ease to think** (Anthroposophic Meditation, [u])

Increased **Intrusive thoughts** (interfering with ability to concentrate), e.g. "..lot of thoughts about my day..." (*Salvia divinorum*, [18])

Increased rapidity of thoughts (Coma/Accident NOS, [α]), **Extremely rapid and detailed thinking processes** (Accident/Emergency NOS, [ε])

Increased structure of thought (Anthroposophic Meditation, [u])

Less sticky thoughts (Jhāna meditation, [t])

Mental quiescence or **Silence** (Transcendental meditation, [w]; Vedic Yoga, [w])

Repetitive thoughts (Ibogaine, [6])

Thoughts or **Mind racing**[19] (N,N-DMT, [11]; Ibogaine, [6]; Meditation (various), [l]; Buddhist meditation NOS, [e]; Epileptic Seizure, [β])

---

[18] This may refer to different experiences, with distinct phenomenologies. In cases like "cessations of consciousness," where not only thinking but also sensations and all other conscious experiences have ceased, we classify this as an effect on "wakefulness."

[19] Distinguished from "Increased rapidity of thoughts" because there can be a qualitative difference between a chaotically fast and confused mind, and the speed and clarity of thinking which can sometimes arise, *e.g.* as in this case, in emergency situations.

Collective[20]

Sense of a “collective energy” (Meditation NOS, [v])

[20] Changes that occur when groups gather together in emergent contexts, doing emergent practices, or when having emergent phenomena.

Dimensional[21]

Sense of reduced dimensionality (Salvia divinorum, inhaled, [15])
"Consistent change of dimensionality, I pass from 3D to 2D."

Perception of alternate or higher dimensions (N,N-DMT, [11])
1- or 2-dimensions (N,N-DMT, [11])
4- or 5-dimensions (N,N-DMT, [11])
Hyperspace (N,N-DMT, [11])
alternate-, hyper-, or multi-dimensions NOS (N,N-DMT, [11])

Sense of interacting with entities from a different dimension/reality whilst not being present in that dimension/reality oneself (N,N-DMT, [11])

[21] Related to the sense of there being spaces and directions beyond ordinary 3+1 dimensional space-time, compression of dimensions to less than than 3+1 dimensions, as well as bending of them, creating other alterations of them, as well as other non-local effects.

Emotional[22]

The full range of emotions and emotional responses, from the extremes of pleasantness and refinement, to the extremes of unpleasantness and horror, is reported.

Positive

Acceptance (Ibogaine, [6])
    I felt [...] acceptance of things (Ibogaine, [6])

Acceptance of death or **removal of fear of death** (N,N-DMT, [11])

Amazement (Concentration on visual object, [d]; N,N-DMT, [11]; Ibogaine, [6]), sometimes extreme (N,N-DMT, [11]), feelings of **Astonishment** (Coma/Accident NOS, [α])

Amusement (N,N-DMT, [11]; Salvia divinorum, inhaled, [15]) — *Fun* (N,N-DMT, [11]), *Great amusement* (Salvia divinorum, inhaled, [15])

Awe (Jhāna meditation, [t]; N,N-DMT, [11])

Bliss (Jhāna meditation, [t]; Buddhist meditation NOS, [i]; N,N-DMT, [11]; Accident/Emergency NOS, [ε]), sometimes *Intense* (Buddhist meditation NOS, [i]), or *Extreme* (N,N-DMT, [11])

Belonging — Sense of belonging or being "home" (N,N-DMT, [11])

Comfort — **Feeling more comfortable** (*Salvia divinorum*, [18])

Connectedness — *e.g.* Amazing sense of **Connectedness** that feels like it spread beyond oneself (Spontaneous NOS, [δ])

Elation — **Markedly increased** (MDMA, ingested, [22])

Enjoyment (N,N-DMT, [11])

Euphoria (Buddhist meditation NOS, [e]; Christian Contemplative Prayer, [w]; N,N-DMT, [11) —
    Gentle (Buddhist meditation NOS, [e])
    Lasting for months (Buddhist meditation NOS, [e])
    Extreme (N,N-DMT, [11])

Familiarity — A sense of familiarity (with the experience) (N,N-DMT, [11])

[22] Elevations, depressions, and alterations of mood, often seemingly unrelated or only loosely related to current circumstances.

Gratitude — Extreme sense of **Gratitude** (N,N-DMT, [11])

Happiness (Jhāna meditation, [t]; NOS Buddhist practice, [w]; NOS Christian practice, [w]; Meditation NOS, [k]; N,N-DMT, [11]; 2C-B, Ingested, [14]), sometimes **Profound** (NOS Buddhist practice, [w]; NOS Christian practice, [w]) or **Extreme** (N,N-DMT, [11]), **Feeling significantly happier** (2C-B, Ingested, [14])

Joy (Anthroposophic Meditation, [u]; NOS Buddhist practice, [w]; NOS Christian practice, [w]; N,N-DMT, [11]; Coma/Accident NOS, [α])

Perceived as one's own (Anthroposophic Meditation, [u])
Perceived as that of another being (Anthroposophic Meditation, [u])
Extreme (N,N-DMT, [11])
Profound (NOS Buddhist practice, [w]; NOS Christian practice, [w])

Exhilaration or **Ecstasy** (Christian Contemplative Prayer, [w]; Transcendental meditation, [w]; Vedic Yoga, [w]; N,N-DMT, [11]) — *e.g.* **religious ecstasy** (Christian Contemplative Prayer, [w]), **Ecstacy** (Transcendental meditation, [w]), **Yogic ecstacy** (Vedic Yoga, [w]), **Extreme exhilaration**/ecstasy (N,N-DMT, [11])

Sense of **Intimacy** (Anthroposophic Meditation, [u])

Liberation or **Freedom —** Sense of Liberation or opening (N,N-DMT, [11])

Love (NOS Christian practice, [w]; N,N-DMT, [11]; MDMA, ingested, [22]; Spontaneous NOS, [δ]) — ***unconditional*** feelings of love (NOS Christian practice, [w]); ***intense*** feelings of love (N,N-DMT, [11]); feeling *much more loving* (MDMA, ingested, [22]); **pervasive** — *e.g.* "Every breath was a loving experience - it was like breathing in love" (Spontaneous NOS, [δ]) or "like a world-encompassing experience of love" (Spontaneous NOS, [δ])

Sense of receiving **Nourishment** (Anthroposophic Meditation, [u])

Peace, Tranquility and Calm (Anthroposophic Meditation, [u]; Buddhist meditation NOS, [i]; Transcendental meditation, [d]; Meditation NOS, [k]; Jhāna meditation, [t]; Esketamine, [5]; N,N-DMT, [11]; Salvia divinorum, [18]; Coma/Accident NOS, [α]; Accident/Emergency NOS, [ε])

Spontaneous (Anthroposophic Meditation, [u])
As a result of intentional cultivation (Anthroposophic Meditation, [u])
Deep peace leading to absence of desires and detachment (Anthroposophic Meditation, [u])
Feeling like everything is fine (Buddhist meditation NOS, [i])
Pervasive sense of calm (Transcendental meditation, [d])
Deep relaxed or calming states (Meditation NOS, [k])

Tranquility (Jhāna meditation, [t])
Extreme Peacefulness (N,N-DMT, [11])
Extreme calm (N,N-DMT, [11])
Feeling calmer (Salvia divinorum, [18])
Peace (Esketamine, [5])
Feeling imperturbable (Buddhist meditation NOS, [i])
Evenness (Buddhist meditation NOS, [i])
Overwhelming feelings of peacefulness (Coma/Accident NOS, [α]; Accident/Emergency NOS, [ε])
Calmness in the face of danger (Accident/Emergency NOS, [ε])
Lack of anxiety about danger (Accident/Emergency NOS, [ε])
Detachment (Accident/Emergency NOS, [ε])

Positive affect NOS (Buddhist meditation NOS, [p])

Pride (N,N-DMT, [11])

Reassurance — Extreme (N,N-DMT, [11])

Rejuvenation — Sense of **Being reborn** (N,N-DMT, [11])

Relaxation (Esketamine, [5])

Relief — Extreme (N,N-DMT, [11])

Surprise — e.g. by how calm one is in the face of danger (Accident/Emergency NOS, [ε]) or by how much detail one is able to take in (Accident/Emergency NOS, [ε])

Sense of **Warmth** (N,N-DMT, [11])

Wellbeing (N,N-DMT, [11]; Coma/Accident NOS, [α]; Accident/Emergency NOS, [ε]; Spontaneous NOS, [δ]) — Sense of healing or **Wellbeing** (N,N-DMT, [11])

Wonder (Anthroposophic Meditation, [u]; Jhāna meditation, [t]; N,N-DMT, [11])

Negative

Agitation or restlessness (Buddhist meditation NOS, [p]; [i]; Esketamine, [5])
Agitation (Buddhist meditation NOS, [p])
Restlessness (Buddhist meditation NOS, [i]; Esketamine, [5])

Alienation, Disconnection, Isolation or Loneliness (Meditation NOS, [g]; Classical Psychedelics in lifetime users, [19]; Salvia divinorum, inhaled, [15]; Esketamine, [5]; Ayahuasca, [3]; Psilocybin, [2]; Spontaneous NOS, [δ])

Feelings of isolation (due to not being able to feel the presence of the experimenter in the room) (Salvia divinorum, inhaled, [15])

Feeling disconnected or alone (Ayahuasca, [3])

Loneliness (Meditation NOS, [g])

Feeling distant or cut off from other people (Classical Psychedelics in lifetime users, [19])

Feeling isolated from everyone (Psilocybin, [2])

Feeling disconnected or isolated from everything (Classical Psychedelics in classic users, [19]; Psilocybin, [2])

Anger, aggression or rage (Buddhist meditation NOS, [p]; Meditation NOS, [g]; Psilocybin, ingested, [14]; Psilocybin, [2])

Significantly increased anger (Psilocybin, ingested, [14])

Experience of antagonism toward people around oneself (Psilocybin, [2])

Despair (N,N-DMT, [11]; Psilocybin, [2])

Extreme hopelessness (N,N-DMT, [11]; Psilocybin, [2])

Disbelief — Extreme disbelief (N,N-DMT, [11])

Disgust (Anthroposophic Meditation, [u])

Doubt (Buddhist meditation NOS, [i])

Fear, dread, worry, anxiety, panic, or terror (Meditation NOS, [g]; Anthroposophic Meditation, [u]; Meditation NOS, [v]; Concentration on visual object, [d]; Buddhist meditation NOS, [p], [i]; Meditation NOS, [k]; Hindu Meditation NOS, [m]; Classical Psychedelics in lifetime users, [19]; N,N-DMT, [11]; Ibogaine, [6]; Esketamine, [5]; MDMA, serotonergic psychedelics, [4]; Psilocybin, [2])

Anxiety or **anxiousness** (Meditation NOS, [g]; Hindu Meditation NOS, [m]; MDMA, serotonergic psychedelics, [4]; Esketamine, [5]; Psilocybin, [2]; Classical Psychedelics in lifetime users, [19]; Epileptic Seizure, [β])

Pananxiety (Hindu Meditation NOS, [m])

Intense anxieties (Hindu Meditation NOS, [m])

Feeling anxious (Epileptic Seizure, [β])

Feeling apprehensive (Epileptic Seizure, [β])

Fear (Concentration on visual object, [d]; Anthroposophic Meditation, [u]; Meditation NOS, [k]; Meditation NOS, [g]; Psilocybin, [2]; Ibogaine, [6]; Spontaneous NOS, [δ]; Epileptic Seizure, [β])

Strong fear reaction to alteration of experience which progressively dissipates as activity is continued (Concentration on visual object, [d])
Fear at sense of self disappearing (Anthroposophic Meditation, [u])
Great existential fear (Anthroposophic Meditation, [u])
Fear of losing one's mind or going insane (Psilocybin, [2])
Fear that the state one is in will last forever (Psilocybin, [2])
Salient fear of or disturbance by seemingly non-threatening phenomena
Disturbing "sounds of movement" and feeling of being very scared (Meditation NOS, [k])
Fear NOS (Meditation NOS, [g]), Some fear (Ibogaine, [6])
Fear of death (Spontaneous NOS, [δ])
Feeling scared (Epileptic Seizure, [β])

Panic (Meditation NOS, [g]; Psilocybin, [2])
Panic Attacks/Panic (Meditation NOS, [g])
Panic (Psilocybin, [2])

Terror (Meditation NOS; N,N-DMT, [11]; Ibogaine, [6])
Terror (Meditation NOS, [g])
Extreme terror (N,N-DMT, [11]
Feeling terrified (Ibogaine, [6])

Worry — Extreme worry (N,N-DMT, [11])

Grief (Psilocybin, [2])

Guilt — Feelings of Guilt (Hindu Meditation NOS, [m])

Irritability (Buddhist meditation NOS, [p]; [i]; Classical Psychedelics in lifetime users, [19])
Bothered by little things (Classical Psychedelics in lifetime users, [19])

Nervousness (Meditation NOS, [g])

Overwhelm (Esketamine, [5])

Paranoia (Buddhist meditation NOS, [p]; [i]; Psilocybin, [2]; Epileptic Seizure, [β])
Having the feeling that people are plotting against one (Psilocybin, [2])

Regret — Extreme Regret (N,N-DMT, [11])

Sadness, depression, dysphoria, low mood (Buddhist meditation NOS, [p]; Anthroposophic Meditation, [u]; Meditation NOS, [g]; Psilocybin, ingested, [14]; late MDMA, [4]; Psilocybin, [2]; Epileptic Seizure, [β])
Emotional suffering NOS (Psilocybin, [2])
Sadness (Psilocybin, [2]; Epileptic Seizure, [β])
Feeling like crying (Psilocybin, [2])

Low mood NOS (late MDMA, [4])
Increased feelings of depression (Psilocybin, ingested, [14])
Adverse emotional and mental states NOS (Meditation NOS, [g])

Self-conscious emotions (Buddhist meditation NOS, [p])

Shock — Extreme Shock (N,N-DMT, [11])

Significantly increased levels across all negative moods (Psilocybin, ingested, [14])

Stress (Esketamine, [5]; Spontaneous NOS, [δ]) — sometimes **Extreme** (because of sensory overload) (Spontaneous NOS, [δ])

Suicidality (Buddhist meditation NOS, [p]; MDMA for PTSD [16]; N,N-DMT, [11])
Increased feelings of suicidality (MDMA for PTSD, [16])

Tension (Esketamine, [5]; Psilocybin, ingested, [14]; 2C-B, Ingested, [14])
Significantly increased tension (Psilocybin, ingested, [14]; 2C-B, Ingested, [14])

Feeling **Threatened** or Pursued (Epileptic Seizure, [β])

Feeling **Trapped**, or claustrophobia (N,N-DMT, [11])

Unease NOS (Esketamine, [5])

Neutral

Affective flattening or emotional detachment (Buddhist meditation NOS, [p])

Subdued emotions
Didn't feel particularly wonderful (Transcendental meditation, [d])

Other

Emotional disconnection (Meditation NOS, [g])

Paradoxical emotions (Salvia divinorum, inhaled, [15]; Spontaneous NOS, [δ]) — *e.g.* a combination of being 'scared out of one's wits' with lots of excitement (Spontaneous NOS, [δ])

Mood swings, or **Emotional lability** (Psilocybin, ingested, [14]; Meditation NOS, [g]; Buddhist meditation NOS, [p]; Mindfulness, [a]; Meditation (various), [l]; Hindu Meditation NOS, [m])

Energetic[23]

Sensing **Energy and forces outside the body** (Anthroposophic Meditation, [u])

Sense of **Energy exchange between body and environment** (Anthroposophic Meditation, [u])

Sensing **Energetic sensations, Forces or Power in the body** (Anthroposophic Meditation, [u]; Buddhist meditation NOS, [e]; Jhāna meditation, [t]; Buddhist meditation NOS, [i]; Meditation NOS, [k]; Ingested Ayahuasca, [9]; Spontaneous NOS, [δ]; Epileptic Seizure, [β]) — sometimes **Intense** (Buddhist meditation NOS, [i]), **Massive** and/or **Overwhelming** (Spontaneous NOS, [δ]), sometimes **Controllable** (Anthroposophic Meditation, [u])

- Energy-types
  - **Autonomous** — *i.e.* sense that somatic energy possesses its own agency and volition and is performing intentional actions on the body (Buddhist meditation NOS, [e])

  - **Blockages** (Buddhist meditation NOS, [e]) or **Pressure** (Buddhist meditation NOS, [e])
    - **Build-up** (Buddhist meditation NOS, [e])
    - **Constriction** (Buddhist meditation NOS, [e])
      - In the chest (Buddhist meditation NOS, [e])
    - **Knots** (Buddhist meditation NOS, [e])
    - **Interrupted flow** (Buddhist meditation NOS, [e])
    - Sense of **Stuck energy** (Buddhist meditation NOS, [e])
      - Around the heart (Buddhist meditation NOS, [e])
      - Concentrating in the head (Buddhist meditation NOS, [e])
    - Feelings of **Pressure** (Buddhist meditation NOS, [e])
      - In the head (Buddhist meditation NOS, [e])
    - **Squeezing** (Buddhist meditation NOS, [e])

  - **Burning** or **Heat sensations** (Buddhist meditation NOS, [e]; Spontaneous NOS, [δ])
    - Fire (Buddhist meditation NOS, [e])
    - Exploding (Buddhist meditation NOS, [e])
    - Sense of fire coming out of the top of the head (Buddhist meditation NOS, [e])

---

[23] Effects for which the word "energetic", or "energy-like", is probably the best word we have, even if we don't yet entirely know what that is physiologically, and that involves experiences that may involve a sense of vibrations, power, movements (see motor/kinetic), colors, sounds, and moods, changes in wakefulness and perception, often related to various "centers" in the body, and described by such terms as "kundalini", "rapture", etc. In some ways, this is a broader category than others. Energetic phenomena are often described — *e.g.* in psychedelic publications — as kinds of spontaneous tactile sensations, and when they were appraised as such, we included them under the heading "sensate".

"I felt like I was glowing - as if I was generating heat" (Spontaneous NOS, [δ])

Sensing energetic "**Channels"** (Buddhist meditation NOS, [e]),

Currents (Buddhist meditation NOS, [e]), **Flow** (Buddhist meditation NOS, [e]), **Streams** (Anthroposophic Meditation, [u]), **Vortex** (Buddhist meditation NOS, [e]), or **Waves** (Buddhist meditation NOS, [e]; Jhāna meditation, [t]; Epileptic Seizure, [β]) — *e.g.*:

Feeling waves of energy pulsing through the body (Epileptic Seizure, [β])
Constant sense of energy flowing through the body (Buddhist meditation NOS, [e])
Extremely painful energy flows (Buddhist meditation NOS, [e])
Tingling (Anthroposophic Meditation, [u])
Rotation (Anthroposophic Meditation, [u])
Centering (Anthroposophic Meditation, [u])
Expanding force (Anthroposophic Meditation, [u])

Electrical (Buddhist meditation NOS, [e])

Bolts (Buddhist meditation NOS, [e])
Jolts (Buddhist meditation NOS, [e]; Epileptic Seizure, [β]) or **Electric-shock-like sensations** (Meditation NOS, [k])
Currents (Buddhist meditation NOS, [e])
Voltage (Buddhist meditation NOS, [e])
Feeling wired (Buddhist meditation NOS, [e])
Feeling like your circuits are gonna get blown out (Buddhist meditation NOS, [e])
Charge-like energetic sensations (Meditation NOS, [k])

Energy-Light (Buddhist meditation NOS, [e]; Spontaneous NOS, [δ])

White energy light (Buddhist meditation NOS, [e])
"I felt like I was glowing" (Spontaneous NOS, [δ])

Fizzing (Buddhist meditation NOS, [e]), **Effervescence** (Buddhist meditation NOS, [e]) or **Vibrations** (Buddhist meditation NOS, [e]; Ingested Ayahuasca, [9])

Vibration (Anthroposophic Meditation, [u])
Vibration cascading through the body (Buddhist meditation NOS, [e])
Intensifying drum-like vibration in one's heart, progressively including the whole body (Ingested Ayahuasca, [9])

Mechanistic (Buddhist meditation NOS, [e])

Feeling like a machine with gears (Buddhist meditation NOS, [e])
Grinding discomfort (Buddhist meditation NOS, [e])

Moving energetic sensations (Buddhist meditation NOS, [e])
Breaking through (Buddhist meditation NOS, [e])
"Crown chakra" (Buddhist meditation NOS, [e])
Energy **Building up the body and going down** over and over (Buddhist meditation NOS, [e])
Bursting (Buddhist meditation NOS, [e])
Vibration **Cascading** through the body (Buddhist meditation NOS, [e])
Hitting (Buddhist meditation NOS, [e])
Opening (Buddhist meditation NOS, [e])
Energy **Rising up** out of the pelvis and **going up** through the chest (Buddhist meditation NOS, [e])
Tearing (Buddhist meditation NOS, [e])
At muscles (Buddhist meditation NOS, [e])
Wiggling through the body (Buddhist meditation NOS, [e])

Rapture (physical) (Buddhist meditation NOS, [e])

Rush — *e.g.* Feeling a ‘sugar rush’ (Epileptic Seizure, [β])

Sense that one's body is being controlled by an energy (Buddhist meditation NOS, [e])

Existential[24]

Sense of self

Perceptions/Representations of Self

Sense of connection, identification, or union of the self with aspects or all of one's environment (Concentration on visual object, [d]; Anthroposophic Meditation, [u]; Buddhist meditation NOS, [q]; Accident/Emergency NOS, [ε])

Felt like one became Nature (Concentration on visual object, [d])
Experiences where the self was in some sense one with something external to it (Anthroposophic Meditation, [u])
Experiences of oneness with nature or the periphery or specific objects (such as rocks or minerals) (Anthroposophic Meditation, [u])
Sense of connection/oneness (Accident/Emergency NOS, [ε])
Feeling **Identified with everything** (Buddhist meditation NOS, [q])

Sense that one experienced "transcendence" or "enlightenment" (Anthroposophic Meditation, [u])

Feeling as if one was dead or dying (Psilocybin, [2]; Spontaneous NOS, [δ]), sense of **Profoundly experiencing one's own death** (Psilocybin, [2])

Increase in **Felt spatial extension of self** (Mindfulness meditation, [j]; Accident/Emergency NOS, [ε]; Buddhist meditation NOS, [q]) — *e.g.* Sense of being "everywhere, all at once" (Accident/Emergency NOS, [ε]), **"Extension" of sense of self beyond the body** to include objects or people in the environment (Buddhist meditation NOS, [q])

Sense of being **Split, Dissociated or Fragmented** (Transcendental meditation, [d]; Hindu Meditation NOS, [m]; Spontaneous NOS, [δ])

"Part of me was eating the meal and part of me was sitting inside laughing" (Transcendental meditation, [d])
Sense that one is observing one's own actions from a distance (Transcendental meditation, [d])
"[...] part of me was just sitting out back watching the whole thing take place" (Transcendental meditation, [d])
Acute "sensation" [sic] of being mentally split (Hindu Meditation NOS, [m])
Sense of self **splitting** (Spontaneous NOS, [δ]) or **fragmenting** (Spontaneous NOS, [δ])

Feeling less dissociated (Buddhist meditation NOS, [e])

---

[24] Alterations in our understanding of deep and layered aspects of existence and identity itself, including to our sense of agency, subjectivity, the centrality and coherence of identity, the continuity or ephemerality of experience, life before or after death, parallel lives, split existences, and the like.

Direct experience of the sense of self as impermanent (Buddhist meditation NOS, [i])

Sense of **Discovering more fundamental aspects of the self** (following spiritual crisis or not) (Anthroposophic Meditation, [u]) or **of encountering true self/identity** (NOS Buddhist practice, [w])

Dissolution of "ego" or "self" (Anthroposophic Meditation, [u]; Psilocybin, ingested, [14]; 2C-B, Ingested, [14])

- **Sense of "ego-dissolution"** (Psilocybin, ingested, [14]; 2C-B, Ingested, [14])
- **"Death-like" experience** - "a kind of death experience" (Anthroposophic Meditation, [u])
- **Sense that "Nothing is left" of former self** (Anthroposophic Meditation, [u])
- **Positive overall experience of "ego-dissolution"** (Anthroposophic Meditation, [u])
- **Negative overall experience of "ego-dissolution"** (Anthroposophic Meditation, [u])
    - **Sense of depersonalization** (Anthroposophic Meditation, [u])
    - **Scary sense of suddenly losing a sense of self** (Anthroposophic Meditation, [u])

Changes to felt **Location of self** — *e.g.* **Sense that the center of one's being, which was "normally in the head," moved to the heart** (Anthroposophic Meditation, [u]), **Experience of "location and non-location"** (Meditation NOS, [b])

Experiencing oneself as a child (Ibogaine, [6])

Radical changes to self perception (Salvia divinorum, inhaled, [15])

- Sense of **Becoming a geometrical object** (Salvia divinorum, inhaled, [15])
    - "I changed from being a square, to a pentagon, to a pyramid"

Sense of **perceiving the "radiance of our fundamental nature"** (NOS Buddhist practice, [w]) or **"experiencing pure consciousness"** (Transcendental meditation, [w])

Witnessing — *e.g.* feeling like a "massive self witnessing all" (Accident/Emergency NOS, [ε])

Agency

- **Sense that actions are doing themselves** (Transcendental meditation, [d])
    - "It feels just like the writing is flowing out as I am doing it" (Transcendental meditation, [d])
- **Sense of selflessness** (NOS Buddhist practice, [w])

Feeling that some other person, power, or force is performing actions through one's body, or **sense that one's body is being controlled** (Buddhist meditation NOS, [q]; [e])
Mild to significant decreased identification with thoughts, emotions and physical sensations (Buddhist meditation NOS, [i])
Loss of sense of agency (Buddhist meditation NOS, [p])
  Sense of there being no 'doer' or 'no one' who decides, controls, or executes actions (Buddhist meditation NOS, [q])
Loss of sense of "basic self" (Buddhist meditation NOS, [p])
  Feeling like one "isn't there" (Buddhist meditation NOS, [q])
Continuously active sense of agency (Self-boundary dissolution meditation techniques, [r])
Responsive/intermediate sense of agency (Task maintenance) (Self-boundary dissolution meditation techniques, [r])
Sense of passivity or non-doing (Self-boundary dissolution meditation techniques, [r])

Self-world boundaries

Changes in self-other or self-world boundaries (Buddhist meditation NOS, [p]; [q]; Mindfulness meditation, [j]; Anthroposophic Meditation, [u]), such as:
  Feeling "permeable" (Buddhist meditation NOS, [q])
  Decreased perceived boundary between self and world (Mindfulness meditation, [j])
  Sense of boundaries of the body dissolving or starting to flow (Anthroposophic Meditation, [u])
  Sense of **lack of separation** between one's body and people or objects in the environment (Buddhist meditation NOS, [q]), of **boundarylessness** (Buddhist meditation NOS, [q]), or **Boundlessness** (NOS Buddhist practice, [w])
  Feeling like others are inside oneself (Buddhist meditation NOS, [q])
  Loss of sense of boundaries (NOS Buddhist practice, [w])
General sense of spaciousness where **typical boundaries are not clear** i.e. **diffuse, spacious, open, not solid** ("like I'm made of air and airiness extends out") (Meditation NOS, [b])
Sense of internal versus external becomes vague i.e. sensory objects are no longer located outside (Meditation NOS, [b])
Things becoming much less distinguished and very existence becoming blurry, diffuse, uncertain (Meditation NOS, [b])
Experience of having "no center" (Meditation NOS, [b])
Normal sense of first-person perspective (Self-boundary dissolution meditation techniques, [r])
Intermediate sense of first-person perspective (Self-boundary dissolution meditation techniques, [r])
"Non-dual" state (Self-boundary dissolution meditation techniques, [r])
Sense of connection with the universe (Esketamine, [5])

Ownership

Sense of ownership weakens considerably (Meditation NOS, [b]), all the way to **Loss of sense of ownership** (Buddhist meditation NOS, [p])

Feeling that one's body, body parts, thoughts, or emotions no longer belong to one, sometimes to the extent that they are experienced as impersonal, as belonging to no one (Buddhist meditation NOS, [p]; Buddhist meditation NOS, [q]; Salvia divinorum, inhaled, [15])

Loss of sense of body ownership (Salvia divinorum, inhaled, [15])

Decrease in the need to decide as movement and agency becomes more and more spontaneous - things tend to happen by themselves (Meditation NOS, [b])

Losing the sense of having a personal point of view (Meditation NOS, [b])

Sense of reality

Feelings of "Derealization" (Buddhist meditation NOS, [p]; Mindfulness, [a]; Salvia divinorum, inhaled, [15]; Psilocybin, ingested, [14]; 2C-B, Ingested, [14]), **Feeling at times that either one is not real or that surroundings are not real** (Meditation (various), [l])

Becoming "dreamy", Dreamlike experience (Anthroposophic Meditation, [u]; Salvia divinorum, inhaled, [15])

Sense of disconnection with reality (Salvia divinorum, inhaled, [15])

Sense of being in two realities at the same time (Salvia divinorum, inhaled, [15])

“I really wanted to be fully in that other reality, it was very familiar, like the reality of my childhood"

Feelings of **Hyper- or Sur-reality** (N,N-DMT, [11])

Experience felt as or more real than everyday reality (N,N-DMT, [11])

Intense sense of the experience being hyper Real (N,N-DMT, [11])

Expressive[25]

Difficulty describing the experience[26] (Ibogaine, [6])

Increased use of positive emotion words (MDMA, ingested, [22])

[25] Changes in how we communicate and express ourselves. This and other categories (Semantic, Interpretive, etc.) could be construed as aspects of a larger "Language" theme that could include other dimensions beyond these.
[26] This is reminiscent of William James' famous "ineffability" mark of "mystical experiences." Interestingly, this was very rarely reported throughout the publications under review.

Functional[27]

Changes in executive functioning NOS (Buddhist meditation NOS, [p])

Increased functionality

Effortless practice (Buddhist meditation NOS, [i])

Sense of having more time to make decisions and take action during crisis (Accident/Emergency NOS, [ε])

Decreased functionality

Being concerned with 'who' will perform daily actions (Buddhist meditation NOS, [q])

Extreme difficulty talking (Ibogaine, [6])

Sense of **Helplessness** (Coma/Accident NOS, [α])

Impaired capacity to interact with body or surroundings (Salvia divinorum, inhaled, [15])

Physical prostration (Ibogaine, [6])

Laying in bed for an extended period during the trip (Ibogaine, [6])

Episodes of **Powerlessness** (Anthroposophic Meditation, [u])

[27] Changes to our activities of daily living and basic ability to function in the ordinary world.

Hedonic Valence[28]

Positive or negative valence (Anthroposophic Meditation, [u])

Unpleasant

Extreme discomfort or pain (N,N-DMT, [11])
Feeling "like crap" (Ibogaine, [6])
Unpleasant experiences (Buddhist meditation NOS, [q]; Esketamine, [5])
Slightly to highly negative experiences (Self-boundary dissolution meditation techniques, [r])
Distressing or hellish experiences (Coma/Accident NOS, [α])
Sense of helplessness (Coma/Accident NOS, [α])
Mildly negative (NDE, [γ])

Neutral

Neutral (Self-boundary dissolution meditation techniques, [r])

Anhedonia

Trouble enjoying things (Classical Psychedelics in lifetime users, [19])

Pleasant

Positive experiences (Self-boundary dissolution meditation techniques, [r]), Sense of extreme positivity (N,N-DMT, [11])
NOS Pleasurable effects (Salvia divinorum, inhaled, [15])
Pleasantness (Coma/Accident NOS, [α])
Very positive affect (NDE, [γ])
Intense pleasure — *e.g.*, like "thousands of orgasms going off in one's head" (Spontaneous NOS, [δ])

Mixed

Mixed (Various opposing emotions) (Self-boundary dissolution meditation techniques, [r])

[28] In the sense meant by psychology, meaning alterations in the degree to which an experience is perceived as pleasant/good, unpleasant/bad, or neutral, which may have a direct impact on our perceived quality of life.

Informational[29]

Sense of receiving non-verbal communication (N,N-DMT, [11]; Coma/Accident NOS, [α])

- Directionality of perceived entity communication (N,N-DMT, [11])
  - one-way from the entity to the subject (N,N-DMT, [11])
  - two-way (N,N-DMT, [11])
  - one-way from the subject to the entity (N,N-DMT, [11])
- Type of communication (N,N-DMT, [11])
  - Visual communication (N,N-DMT, [11])
  - Verbal-auditory communication (N,N-DMT, [11])
  - "Extrasensory" communication (N,N-DMT, [11])
  - Somatic communication (N,N-DMT, [11])
- Content
  - **Sense that a message, task, mission, or insight was given from the encounter** (N,N-DMT, [11])
  - **Sense of acquiring predictions about the future** (N,N-DMT, [11]; Coma/Accident NOS, [α])

---

[29] Related to a sense of receiving various forms of information, such as "downloads", channeling, precognition (which obviously contains a temporal component as well), and encompassing phenomena such as Third Man Factor.

Intuitive[30]

Insights that appear as specific ideas (Anthroposophic Meditation, [u])

Insights that are "like seeing something for the first time" (Anthroposophic Meditation, [u]) or **"like remembering something one has forgotten"** (Anthroposophic Meditation, [u])

Intuitive experiences

Sense of perceiving information that "could not have been known by usual means" (Meditation NOS, [v])

A sense of suddenly **Being able to understand everything** (Coma/Accident NOS, [α])

Wordless understanding (Anthroposophic Meditation, [u])

[30] Intuitions, insights, "aha moments," and so on. These differ from the previous label in that they are not accompanied by a sense of being communicated by another subject or person. See *e.g.* Claire Petitmengin's PhD dissertation, *L'expérience Intuitive [The Intuitive Experience]*, for a micro-phenomenological exploration of various kinds of intuitive experiences, from novel scientific insights to seemingly impossible intuitions of distant events, and a proposed generic structure.

Magical[31]

Hard-to-explain/seemingly "magical" occurrences (Meditation NOS, [v]; NDE, [γ])

Physical manifestations that seemed to have no physical cause (Meditation NOS, [v]), — *e.g.* Seeing objects move without apparent cause (Meditation NOS, [v]; NDE, [γ]); physical objects appearing when they had not been there before (Meditation NOS, [v]); something falling over (Meditation NOS, [v]); A light going out (Meditation NOS, [v])

Sense of experiencing **Increased synchronicities** (Meditation NOS, [v]), such as **Meaningful coincidences** (Meditation NOS, [v])

Unusual capacities

Sense of **Being aware of things going on elsewhere**, as if by ESP (NDE, [γ]; Coma/Accident NOS, [α])
Sense of **Being able to know another person's psychological experience** (Meditation NOS, [k]; NDE, [γ])
Sense of **Experiencing memories of a "previous life"** (NDE, [γ]) or seeing **"past lives"** (Meditation NOS, [k])
Sense of **Viewing "scenes from the future"** (Coma/Accident NOS, [α])
Sense of **Having an influence on electronic devices** (Buddhist meditation NOS, [e])

[31] A wide range of effects and experiences from which we might be tempted to infer something beyond "ordinary" materialistic causality and a single, shared Euclidean space, and about which we remain strictly ontologically agnostic while yet appreciating that reports of these sorts of effects and experiences may often have clinical relevance. This similarly is a more broad category than some of those above, and will need to be handled with nuance and care on multiple levels. Examples include descriptions of spoon bending, etc.

Medical[32]

Hospital referral (Hindu Meditation NOS, [m]), **Getting help at a hospital or emergency department** (Psilocybin, [7])

Hospitalization (Ayahuasca for depression, [17])

[32] Changes in our relationship to health, healing, the symptoms and trajectories of other medical conditions, including chronic pain or terminal conditions, and our overall sense of wellness or unwellness.

Meta-emergent[33]

Appreciation (of the experience) — *e.g.* Extreme (N,N-DMT, [11])

Strong sense of **Sacredness** of a specific experience (NOS Christian practice, [w])

Sense of **Miraculousness** of a specific experience (N,N-DMT, [11])

Feeling enlightened (Buddhist meditation NOS, [e]; N,N-DMT, [11]), Extreme sense of enlightenment (N,N-DMT, [11])

Sense of Magic of a specific experience (extreme) (N,N-DMT, [11])

Sense of **Surreality** of experience (N,N-DMT, [11])

Sense of the **Uniqueness of salvia compared to other psychedelics** (Salvia divinorum, inhaled, [15])

- “The experience was surprising, how weird it is!”, “This was a completely new experience for me.”
- Examples of retrospective evaluation of experience:
  - Most important experience of one's life (N,N-DMT, [11])
  - Most incredible, or extraordinary experience of one's life (N,N-DMT, [11])
  - Most complex, elaborate, or dazzling experience of one's life (N,N-DMT, [11])
  - Most spiritual experience of one's life (N,N-DMT, [11])
  - Most disturbing, debilitating, or appalling experience of one's life (N,N-DMT, [11])
  - Hardest, harrowing, or hectic experience of one's life (N,N-DMT, [11])
  - Most mesmerizing experience of one's life (N,N-DMT, [11])
  - Most homely, inviting, or cherished experience of one's life (N,N-DMT, [11])
  - Most nightmarish experience of one's life (N,N-DMT, [11])
  - Most exquisite, perfect, or special experience of one's life (N,N-DMT, [11])
  - Worst experience of one's life (N,N-DMT, [11])
  - Heavenly — Feeling that one has gone to heaven and come back (Ibogaine, [6])
  - Bizarre — "most bizarre thing I’ve ever experienced" (Ibogaine, [6])
  - Cherishing — "It was an experience that I’ll cherish" (Ibogaine, [6])

---

[33] Changes in the experience of, relationship to, and interpretation of other emergent phenomena, such as the sense of them being good or bad or mixed, of them occurring on their own or with a sense of control and even mastery, of them being harmful or healing, of them being real or unreal, of them being of social value or not, etc.

Motivational[34]

Increased motivation to practice

Strong motivation to practice (Concentration on visual object, [d])

Changes in motivation or goal NOS Buddhist meditation NOS, [p])

[34] Related to changes in one's motivations, intentions, or goals, e.g. in the context of emergent practice or in daily life. Some publications use the term "conative," but given the historical uses of its latin root *conatus*, *e.g.* within Baruch Spinoza's philosophical works, where it refers to something much more fundamental than individual goals and motivations, more akin to a fundamental, ontological or "cosmic" impetus that animates the entire process of becoming — an acception which influenced thinkers like Schopenhauer and Nietszche in forming their respective notions of *Will-to-live*, and *Will-to-power* — it seems more straightforward to use the simpler term of *motivation*.

Motor/Kinetic[35]

Balance impairment (N,N-DMT, [11])

Difficulty walking (Ibogaine, [6])

Impaired psychomotor coordination (N,N-DMT, [11]; Ibogaine, [6])

Involuntary movements (Meditation NOS, [g]; Buddhist meditation NOS, [p], [e])

- **Adopting unusual bodily postures involuntarily** (Buddhist meditation NOS, [q]; Buddhist meditation NOS, [e])
  - **Spontaneous hands and leg movements** (Meditation NOS, [k])
  - **Spontaneous […] back-bending** (Buddhist meditation NOS, [e])

- **Convulsive- or athetoid-type movements, shivering, shaking, jumping, jerking** (Buddhist meditation NOS, [e]; N,N-DMT, [11]; Psilocybin, [2])
  - **Body shaking/trembling** (Psilocybin, [2])
  - **Body vibrating "very very fast"** (Meditation NOS, [k])
  - **Jumpiness** (Buddhist meditation NOS, [e])
  - **Shaking** (Buddhist meditation NOS, [e])
  - **Whole-body jerking** (Buddhist meditation NOS, [e]) or **Involuntary jerks** (Meditation NOS, [g])

- **Teeth chattering** (Meditation NOS, [k])

Paralytic effects ((K-hole) Ketamine, [20]; N,N-DMT, [11])

---

35 Effects on the movement of the body, such as it seeming to freeze, become more or less coordinated, or move spontaneously in patterns, or apparently at random.

Paradigmatic[36]

General Beliefs or Views

Delusional, irrational, or paranormal beliefs[37] (Buddhist meditation NOS, [p])

Gaining a stronger conviction in the fruitfulness of one's spiritual endeavors (Anthroposophic Meditation, [u])

"Can be a consequence of insights" (Anthroposophic Meditation, [u])

Increased lack of conviction or doubt (Anthroposophic Meditation, [u])

"can arise if the practice leads to no specific results or insights" (Anthroposophic Meditation, [u])

Increased sense of **Significance** (Spontaneous NOS, [δ])

Everything seemed significant (Spontaneous NOS, [δ])

Sense of significance one is unable to explain (Spontaneous NOS, [δ])

Sense of **Inevitability** one is unable to explain (Spontaneous NOS, [δ])

Selfviews

Changes in how one views the relationship between their narrative self and other aspects of their experience (Buddhist meditation NOS, [q])

Sense of clarity about there being no self, about not being a fixed and abiding self (Buddhist meditation NOS, [q])

Worldviews

Change or transformations in worldviews (Esketamine, [5]; Ibogaine, [6]; N,N-DMT, [11]; Buddhist meditation NOS, [p]; Anthroposophic Meditation, [u]; Buddhist meditation NOS, [q]; N,N-DMT, [11]; Coma/Accident NOS, [α]; Accident/Emergency NOS, [ε])

New metaphysical beliefs

---

[36] Alterations in the way we fundamentally conceive of and relate to many aspects of experience and the world, which may include alterations to our understanding of and relationship to philosophy, ontology (what truly "is"), epistemology (how we know what truly "is"), how things truly work, and nosology (diagnosis). This is the level of high-level views, a person's deep "schemas" or constructions of reality, and sense-making.

[37] We do not necessarily endorse terms such as "delusional", "irrational", or "paranormal" beliefs, which carry ontological and (implicit) value judgments. While common in the biomedical discourse on the EPEEs of interest here (see e.g. Kaselionyte, J., & Gumley, A. (2019). Psychosis or spiritual emergency? A Foucauldian discourse analysis of case reports of extreme mental states in the context of meditation. *Transcultural Psychiatry*, *56*(5), 1094–1115. https://doi.org/10.1177/1363461519861842), we believe more nuance is needed, as these sorts of normative judgments can be needlessly pathologizing. Essential questions of politics of knowledge and normativity arise here. A similar critique can be made for much of the non-biomedical terminology which has been used in recent research, for instance in transpersonal psychology. See our Documentation/Definitions article for more detail.

Becoming "as sure that there is a spiritual world as that there is a table that they can touch in front of oneself" (Anthroposophic Meditation, [u])
Feeling that an entity encounter existed, at least in part, in some other dimension or reality (N,N-DMT, [11])
Feeling that an encountered entity continued to exist after an encounter (N,N-DMT, [11])
Feeling identical to an entity that was encountered (N,N-DMT, [11])
Strong **Sense that linear time is an illusion** (Accident/Emergency NOS, [ε])
Positive philosophical or spiritual insights (Esketamine, [5]; Ibogaine, [6]; N,N-DMT, [11]; Coma/Accident NOS, [α]), such as the metaphysical insight of **"Oneness with things"**, possibly involving emotional and perceptual aspects ("feeling a strong connection" or a "oneness" with things; **Perception of the "interwoven" nature of everything** (Ibogaine, [6]) or sense of **Harmony, unity or connection with the universe** (Esketamine, [5])

> "It was that realization of oneness with things… I felt just a connection… for some reason on ibogaine… I felt like a really strong connection with all that, almost like a delicacy. Everything was interwoven so perfectly, but it's so delicate, if any one thing was different it would be just a totally different scenario." (Ibogaine, [6])

Extreme insightfulness (N,N-DMT, [11])
Sense of harmony or unity with the universe (Coma/Accident NOS, [α])

Ontological Shock (N,N-DMT, [11])
Sense that the experience was mind-shattering or mind blowing (N,N-DMT, [11])

Perceptual[38]

Structural Modulations

Auditory

Changed perception of spatial location of sounds — *e.g.* Hearing voices as if far away (Epileptic Seizure, [β])

NOS **modifications of auditory perception** (Salvia divinorum, inhaled, [15])

Slowed auditory perception — *e.g.* Hearing voices as if slowed down (Epileptic Seizure, [β])

"Envelope" of phenomena[39]

Dissolution of objects of perception

Phenomena perceived as constituted by components (Theravadā śamatha and vipassanā Meditation, [h])

Direct perception of phenomena as a conjunction of elements with the characteristic of extension, cohesion, kinetic energy, motion, space, and at times, consciousness (Theravadā śamatha and vipassanā Meditation, [h])

Dissolution of objects of perception into their "constituents" (Theravadā śamatha and vipassanā Meditation, [h])

Dissolution of the perception of compactness or solidity concerning self and external objects (Theravadā śamatha and vipassanā Meditation, [h])

Dissolution of seeming "unit-components" of phenomena (Theravadā śamatha and vipassanā Meditation, [h])

"Components" of phenomena perceived as being in permanent flux (Theravadā śamatha and vipassanā Meditation, [h])

Sensations appear as disaggregated into small or very fine particles or vibrations (Buddhist meditation NOS, [i]; Theravadā śamatha and vipassanā Meditation, [h])

---

[38] Alterations in the quality, capability, phase, apparent sampling rate, scope, and shape of perception and attention (structural modulations) and novel perceptual content.

[39] In this case, this refers to the spatial or temporal form of phenomena. The term "envelope" is borrowed from music and describes the different phases of a sound (attack — sustain — decay — release), which can have various shapes. Here we also apply it to the "spatial" disaggregation of *gestalts*.

External objects and notion of I, me, or mine, dissolving into mere processes of physical and mental components interacting with each other (Theravadā śamatha and vipassanā Meditation, [h])

Endings of sensations can become predominant (Buddhist meditation NOS, [i])

Sensations can have a ratchet-like or mechanical quality (Buddhist meditation NOS, [i])

Sensations can have a prickly quality (Buddhist meditation NOS, [i])

General

Direct perception that nothing is permanent (Theravadā śamatha and vipassanā Meditation, [h])

Increased perceptual abilities (Anthroposophic Meditation, [u]; Meditation NOS, [g]; MBSR/Buddhist meditation NOS, [c]; Buddhist meditation NOS, [p]; N,N-DMT, [11]), such as **perceptual clarity**, *e.g.* "extreme sense of clarity or brightness" (N,N-DMT, [11]), or **perceptual hypersensitivity** (Meditation NOS, [g]; MBSR/Buddhist meditation NOS, [c]; Buddhist meditation NOS, [p]); **Becoming more sensitive and attentive to percepts** (Anthroposophic Meditation, [u]); **Excessive hypersensitivity (feeling "vulnerable")** (Anthroposophic Meditation, [u]); **Sense that one can effortlessly perceive everything** (Anthroposophic Meditation, [u])

Experiencing the arising and passing of physical and mental phenomena (Theravadā śamatha and vipassanā Meditation, [h])

Sense that perception is free of "conceptualized notions", such as objects or self (Theravadā śamatha and vipassanā Meditation, [h])

Sense of perceiving in an unbiased way (Theravadā śamatha and vipassanā Meditation, [h])

Sense of perceiving the process of "creating moment to moment the seeming reality in which one lives" (Theravadā śamatha and vipassanā Meditation, [h])

Surroundings and objects beyond immediate situation become less vivid and apparent (Accident/Emergency NOS, [ε])

Perceptual speed

Noticeable to tremendous increase in number of sensations observed per second (Buddhist meditation NOS, [i])

Dramatic slowdown in number of sensations observed per second following prior increase (Buddhist meditation NOS, [i])

Subject-to-object perceptions

Changed representation of location of "the observer"

Sense that instead of looking from the front of one's head, one is looking "from behind, through one's eyes" (Transcendental meditation, [d])

Feeling disoriented on account of a loss in the unity of one's experience (Buddhist meditation NOS, [q])

Feeling like one's consciousness became a kaleidoscope (Buddhist meditation NOS, [q])

Increased sense of perceptual duality

Feeling of "watching" (Transcendental meditation, [d])

"State has more the quality of "observing it" than experiencing it" (Transcendental meditation, [d])

Sense that "awareness is going beyond the physical senses" (Meditation NOS, [v]) or that one is **sensing something across a distance** (Meditation NOS, [v]) or **of seeing with eyes closed, "through one's eyes" or with a third eye** (Transcendental meditation, [d]; N,N-DMT, [11])

Sense of "experiencing awareness in the past or in the future" (Meditation NOS, [v])

Novel content

Auditory

Other **Hearing-like experiences** (Meditation NOS, [g]; Anthroposophic Meditation, [u]; Meditation NOS, [g]; Ayahuasca, [3])

Hearing things that others do not hear (Ayahuasca, [3])

Auditory "Hallucinations" (Meditation NOS, [g])

Loud noise connected with OBE (Anthroposophic Meditation, [u])

Hearing **Music** (Meditation NOS, [v]; Anthroposophic Meditation, [u]; Salvia divinorum, inhaled, [15]; NDE, [γ]; Epileptic Seizure, [β]) that was not in the physical environment

"Something that is like a song" (Anthroposophic Meditation, [u])

"A giant organ of angel voices, ranging from deep, deep, deep, bass tones to tones that are so high that they can hardly be heard [. . .]. And it was very plastic, melodic, and harmonic" (Anthroposophic Meditation, [u])

Sense of hearing **Voices** or **Receiving "communications"** in the absence of a person speaking as if they were real external perceptions (Anthroposophic Meditation, [u]; Meditation NOS, [k]; Meditation NOS, [v]; N,N-DMT, [11]; Ibogaine, [6]; Salvia divinorum, inhaled, [15]; Coma/Accident NOS, [α])

"Hearing a voice whispering in the wind" (Anthroposophic Meditation, [u])

Hearing voices while meditating (Meditation NOS, [k])

Unusual hearing or vision (Meditation NOS, [k])

Hearing Voices that were not in the physical environment (Meditation NOS, [v])

Sense of being verbally addressed by presences or beings (Salvia divinorum, inhaled, [15]) or of Receiving "communications" (Anthroposophic Meditation, [u])

Hearing an unidentifiable voice (Coma/Accident NOS, [α])

Mental

Mental images of blood and death and of cutting - being soaked in the images like a dream (Meditation NOS, [k])

Memory- or thought-like images (Anthroposophic Meditation, [u])

Quasi-visual imagery (Anthroposophic Meditation, [u])

Multimodal or indeterminate

Body feeling of the same nature as light (Buddhist meditation NOS, [o]), sense that **Light is emanating from the body** (Buddhist meditation NOS, [o]), or **feeling surrounded by a brilliant light** (Coma/Accident NOS, [α])

Feeling that one's body 'was breaking apart into sparkles and like electrical sparks were being sent off everywhere in all directions' (Buddhist meditation NOS, [o])

Feeling something crackling in the air (Meditation NOS, [v])

Increased sense of openness (Concentration on visual object, [d])

Sense of being totally open to everything (Concentration on visual object, [d])

Multimodal images (Anthroposophic Meditation, [u])
- Cold (Anthroposophic Meditation, [u])
- Healing (Anthroposophic Meditation, [u])
- Invigorating (Anthroposophic Meditation, [u])
- Informative (Anthroposophic Meditation, [u])

Paradoxical perceptions
- Sound perceived as "loudly silent" (Accident/Emergency NOS, [ε])

Perceiving concepts (Concentration on visual object, [d])
- Sense that Nature is breathing (Concentration on visual object, [d])
- Sense that one is able to feel Nature breathe (Concentration on visual object, [d])

Perceiving a **Void** (N,N-DMT, [11]) or **The "void"** (Esketamine, [5])
- White (N,N-DMT, [11])
- Golden (N,N-DMT, [11])
- Black (N,N-DMT, [11])
- Void NOS (N,N-DMT, [11]; Esketamine, [5])

Sense of being attacked (Anthroposophic Meditation, [u])

Sense of receiving new capacities (Anthroposophic Meditation, [u])

Sense of being protected (Anthroposophic Meditation, [u])

Sense of receiving guidance[40] (Anthroposophic Meditation, [u])

Sensing "Moods" or "atmospheres" (Anthroposophic Meditation, [u]; Jhāna meditation, [t])
- Sensing the "moods of nature" (Anthroposophic Meditation, [u])
- Sense of wisdom (Anthroposophic Meditation, [u])
- Sense of mercy (Anthroposophic Meditation, [u])
- Sense of dignity (Anthroposophic Meditation, [u])
- Sense of radiance (Anthroposophic Meditation, [u])
- Sense of freshness (Anthroposophic Meditation, [u])
- Sense of dryness (Anthroposophic Meditation, [u])
- Sense of bitterness (Anthroposophic Meditation, [u])
- Sense of Love (Anthroposophic Meditation, [u])
- Sense of intimacy (Anthroposophic Meditation, [u])

[40] This and previous descriptors could be seen as "proto-visionary", but they seem to suggest something less elaborate or immersive, so we decided to include it here.

Sense of Suffering (Anthroposophic Meditation, [u])
Sense of health (Anthroposophic Meditation, [u])
Sense of vitality (Anthroposophic Meditation, [u])
Sense of warmth (Anthroposophic Meditation, [u])
Sense of coldness (Anthroposophic Meditation, [u])
Sense of beauty (Anthroposophic Meditation, [u])
Sense of clarity (Anthroposophic Meditation, [u])
Safety (Jhāna meditation, [t])
Coziness (Jhāna meditation, [t])
Seclusion (Jhāna meditation, [t])
Magic (Jhāna meditation, [t])

Unitive-feeling states (Buddhist meditation NOS, [i]) or general sense of **Cohesion** (Jhāna meditation, [t]), sense of **Absorption** (NOS Christian practice, [w]), experiences of **"Jhāna"** (rapturous or peaceful states of attentional absorption) (Theravadā śamatha and vipassanā Meditation, [h])

Proprioceptive

Sense that "a very potent force split one's body in two halves" (Salvia divinorum, inhaled, [15])
Seeing one's body as a block of light (Theravadā śamatha and vipassanā Meditation, [h])

Subject-to-subject perceptions[41]

---

[41] The experiences here seem to involve a specific mode of attention, which consists in perceiving what appears as another *subject*, rather than an *object*. Husserl considered this a specific *mode* of intentional consciousness and called it the *welcoming* mode — that is, the disposition to relate to *alter egos* (Bitbol, M. (2014). *La conscience a-t-elle une origine ? Des neurosciences à la pleine conscience, une nouvelle approche de l'esprit*. Editions Flammarion, p.128). Here we call this "subject-to-subject perception". So-called "entity encounters," or "sensed presences," could be seen, from this perspective, as belonging to that type of "interest", perceptual or attentional mode. We all share the experience of momentarily perceiving an inanimate object, say, a rock, as being, say, a dog or a cat, and of then realizing what we saw is in fact not a dog but a rock. This produces a massive shift in our perception and our mode of relating to that perception. Similarly, we all share the experience of perceiving a "presence" when looking at a dark corridor when our imagination is excited by fear. In the context of so-called "relational-interactive" experiences (Strassman, *The Spirit Molecule*), instead of focusing on what is happening in terms of content — the *whom* or *what* I am perceiving — which may well be very impactful and important on its own terms, we can also focus on the fact that this "mode of attention", a subject-to-subject relationship, is what is manifesting and modulating experience in interesting and personal ways. One could look at Winkelman (2018)'s comments on entity encounters in the context of psychedelic experiences, while his account is slightly different than mine (ie, it is not phenomenological but based on cognitive theory), the overall explanatory thrust is quite similar to what I am saying here: these experiences may represent more profound and long lasting instances of a very natural form of perception in humans, which is to "subjectivize," and often anthropomorphize, objects of perception. The present remark is not to be taken as a value judgment on our part, but rather as a form of phenomenological bracketing and displacement of attention (reduction).

Experiencing a somatic "energy" as autonomous, possessing its own agency and volition and performing intentional actions on the body (Buddhist meditation NOS, [e])

Perceiving or sensing a presence or presences (Anthroposophic Meditation, [u])

"Like standing in front of an animal or seeing a fish in an aquarium" (Anthroposophic Meditation, [u])

Sense of being watched (Anthroposophic Meditation, [u])

Taste and Smell

Smell and taste "of Christ", associated with emotional intensity and intimacy (Anthroposophic Meditation, [u])

Burnt smell before a challenging sensed presence appeared (Anthroposophic Meditation, [u])

Touch

Sense of receiving a "spiritual touch" (Anthroposophic Meditation, [u])

Sense of **Cleansing sensations** (cleansing of "the brain") (Ibogaine, [6])

Sense of **Localized activity** (Ibogaine, [6])

Visual

Seeing short to long-lasting, simple to highly complex and multidimensional images sometimes called **"hallucinations", visions, "dysperceptions", "visuals", "imagery", or "illusions[42]"** (Anthroposophic Meditation, [u]; Meditation NOS, [g]; Meditation NOS, [v]; Buddhist meditation NOS, [p]; [i]; Mindfulness, [a]; Hindu Meditation NOS, [m]; NOS, [w]; N,N-DMT, [11]; Ibogaine, [6]; Ayahuasca, [3]; Salvia divinorum, inhaled, [15]; Epileptic Seizure, [β]), such as:

Ancient and/or culture-specific imagery (N,N-DMT, [11])

Animal visuals (N,N-DMT, [11])

Tentacles (N,N-DMT, [11])

Closed eye visuals (CEVs) NOS (N,N-DMT, [11])

Death imagery (Buddhist meditation NOS, [i])

[42] Again, we do not necessarily endorse terms such as "hallucinations" or "illusions" which carry similar ontological and (implicit) value judgments.

Cartoon-type visuals (N,N-DMT, [11])

Crystals (Anthroposophic Meditation, [u])

Digital or alien visuals (N,N-DMT, [11])

Faces (Anthroposophic Meditation, [u]; N,N-DMT, [11]; Ibogaine, [6]) or **Eyes** (N,N-DMT, [11]; Ibogaine, [6])

Figures (Anthroposophic Meditation, [u])

Flower(s) or lotus (N,N-DMT, [11])

Fractals, geometric shapes and patterns (Meditation NOS, [v]; N,N-DMT, [11]; Ibogaine, [6]; Salvia divinorum, inhaled, [15]; Epileptic Seizure, [β])

- Chrysanthemums (N,N-DMT, [11])
- "Everything would start to look like a roulette wheel" (Ibogaine, [6])
- Kaleidoscopes (N,N-DMT, [11])
- Geometric patterns (Salvia divinorum, inhaled, [15])
- Mandalas (N,N-DMT, [11])
- Other NOS (N,N-DMT, [11])
- Seeing geometric colored shapes (Epileptic Seizure, [β])
- "Sacred geometry" (N,N-DMT, [11])
- Spinning circle (Ibogaine, [6])

Seeing insects (Epileptic Seizure, [β])

Luminous **walls** and **surfaces** (Salvia divinorum, inhaled, [15])

Metallic objects (Salvia divinorum, inhaled, [15])

Moving perceptions

- **Visuals folding-in on themselves** (N,N-DMT, [11])

Open eye visuals (OEVs) (N,N-DMT, [11])

Other beings (Anthroposophic Meditation, [u])

Other visuals (Meditation NOS, [v])

Satanic visuals (N,N-DMT, [11])

Seeing things that others do not see (Ayahuasca, [3])

Skulls, skeletons, or other human anatomy (N,N-DMT, [11])

Symbols (Meditation NOS, [v]; N,N-DMT, [11]; Ibogaine, [6]), **Writing, scripture, or hieroglyphs** (N,N-DMT, [11]; Ibogaine, [6])

> "a trilogy symbol" that was "joyful" and symbolized "mind, body, and spirit" (Ibogaine, [6])

Tunnel, window-like visions (Salvia divinorum, inhaled, [15]) or Tunnel-type structures (N,N-DMT, [11])

Various degrees of clarity and intensity (Anthroposophic Meditation, [u])

> **Sensory-like visual images** (Anthroposophic Meditation, [u])

Webs or grids (N,N-DMT, [11]), **Lines** (Anthroposophic Meditation, [u]; Epileptic Seizure, [β]), wavy lines (Epileptic Seizure, [β])

Physiological[43]

Arthromyalgical

Jaw clenching (MDMA, ingested, [22])
Coughing/wheezing (Ayahuasca, [3])
Stiff/swollen joints (Ayahuasca, [3])

Cardiorespiratory[44]

Cardiac changes (Buddhist meditation NOS, [p]; Meditation NOS, [g]; Psilocybin, [2]; Transcendental Meditation, [w]; N,N-DMT, [11]; Epileptic Seizure, [β]) — *e.g.*:

Decreased heart rate (Transcendental Meditation, [w])
Heart beating more strongly, irregularly or skipping beats (Psilocybin, [2])
Increased heart rate (Meditation NOS, [g])
Sense of tachycardia or dysrhythmia (N,N-DMT, [11])
Palpitations (Epileptic Seizure, [β])

Chest

Chest pressure (N,N-DMT, [11]) or **Weight** (Psilocybin, [2])
Cough or lung harshness (N,N-DMT, [11])

Breathing

Breathing changes (Buddhist meditation NOS, [p]; Meditation NOS, [v])
Breathing more deeply (Meditation NOS, [v])
Breathing more shallowly (Meditation NOS, [v])
Breathing becoming somehow different than usual (Meditation NOS, [v])
Breathing difficulties (N,N-DMT, [11]; Ayahuasca, [3])
Choking sensation (N,N-DMT, [11])
Dyspnea, tachypnea, or apnea (N,N-DMT, [11])
Reduced oxygen consumption (Transcendental Meditation, [w])

Gastrointestinal

Abdominal pressure or weight (Psilocybin, [2])

Appetitive changes (Buddhist meditation NOS, [p]), **loss of appetite** (Meditation NOS, [g])

---

[43] Alterations of our material bodies and their quality and function, body temperature, heart rate and its variability, respiration, sleep, energy level in the ordinary sense, and measurable changes on EEG, MEG, fMRI, and other neuroimaging and measurement devices. This may apply to individuals or groups of individuals. See Medical below Included in the present inventory are physiological effects with a subjective component. Measures of physiological signs not accessible to introspection, such as EEG or blood testing, were excluded.
[44] One must obviously be very careful to not presume cardiac, respiratory, neurologic, endocrine, metabolic, toxicologic, and related signs and symptoms are necessarily purely practice or psychedelic related, for example, nor benign, and must keep an appropriate clinical differential diagnosis and index of suspicion, as with all of these phenomena.

Diarrhea (Meditation NOS, [g])

Feeling **'Butterflies'** (Epileptic Seizure, [β])

Gastrointestinal distress (Buddhist meditation NOS, [p]; Meditation NOS, [g])

Fecal urgency or incontinence (N,N-DMT, [11])

Nausea and/or disgust (Meditation NOS, [g]; Anthroposophic Meditation, [u]; Buddhist meditation NOS, [p]; [i]; N,N-DMT, [11]; Ibogaine, [6]; Ayahuasca, [3]; MDMA, serotonergic psychedelics, [4]; Epileptic Seizure, [β]), which can be movement induced (Ibogaine, [6])

Vomiting (N,N-DMT, [11]; Ibogaine, [6]; Ayahuasca, [3]; Ingested Ayahuasca, [9])

General/other

Adverse bodily "states" NOS (Meditation NOS, [g])

Analgesia (NDE, [γ]) or **Painlessness** (Coma/Accident NOS, [α])

Anesthesia (Ketamine, [12])

Elimination or attenuation of withdrawal symptoms (in addicts) during ibogaine experiences (Ibogaine, [6])

> "I would've been tearing my hair out, you know, hot and cold and crazy with withdrawal symptoms without ibogaine. (Ibogaine, [6])"
> "It was something else man, I can still remember the feeling of it just like, I'm not sick, how am I not sick?...I was like, dude, I haven't had morphine in apparently 36 hours, haven't had anything in 36 hours and I feel fine? This is crazy, this is what I was hoping would happen… nothing I've ever done before has worked like this. I've been to treatment four other times I think, and I was like, this is not the way this goes… but I did not have any withdrawal symptoms other than lack of sleep." (Ibogaine, [6])

Feeling sore (Ibogaine, [6])

Increased or decreased physical energy[45] (Jhāna meditation, [t]; Buddhist meditation NOS, [i]; Ibogaine, [6]) or **Strength** (Spontaneous NOS, [δ])

- **Body energized** (Jhāna meditation, [t])
- **Fatigue or weakness** (Buddhist meditation NOS, [p]; Ibogaine, [6]; Epileptic Seizure, [β])
  - Feeling wiped-out (Ibogaine, [6])

[45] This refers to the general sense of feeling energized, not specifically energetic-type somatic experiences, which are in the "Energetic" section.

Physically taxing experience (Ibogaine, [6])
Feeling one's energy drained (Epileptic Seizure, [β])
Weakness (Epileptic Seizure, [β])
"I felt the strength of ten men - over next few days it wore me down and I had to give up working" (Spontaneous NOS, [δ])

Physical Pain (Meditation NOS, [g]; Buddhist meditation NOS, [p]; [e]; [i]; Mindfulness, [a]; Psilocybin, [2]; Ayahuasca, [3]; N,N-DMT, [11]; Serotonergic psychedelics and MDMA [4]; Classical Psychedelics in lifetime users, [19])
Abdominal (Meditation NOS, [g]; Ayahuasca [3])
Chest (Ayahuasca [3])
Head pressure, headache, or migraines (Meditation NOS, [g]; Buddhist meditation NOS, [p]; Serotonergic psychedelics and MDMA [4]; Ayahuasca [3]; Classical Psychedelics in lifetime users, [19]; N,N-DMT, [11]; Spontaneous NOS, [δ]; Epileptic Seizure, [β])
"Real sharp pain on top of my head" (Spontaneous NOS, [δ])
Headaches (Epileptic Seizure, [β])
Pounding in one's head (Epileptic Seizure, [β])
Tightness in the head (Epileptic Seizure, [β])
Heart (Buddhist meditation NOS, [e]; Buddhist meditation NOS, [e])
Piercing pain in the **Heart** (Buddhist meditation NOS, [e])
"Feeling like there is a dagger or something embedding itself in the heart from below" (Buddhist meditation NOS, [e])
Neck pain (Meditation NOS, [g])
Lower back (Meditation NOS, [g])
Knee pain (Meditation NOS, [g])
Muscle aches (Ayahuasca, [3]; Meditation NOS, [g])
Muscle soreness (Meditation NOS, [g])
Physical suffering NOS (Psilocybin, [2])
Stomach pain (Meditation NOS, [g]; Epileptic Seizure, [β])

Pressure, tension or release of pressure, tension (Buddhist meditation NOS, [p]; Meditation NOS, [g])
Head pressure (Buddhist meditation NOS, [p])

Rapture (physical) (Buddhist meditation NOS, [i])

Reduced Stress Levels (Loving kindness, [w])

Relaxation (increase) (Loving kindness, [w]; Salvia divinorum, inhaled, [15])

Sedation (Ketamine, [12])

Sweating — excessive (diaphoresis) (N,N-DMT, [11]; Salvia divinorum, inhaled, [15]; Epileptic Seizure, [β])

Thermal changes (Buddhist meditation NOS, [p]; Buddhist meditation NOS, [e]; Salvia divinorum, inhaled, [15]; N,N-DMT, [11]; Spontaneous NOS, [δ]; Epileptic Seizure, [β]) or **Temperature dysregulation** (N,N-DMT, [11])

Intense heat (Buddhist meditation NOS, [e])

Feeling very hot (Spontaneous NOS, [δ])

Muscular

Multiple chronic muscle contractions (Meditation NOS, [g])

Aching muscles (Ayahuasca, [3])

Neurological

Seizures (Ayahuasca, [3])

Sensory systems

Visual focus problems (Meditation NOS, [g])

Facial **Numbness** (Epileptic Seizure, [β]) Stunned sensation - like a nerve block (Epileptic Seizure, [β])

Skin

Rash (Meditation NOS, [g])

Hives (Meditation NOS, [g])

Urinary System

Urinary urgency or incontinence (Meditation NOS, [g]; N,N-DMT, [11])

Psychological[46]

Affiliation

Empathic or affiliative changes (Buddhist meditation NOS, [p])
Change in doubt, faith, trust or commitment (Buddhist meditation NOS, [p])

Biographical memories

Déjà-vu (Epileptic Seizure, [β])

De-repression[47], i.e., old traumatic experiences appear in consciousness (Anthroposophic Meditation, [u]; Ingested Ayahuasca, [9]) or Becoming aware of repressed emotions (Ingested Ayahuasca, [9])

Recalling or re-experiencing disturbing or traumatic memories, **or stressful event in the past** (Meditation NOS, [g]; Buddhist meditation NOS, [p]; [e]; Meditation NOS, [a]; Mindfulness, [a]; Meditation NOS, [k]; Classical Psychedelics in lifetime users, [19]; Ingested Ayahuasca, [9])

- "Opening to all past trauma" (Buddhist meditation NOS, [e]), with a sense of beauty, or with associated difficulty

Re-experiencing memories (not traumatic) (Ibogaine, [6]; NDE, [γ]; Epileptic Seizure, [β])

- Re-experiencing many memories (Ibogaine, [6])
- Review of past events i.e. panoramic memories appearing all at once (NDE, [γ])
- Flashback to childhood (Epileptic Seizure, [β])

Reorganization of memories, including memories of past trauma/abuse (Ibogaine, [6])

Insights

Becoming aware of one's conditioning (e.g. the mental projections generated by emotions) (Theravadā śamatha and vipassanā Meditation, [h])

Sense of **Disidentification with one's conditionings** (Theravadā śamatha and vipassanā Meditation, [h])

---

[46] Alterations, both good and bad, in various aspects of our psychology, including the presentation, intensity, and relationship to our issues, as well as various presentations that can resemble and may sometimes appear to overlap with various mental illnesses as well as the wellness-related goals of positive psychology.

[47] In study [u], this de-repression happened as part of a "self-encounter" — in the sense described above — and ended up being a deeply transformative experience for the practitioner, affecting, among other things, their career path.

Emotional/psychological/relational insights (Anthroposophic Meditation, [u]; Spontaneous NOS, [δ]) or **Series of liberating emotional insights** (Ingested Ayahuasca, [9]) — example of such a sequences:

> "The subject became aware of repressed emotions (anger) and acquired novel psychological insights into their emotions and a given situation associated with it; subsequent transformation of anger into hurt, then a feeling of abandonment, then increased feeling of worthlessness; then the subject forgave their father and accepted their differences, coming out with a sense of having had a forgiving interaction with their deceased father who was then perceived as "released" (Ingested Ayahuasca, [9])
>
> "Recognition that I wasn't who I wanted to be — a wake-up call that my work oriented obsession was interfering with my relationship with my son" (Spontaneous NOS, [δ])

Sense of **Liberation from one's conditionings** (Theravadā śamatha and vipassanā Meditation, [h])

Feeling like a weight has lifted (Buddhist meditation NOS, [i])

New insights or creative solutions to problems that had been vexing one (Meditation NOS, [v])

Insight that moment to moment "mostly we live in total distortion because we are filtering everything through our habitual reactions, judgments and prejudices" (Theravadā śamatha and vipassanā Meditation, [h])

Difficulties

Aversion (Esketamine, [5])

Sense of **Bizarreness, strangeness or weirdness** (N,N-DMT, [11]; Esketamine, [5]), sometimes extreme (N,N-DMT, [11]; Esketamine, [5])

Sense of **Craziness** or **Insanity** (Psilocybin, [2]), Extreme sense of craziness or insanity (N,N-DMT, [11]), feeling like one is **Going crazy** (Meditation NOS, [k]; Meditation (various), [l])

Crisis (Anthroposophic Meditation, [u])

- **Biographical crisis** (Anthroposophic Meditation, [u])
  - **Encountering repressed and underdeveloped aspects of the personality** (Anthroposophic Meditation, [u])
- Crisis could also be associated with **Periods of inactivity and reduced functionality in daily life** (Anthroposophic Meditation, [u])

Crisis sometimes lead to **Breakthroughs in personal development** (Anthroposophic Meditation, [u])
Sense of a **Radical break with one's previous identity or reality** (Anthroposophic Meditation, [u])
Spiritual crisis (Anthroposophic Meditation, [u])
Sense of undergoing a "threshold experience", which refers to liminal state where the old is being left behind

"Depersonalization" (Meditation NOS, [g], Mindfulness, [a])

Difficulty managing troubling thoughts and feelings (Mindfulness, [a])

Disorientation (Esketamine, [5])

"**Dysphoria**" (Meditation NOS, [g])

Great psychological challenges (Buddhist meditation NOS, [i])

Feeling of being out of control (alarming) (Meditation NOS, [k])

"Mania" (Mindfulness, [a]; Buddhist meditation NOS, [e]; [i])

Paranoid negativistic delusional thoughts (Meditation (various), [l]; Hindu Meditation NOS, [m])

Panic Attacks (Mindfulness, [a])

Psychosis / Psychotic symptoms (Meditation NOS, [g]; Mindfulness, [a])

Intense anxieties (Meditation (various), [l])

"Ideas of reference" [sic] (Hindu Meditation NOS, [m])

Resistance, such as "resistance to effects of the drug", or "difficulty relinquishing control" (Esketamine, [5])

Suicidal ideation, feeling **Suicidal** (Hindu Meditation NOS, [m]; Meditation (various), [l]; Psilocybin, [7]) — *e.g.* because of excessive energetic experiences (Meditation (various), [l])

General

Increased relaxation (Loving kindness, [w])
Significantly greater self-awareness (Meditation NOS, [f])

Significantly greater acceptance of one's experience (Meditation NOS, [f])
Sense of absurdity of the "usual world" (Salvia divinorum, inhaled, [15])

> "It was very fun to check how absurd the usual world was. I was dying of laughter with the perspective of the existence of other worlds containing life"

Significantly increased sense of creativity (2C-B, Ingested, [14])
Reduced or absence of craving (in addicts) (Ibogaine, [6])
Reduced Stress Levels (Loving kindness, [w])

Personal narratives/Psychological Ego

Breakthroughs in personal development (Anthroposophic Meditation, [u])

Change in narrative self (Buddhist meditation NOS, [p]), Sense of a **Radical break with one's previous identity or reality** (Anthroposophic Meditation, [u])

Changes to personal issues, including "psychological insights in relation with personal issues" (Ibogaine, [6]), "alleviation of suffering related to reorganization of memories of past trauma/abuse" (Ibogaine, [6]), or **series of psychological insights into past issues** (Ingested Ayahuasca, [9])
Complete loss of personal and narrative identity (Buddhist meditation NOS, [q])
Confused sense of self (Buddhist meditation NOS, [q])
Disintegration or dissolution of the personality structures that support the 'story of me' or personal identity (Buddhist meditation NOS, [q])
"**Depersonalization**[48]" (Salvia divinorum, inhaled, [15])
Feeling "deeply wounded in the sense of one's identity" (Buddhist meditation NOS, [q])
Feeling like someone else (Meditation NOS, [v]) or **Experiencing a new identity** (Meditation NOS, [v])
Increased self-acceptance and acceptance of significant others as they are (Ibogaine, [6]; Ingested Ayahuasca, [9])
Loss of identity (Meditation NOS, [v])
Positively changed self-image (Ingested Ayahuasca, [9])
Previously implicit aspects of the 'story of me' becoming apparent, generally leading to a questioning, abandoning, or revision of that story (Buddhist meditation NOS, [q])
Sense of encountering a "higher self" (Anthroposophic Meditation, [u])
Sense of forgiving/forgiveness (Ingested Ayahuasca, [9])
Sense of transcending personal identity (Meditation NOS, [v])
Sense that one underwent "initiatory experiences" (Anthroposophic Meditation, [u])

Unusual ideation (Meditation NOS, [g]; Mindfulness, [a]; Hindu Meditation NOS, [m]; Meditation (various), [l]; Salvia divinorum, inhaled, [15])
Believing that one is invincible (Meditation (various), [l])

[48] Although common, this term and its companion, "derealization", are rather vague.

"Delusions or paranormal beliefs" NOS (Meditation NOS, [g], Mindfulness, [a])

Delusional convictions that one has caused the end of the world (Hindu Meditation NOS, [m])

Feeling like a special person — *e.g.* very powerful or like the Messiah (Spontaneous NOS, [δ])

Sense of having **Special powers** — *e.g.* that "one is making bottles rattle or making music distort when walking by pubs" (Spontaneous NOS, [δ]); or that one is able to "contact God", read people's minds and know everything (Meditation (various), [l])

Having a sense that one knows something special about the world (Meditation (various), [l])

"Illusions" (Mindfulness, [a])

Sense that "people from other planets are after one" (Meditation (various), [l])

Suspiciousness (Meditation NOS, [g])

Unusual ideation NOS (Salvia divinorum, inhaled, [15])

Semantic[49]

Disintegration of conceptual meaning structures (Buddhist meditation NOS, [p])

[49] Alterations in relationship to the meanings of words, concepts, and experiences.

Sensate[50]

Structural modulations

Auditory

Increased hearing sensitivity

Sensitive hearing (Classical Psychedelics in lifetime users, [19]; Salvia divinorum, [18]) — *e.g.* “the air-conditioner seemed louder...”, “...felt distracted by background sounds” (Salvia divinorum, [18])

General

Sensory overload (Spontaneous NOS, [δ])

Senses more vivid than usual (Coma/Accident NOS, [α]; Accident/Emergency NOS, [ε])

Mental

Decrease in the production of internal images (NOS, [w])

Tactile

Prominent, distinct body sensations (Self-boundary dissolution meditation techniques, [r])

Indistinct bodily sensations (Self-boundary dissolution meditation techniques, [r])

Imperceptible, non-local bodily sensations (Self-boundary dissolution meditation techniques, [r])

Transmodal[51]

Sensory modalities losing their specificity

"Sensation that [...] one is looking [...] through one's eyes" (Transcendental meditation, [d])

Visual

Visual **"Aliveness"** (Concentration on visual object, [d])

[50] Unusual sensate effects and experiences, typically visual, auditory, and somatic, but also olfactory, gustatory, mental, vestibular, proprioceptive, kinesthetic, transmodal, synesthetic, etc.

[51] This is a term that applies to qualities which are not specific to a given sense modality — for instance, rhythm, movement, size, space, time, are transmodal characteristics, as they can qualify visual experiences, tactile experiences, sounds, tastes, thoughts, etc., contrary to color, for instance, which is a specifically visual quality. See Petitmengin (2007).

"There is a definite aliveness in the environment, almost as if you're aware that there is consciousness in everything" (Transcendental meditation, [d])
"Almost as if objects were alive and breathing" (Transcendental meditation, [d])

Breaking up of visual percepts
Seeing one's body just as small particles (Theravadā śamatha and vipassanā Meditation, [h])

Environmental distortions, such as "room and wall distortion" (N,N-DMT, [11])

Expansion of the visual field (N,N-DMT, [11])

Blurring of contours of objects (Transcendental meditation, [d])
"Objects are less defined" (Transcendental meditation, [d])

Visual **"Breathing"** (Transcendental meditation, [d]; Concentration on visual object, [d]; N,N-DMT, [11])
"Almost as if objects were alive and breathing" (Transcendental meditation, [d])
A "breathing" quality to surroundings (Concentration on visual object, [d])

Increased **Brightness of the visual field** (Buddhist meditation NOS, [o])

Increased visual **Clarity** (Jhāna meditation, [t]; N,N-DMT, [11])

Clouded vision — *e.g.* Seeing as if through a film (Epileptic Seizure, [β])

Increased **Color Vividness** (Concentration on visual object, [d]; Salvia divinorum, [18]; N,N-DMT, [11]), **Brightness** (Jhāna meditation, [t]), **Intensity** (N,N-DMT, [11]; [1]) or **Contrast (**Salvia divinorum, [18])
Hyperintense colors (N,N-DMT, [11])
Intensification of colors (Psilocybin mushrooms, ingested, [1])
Increased vividness and contrast, e.g. “the contrast was more vivid” (Salvia divinorum, [18])

Disappearance of visual percepts (Concentration on visual object, [d]; Buddhist meditation NOS, [p], [e]))
Object of meditation "disappeared" (Concentration on visual object, [d])
Visual percepts falling away (Buddhist meditation NOS, [e])

Desolidification of objects (Transcendental meditation, [d]; Buddhist meditation NOS, [o])

"Objects are not as solid." (Transcendental meditation, [d])

Seeing energy instead of solid objects (Buddhist meditation NOS, [o])

Dissolution of visual percepts or objects (Buddhist meditation NOS, [p]; N,N-DMT, [11])

Distortions of objects — *e.g.* Object of meditation "turns to plastic" (Concentration on visual object, [d])

Glowing colors

Neon (N,N-DMT, [11])

Kaleidoscopic vision (Epileptic Seizure, [β])

Visual **"Melting"** (Concentration on visual object, [d]; N,N-DMT, [11])

Object of meditation "melts" (Concentration on visual object, [d])

Modifications of visual perception NOS (Salvia divinorum, inhaled, [15]; N,N-DMT, [11])

Monochromatic vision (Epileptic Seizure, [β])

Moving visual percepts (Transcendental meditation, [d]; (NOS, [w]))

"the whole objects seems to be moving on some level" (Transcendental meditation, [d])

Perceived motion in otherwise static inkblot images (NOS, [w])

Pointillistic vision (Transcendental meditation, [d])

"Objects are [...] like millions of little spots" (Transcendental meditation, [d])

Pixelation (Meditation NOS, [b]; Buddhist meditation NOS, [o]; N,N-DMT, [11])

Seeing "pixels" (Meditation NOS, [b])

Reality pixelating (Buddhist meditation NOS, [o])

Sense of a **Perceptual veil or curtain lifting/falling** (N,N-DMT, [11])

Visual **"Pulsating"** (Concentration on visual object, [d])

Everything was kind of pulsating (Concentration on visual object, [d])

Increased visual **Sensitivity** (Transcendental meditation, [d]; MBSR/Buddhist meditation NOS, [c]; Salvia divinorum, [18])

"It's like there's all kinds of little moving things." (Transcendental meditation, [d])

e.g. “the patterns on the curtain appeared more prominent...” (Salvia divinorum, [18])

Shaky vision (N,N-DMT, [11])

Shimmering of visual percepts (Transcendental meditation, [d])

"the whole objects seems to be moving on some level as if you looked through heat - how it shimmers" (Transcendental meditation, [d])

Visual **"Tracers"** (N,N-DMT, [11])

Tunnel vision (Transcendental meditation, [d])

A sort of tunnel vision associated with [change in vision] (Transcendental meditation, [d])

Visual **"Vibrating"** (Concentration on visual object, [d]; N,N-DMT, [11])

Boundaries between objects are vibrating but sharp (Transcendental meditation, [d])

Objects have a slight vibratory quality to them but don't lose their distinctness of outline (Transcendental meditation, [d])

Visual "**Warping"** (N,N-DMT, [11])

Visual **"Zooming out"** (Transcendental meditation, [d])

Slight change in one's vision, like a camera lens zooming out, a pulling back (Transcendental meditation, [d])

Sometimes there's a perceptual shift - like you're looking through a tube or a telescope - like you're looking at something from somewhere else (Transcendental meditation, [d])

Novel Content

Auditory

Endogenous sounds[52]

[52] Meaning sounds which were seemingly spontaneous and not produced by anything in the physical environment of the person.

Ringing-type sounds (Jhāna meditation, [t]; Meditation NOS, [v]; N,N-DMT, [11]; Ibogaine, [6]))

"Auditory carrier wave" (N,N-DMT, [11])

Buzzing sound (Meditation NOS, [v]; N,N-DMT, [11]; Ibogaine, [6])

Ears ringing loud (Ibogaine, [6])

Sense that the buzzing sounds is taking one into the full experience (Ibogaine, [6])

Crackling (N,N-DMT, [11]; Epileptic Seizure, [β]) — *e.g.* a sound "like Rice Krispies" (Epileptic Seizure, [β])

Droning (N,N-DMT, [11])

"**Electric**" sound (N,N-DMT, [11])

High-pitched sound (Jhāna meditation, [t]; N,N-DMT, [11])

Hissing (N,N-DMT, [11])

Humming (N,N-DMT, [11]; Meditation NOS, [v])

Muffled noise (Accident/Emergency NOS, [ε])

Noise NOS (NDE, [γ]),

Popping (N,N-DMT, [11])

Pulsing (N,N-DMT, [11]; Epileptic Seizure, [β]) — Pulsing noises (Epileptic Seizure, [β])

Static (N,N-DMT, [11])

"**Tunnel sound**" (Accident/Emergency NOS, [ε])

Vibrating (N,N-DMT, [11]; Ibogaine, [6]) — Intense vibration sounds (Ibogaine, [6])

Whining noise (N,N-DMT, [11])

Zapping (Ibogaine, [6])

Interoceptive[53]

Breath stops being perceived (Jhāna meditation, [t])

Feeling shaky inside (Psilocybin, [2])

Mental

Experiencing **Vivid mental imagery** (Buddhist meditation NOS, [e])

Seeing closed eyes visual imagery for the first time in one' s life (Ingested Ayahuasca, [9])

Proprioceptive/Kinesthetic[54]

Changes to **sense of Directionality**, *e.g.* centring/being lifted up/moving inwards (Jhāna meditation, [t]; Meditation NOS, [b]), e.g. **Sense of directionality in space becomes less clear** i.e. one's sense of oneself in relation to one's surroundings is less bodily/embodied (Meditation NOS, [b])

Changes to **sense of Space** (Epileptic Seizure, [β]; Self-boundary dissolution meditation techniques, [r]; Self-boundary dissolution meditation techniques, [r])

Feeling as if one has 'no sense of order' (Epileptic Seizure, [β])
Indeterminate self-world structure (Self-boundary dissolution meditation techniques, [r])
Other alteration of sense of location (ambiguous space) (Self-boundary dissolution meditation techniques, [r])

"Dissociation" from the body or body sensations (Buddhist meditation NOS, [q]; Salvia divinorum, inhaled, [15]; Esketamine, [5]; Ketamine, [20]), sense of **Observing body from outside** (Buddhist meditation NOS, [q])

Disruptions of the body scheme (Anthroposophic Meditation, [u]; Meditation NOS, [v]; Concentration on visual object, [d]; Buddhist meditation NOS, [p]; Buddhist meditation NOS, [e]; Buddhist meditation NOS, [q]; Self-boundary dissolution meditation techniques, [r]; Meditation NOS, [b]; N,N-DMT, [11]; Epileptic Seizure, [β])

Sense that **Body parts are disappearing** (Meditation NOS, [v])
Feeling like the **Body changed in shape or size** (Meditation NOS, [v])
NOS Change in sense of embodiment (Buddhist meditation NOS, [p])
Feeling more embodied (Buddhist meditation NOS, [e]; Anthroposophic Meditation, [u]), i.e., "more connected to one's body and

---

[53] Refers to the ability to feel the body's internal sensations, *e.g.* visceral sensations, or sensations from other organs.
[54] Here referring to felt sense of movement, body position, location — at a sensate level, the representative/perceptual level.

experiencing an embodiment of the environment in a broader sense" (Sparby, 2020)
Sense of being pulled, pressured, twisted or divided (Salvia divinorum, inhaled, [15]; Epileptic Seizure, [β])
Feeling like one does not have a body anymore or disembodied (Anthroposophic Meditation, [u]; Buddhist meditation NOS, [q])
Feeling like one has spilled out of their ordinary embodiment into the world such that there was no longer 'self', only objects (Buddhist meditation NOS, [q])
Changes in locatedness within the body schema, such as from behind the eyes to the middle of the head or to the heart (Buddhist meditation NOS, [q])
Sense of being relocated above, behind, to the side, or displaced in some way from the body (Buddhist meditation NOS, [q])
Sense that one's body is so spread that it's difficult to know where it is and what it is (Meditation NOS, [b])
Within body (only) (Self-boundary dissolution meditation techniques, [r])
Body and close surrounding (Self-boundary dissolution meditation techniques, [r])
Expansion into vast space (Self-boundary dissolution meditation techniques, [r])
Sense that conscious awareness of the body drops from the head into the heart, and then **a sense of being continually present in the heart center** (Buddhist meditation NOS, [q])
Sense that one's **Body is dissolving** (N,N-DMT, [11]) or **has dissolved** (Meditation NOS, [b])

Lateralization of effects, *e.g.* sense that effects are starting from a specific side of "reality" (Salvia divinorum, inhaled, [15])

"I had the sensation that the effects of the substance were approaching me, like an air blast, from the left"; "I felt like another reality, just as real or even more authentic, was coming from the right."

Loss of contact, perception or awareness of one's body (Anthroposophic Meditation, [u]; Transcendental meditation, [w]; Theravadā śamatha and vipassanā Meditation, [h]; Esketamine, [5]; Salvia divinorum, inhaled, [15])

Sensations of accelerating, falling, or moving at a high velocity (N,N-DMT, [11]), *e.g.* a "sense of being on a rollercoaster ride (Esketamine, [5])

Sense of **Floating** (Meditation NOS, [v]; Meditation NOS, [k]; Ketamine, [20])

Sense of **Floating out of body** (N,N-DMT, [11]), of **Spirit/Soul leaving body** (N,N-DMT, [11]), of **Falling away from body** (N,N-DMT, [11]), of **Falling through space or through the ground** (Buddhist meditation NOS, [q]), of **"disappearing" or of "dropping out"** – like falling into empty space (Meditation NOS, [b]), of being **Lifted out of one's body** (Concentration on visual object, [d]), of **Being separated from one's body** (Coma/Accident NOS, [α]; NDE, [γ]); "**Out of body experience**"[55] (Meditation NOS, [v]; Anthroposophic Meditation, [u]; Buddhist meditation NOS, [q]; Concentration on visual object, [d]; Salvia divinorum, inhaled, [15]; Ketamine, [20]; Spontaneous NOS, [δ]; NDE, [γ])

Out of body experiences during meditation can be both light and strong (Anthroposophic Meditation, [u])

Sense of **Lightness** (Meditation NOS, [v]; Jhāna meditation, [t]) or **Heaviness** (Meditation NOS, [v]; Ibogaine, [6]; N,N-DMT, [11])

Smell/taste

Endogenous smells (Meditation NOS, [v]; Epileptic Seizure, [β]; NDE, [γ]) — Smelling or tasting things that "were not physically there" (Meditation NOS, [v]), unusual olfactory phenomena (NDE, [γ])

Smelling sulfur (Epileptic Seizure, [β])
Smelling burning (Epileptic Seizure, [β])
Smelling watermelon (Epileptic Seizure, [β])
Smelling ammonia (Epileptic Seizure, [β])
Smelling pungent spices (Epileptic Seizure, [β])

Unpleasant taste (N,N-DMT, [11]; Epileptic Seizure, [β])

Unusual gustatory phenomena NOS (NDE, [γ])

Synesthetic[56]

Affective-tactile synesthesia — *e.g.* "Every breath was a loving experience - it was like breathing in love" (Spontaneous NOS, [δ])

Audio-tactile synesthesia (Anthroposophic Meditation, [u]; Ibogaine, [6])

"A voice appeared, "half-felt, half-heard"" (Anthroposophic Meditation, [u])

Visual-affective synesthesia (Anthroposophic Meditation, [u])

---

[55] See Sparby (2020): "Out of body experiences overlap in part with sleep paralysis experiences but do not necessarily happen during sleep or in a hypnagogic or hypnopompic phase"

[56] Refers to sensations which seem to belong to a blend of sense modalities with representations of one sense category in another.

"Perceiving the affective component of an image" (Anthroposophic Meditation, [u])

Visual-auditory synaesthesia (N,N-DMT, [11])

Visual-proprioceptive synesthesia (Salvia divinorum, inhaled, [15])

Objects perceived with eyes open or closed were felt as being associated with the body (Salvia divinorum, inhaled, [15])

For example, seeing external modifications in reality, like a wave, that affects or folds the person's body: “The effects of the substance were approaching me, like an air blast, from the left. (...) they were distorting the objects in the room and also my body and sensations”.

One person described the concept of a square present in the mind that was felt with the body: “A force was pressing the right side of my body (...), so my sensation was being a square. Visually I wasn’t seeing any image, but that square was conceptually present in my mind”

Visual-spatial synesthesia

Sense that **space is filled with color** (Buddhist meditation NOS, [e])

Visual-tactile synesthesia (Anthroposophic Meditation, [u])

"A tactile seeing" (Anthroposophic Meditation, [u])

Tactile

Body “load” (N,N-DMT, [11])

Buzz (N,N-DMT, [11])

Changes in bodily sensations NOS (Buddhist meditation NOS, [p]; Salvia divinorum, inhaled, [15])

Cold (Meditation NOS, [v]; Epileptic Seizure, [β])

Facial or oropharyngeal "paraesthesias" (N,N-DMT, [11])

Heat of **Warmth** sensations (Meditation NOS, [v]; Ibogaine, [6]; NDE, [γ], Epileptic Seizure, [β])

Localized sensations (Ibogaine, [6])

"At the back of the brain" (Ibogaine, [6])

Pressure (Meditation NOS, [v])

Scrubbing sensations (Ibogaine, [6])
Scrubbing of "the brain" (Ibogaine, [6])

Tingling (Salvia divinorum, inhaled, [15]; N,N-DMT, [11]; Esketamine, [5]; Meditation NOS, [v]; Epileptic Seizure, [β)

Vibrations (Salvia divinorum, inhaled, [15]; N,N-DMT, [11])

Rapture — Body "high" or body euphoria (N,N-DMT, [11])

Zapping sensations (Ibogaine, [6])
Sides, front and mostly back of the "brain" (Ibogaine, [6])
In "the brain" (Ibogaine, [6])

Transmodal
Feeling of a "reboot" (Buddhist meditation NOS, [i])

Sense of **Warmth** (Jhāna meditation, [t])

Vestibular[57]
Dizziness, disorientation or lightheadedness (Meditation NOS, [g]; Buddhist meditation NOS, [p], [e]; Anthroposophic Meditation, [u]; N,N-DMT, [11]; Ibogaine, [6]; Spontaneous NOS, [δ]; Epileptic Seizure, [β]), sometimes involving a sense of "**Spinning**" or "spinning around the room" (Meditation NOS, [g] Ibogaine, [6] ; Esketamine, [5]), or of **Spatial disorientation** (Ibogaine, [6]; Meditation NOS, [g]), **Dizziness, vertigo or syncope** (Buddhist meditation NOS, [p], [e]; Anthroposophic Meditation, [u]; Meditation NOS, [g]), sense of **Losing contact with the body** that got stronger when meditating (Anthroposophic Meditation, [u]), **Feeling intoxicated** or **Head "swimming"**(Epileptic Seizure, [β])

Sense of **Lightness** (sometimes experienced as positive and rapturous) (Anthroposophic Meditation, [u]; Ibogaine, [6])

Visual
Halos (N,N-DMT, [11]; NDE, [γ]), "**Auras"** (NDE, [γ]) or **Bands of color around people or objects** (Transcendental meditation, [d])

---

[57] Felt sense of balance and spatial orientation.

Endogenous **Lights or Colors with eyes-opened or closed**[58] (Concentration on visual object, [d]; Buddhist meditation NOS, [e]; [p]; [o]; [i]; Meditation NOS, [v]; Anthroposophic Meditation, [u]; Hindu Meditation NOS, [m]; N,N-DMT, [11]; Ibogaine, [6])

Discrete lightforms (Buddhist meditation NOS, [o]; Hindu Meditation NOS, [m]; Anthroposophic Meditation, [u]; N,N-DMT, [11]; Ibogaine, [6]; Coma/Accident NOS, [α]; Epileptic Seizure, [β])

Seeing a **Brilliant light** (Coma/Accident NOS, [α]) or **Bright light** (N,N-DMT, [11]; Ibogaine, [6])

Experiencing various **Bright colors** (Hindu Meditation NOS, [m]; Buddhist meditation NOS, [o]; Anthroposophic Meditation, [u])

Blue, purple, and red globes (Buddhist meditation NOS, [o])

White or blue lights (Buddhist meditation NOS, [o])

White light (N,N-DMT, [11]; Ibogaine, [6])

Silvery, blue or diamond-like colors (Anthroposophic Meditation, [u])

White spots or little stars (Buddhist meditation NOS, [o])

"Christmas tree"-like lights (Buddhist meditation NOS, [o])

Seeing **Flashing lights** (Epileptic Seizure, [β])

"Jewel"-like lights (Buddhist meditation NOS, [o])

Moon-shaped objects (Buddhist meditation NOS, [o])

Simple light impressions (Anthroposophic Meditation, [u])

Small radiant bursts (Buddhist meditation NOS, [o]) or **Exploding light** (Anthroposophic Meditation, [u])

---

58 These can be *superimposed on***,** or entirely *replace* perceptions of surroundings — *e.g.* "Object of meditation replaced by "golden light", then "green light", then "white light"" (Concentration on visual object, [d]).

Patterned and diffuse lights (Buddhist meditation NOS, [e]; Buddhist meditation NOS, [o]; Anthroposophic Meditation, [u])

Beam/stream of light (N,N-DMT, [11]; Ibogaine, [6])

Blinding light (Anthroposophic Meditation, [u])

Bursting with light (Buddhist meditation NOS, [o])

Curtain of light (Buddhist meditation NOS, [o])

Electrical sparks in all direction (Buddhist meditation NOS, [o])

Field of vision covered by intense color (Buddhist meditation NOS, [e])

Golden light that fills the sky (Buddhist meditation NOS, [o])

Lightning-strike-like, upward and radiating light (Anthroposophic Meditation, [u])

Lights always on (Buddhist meditation NOS, [o])

Ropes of shimmering (Buddhist meditation NOS, [o])

Rays of light that "go through everything" (Buddhist meditation NOS, [o])

Sexual[59]

Sexuality-related changes (Buddhist meditation NOS, [p]; Buddhist meditation NOS, [e])

- **Increased libido** (Buddhist meditation NOS, [e])
- **Sexual thoughts** (Buddhist meditation NOS, [e])

Sexual, intimate, or erotic-toned experience (N,N-DMT, [11])

[59] Changes to libido, sexual fantasies, orientation, and so on.

Social[60]

Perception/Representation of Self[61]

Increased sense of separateness, e.g. “I could see you and hear you, but I felt separated and distant from you...” (Salvia divinorum, [18])

Perception/Representation of others[62]

Decreased ability to identify facial expressions of anger (MDMA, ingested, [22])

Increased intensity required to identify anger compared to other negative emotions (MDMA, ingested, [22])

Increased sense of being positively regarded by others during social interactions (MDMA, ingested, [22])

Increased perception of others as empathic during social interactions (MDMA, ingested, [22])

Increased positive responses to happy faces (MDMA, ingested, [22])

Sense that other people can sense one's increased energy/heat (Spontaneous NOS, [δ])

Sociability

Decreased self-reported sociability (2C-B, Ingested, [14]; Psilocybin, ingested, [14]) or

Desire for isolation, e.g. “...I wished I didn’t have to answer questions...”, “..wished I was left alone...” (Salvia divinorum, [18])

Significant increase in friendliness (2C-B, Ingested, [14])

---

[60] Changes in our relationships to others, society, and our roles in society.
[61] During a social interaction/within a social context.
[62] During a social interaction/within a social context.

Spatial[63]

Sense of **Space being distorted from its usual mode** (Buddhist meditation NOS, [p]; Meditation NOS, [v])

Sense of **Spacelessness**, **of lacking a sense of space** or **sense of space disappearing/absent** (Christian Contemplative Prayer, [w]; Anthroposophic Meditation, [u]; NOS Buddhist practice, [w]; Transcendental meditation, [w])

Sense of **Being outside space** (Esketamine, [5])

Spatial disorientation (Meditation NOS, [g])

Simultaneous experience of "location and non-location" (Meditation NOS, [b])

[63] Related to emergent phenomena doing something to the sense of space itself, changing it, adding something emergent to it, etc.

Temporal[64]

Slowing of time perception (Meditation NOS, [v]; 2C-B, Ingested, [14]; Coma/Accident NOS, [α]; Accident/Emergency NOS, [ε]; NDE, [γ])

Time seeming to **Slow down** (Coma/Accident NOS, [α]; Accident/Emergency NOS, [ε]), **Pass more slowly** than usual (NDE, [γ]), or **Seeming longer** than usual[65] (Meditation NOS, [v]; Accident/Emergency NOS, [ε]) — *e.g.* Seconds turning into minutes or hours (Accident/Emergency NOS, [ε]), "Everything seemed to go in slow motion" (Accident/Emergency NOS, [ε]), Fast moving objects appearing to slow down (Accident/Emergency NOS, [ε])

Speeding of time perception (Meditation NOS, [v]; Psilocybin, ingested, [14]; Coma/Accident NOS, [α])

Time seeming to **Speed up** (Coma/Accident NOS, [α]), or **Seeming shorter** than usual (Meditation NOS, [v])

Suspensions of time perception (Christian Contemplative Prayer, [w]; Anthroposophic Meditation, [u]; NOS Buddhist practice, [w]; Esketamine, [5]; Accident/Emergency NOS, [ε])

Sense of **Being outside time** (Esketamine, [5])

Experience of **Eternity** (Esketamine, [5])

Suspension — Time seeming **Suspended** (Accident/Emergency NOS, [ε])

Sense of **Timelessness** (Anthroposophic Meditation, [u]; NOS Buddhist practice, [w]; Accident/Emergency NOS, [ε])

Sense of **Time stopping or disappearing** (Christian Contemplative Prayer, [w]; Accident/Emergency NOS, [ε]),

Other **Changes** or **Distortions in the perception of time** (Meditation NOS, [b]; Buddhist meditation NOS, [p]; Meditation NOS, [g]; Meditation NOS, [v]; Anthroposophic Meditation, [u]; N,N-DMT, [11]; Ibogaine, [6]; Salvia divinorum, inhaled, [15]; Coma/Accident NOS, [α]; Accident/Emergency NOS, [ε]),

Altered time perception NOS (Coma/Accident NOS, [α])

Sense of **Being stuck in a repetitive time loop** (N,N-DMT, [11]; Ibogaine, [6])

Losing track of time (Anthroposophic Meditation, [u])

---

64 Alterations in the perception of and understanding of time, including time seeming to speed up, slow down, stop, go backwards, loop, split, be lost, cease to apply as a construct altogether, or to have some sort of access to the past, future, or alternative timelines, including such concepts as retrocausality.

65 All of these formulations, although similar, carry slightly different meanings.

Strong **Sense that linear time is an illusion** (Accident/Emergency NOS, [ε])

Temporal disorientation (Meditation NOS, [g])

Time stops feeling relevant (Meditation NOS, [b])

Volitional[66]

Avolition (Buddhist meditation NOS, [p])

Change in effort or striving (Buddhist meditation NOS, [p])

Difficulty making decisions (Classical Psychedelics in lifetime users, [19])

Feeling of losing control over one's physical body (Anthroposophic Meditation, [u])

Lack of control or sense of losing control over experience (Concentration on visual object, [d]; Ibogaine, [6]; Spontaneous NOS, [δ])

> "I came to the realization that this is out of my control, I'm Terrified, there's nothing I can do about it, and once that happened, it was a much smoother ride." (Ibogaine, [6])

Passivity

> Passive state (Transcendental meditation, [d])
> 'The process took me over' (Meditation (various), [l])

Sense of **Being possessed, abducted, or invaded** (Spontaneous NOS, [δ]), or **Sense that one's body is being controlled** — *e.g.* by an energy (Buddhist meditation NOS, [e])

---

[66] Related to changes in our sense of control and agency, such as strengthening it, reducing it, eliminating it, transcending it, seeing through some sense of an illusion of it, being controlled by other people or entities, and other alterations.

Wakeful[67]

High wakefulness (Jhāna meditation, [t])

Intentionally-induced momentary extinction of experience[68] (Buddhist śamatha/vipassanā meditation, [n]; Buddhist meditation NOS, [i]; Vedic Yoga, [w])

Loss of consciousness (LSD, [10]; Spontaneous NOS, [δ]) **— While remaining physically active** (loss of awareness of surroundings/memory of actions) (LSD, [10])

Oblivion to external and internal environmental stimuli (Vedic Yoga, [w])

Syncope/Fainting (Meditation NOS, [g]; Ayahuasca, [3]), Fainting (Ayahuasca, [3])

---

[67] Related but different from arousal, as *e.g.*, feeling very awake/high wakefulness could be accompanied by either low or high arousal. One can even be very "awake" whilst being asleep, as in the case of lucid dreaming, or lucid dreamless sleep, which are reported in several publications under review. Conversely, some meditative phenomena (*e.g.* various forms of cessation of consciousness), seem to involve a momentary absence of any experiential content, which seems to be only marginally related to arousal, since the physiology of meditators who have been studied while in cessation for 90 min, didn't stop, although it slowed down (Laukkonen *et al.*, 2023). There still appeared to be some degree of arousal, from a third person perspective, even though, from the first-person perspective of the meditator, any form of awakeness or wakefulness had disappeared.

[68] In Buddhism, various forms of this are called Nirodha, Phala-Samāpatti, Nirodha-Samāpatti… It seems plausible that there are equivalent concepts to refer to such events — since these can hardly be called "states" or "experiences" — in various other traditions, and that these are often associated with the notion of *transcendence*.

Other dimensions[69]

Evolution/Diachronic patterns

Small-scale

Progressive transformation and disappearance of visual forms, followed by novel endogenous visual sensations (Concentration on visual object, [d]) : 1 - distortion; 2 - "melting"; 3 - disappearance; 4 - visual forms replaced by light; 5 - Light changes color : a - Golden; b - Green; c - White

Reaction to altered sense of embodiment and subsequent cognitive effects (Concentration on visual object, [d]): 1 - Feel like one is lifted out of one's body; 2a - Fear reaction to the modulation of experience; 2b - Lack of control over experience; 3 - Deciding to keep engage in the practice; 4 - Gradual subsiding of fear; 5 - Feeling a total clarity of mind, etc.

Onset of Ibogaine, [6]:

Phase (1) Zapping/buzzing sensations, sounds and synesthetic vibrations come up and intensify, in specific locations, with a sense that they are cleansing the area where they are happening, and leading to

Phase (2) Entering an alternate reality with complex visions and scenarios happening. .

Mid-scale

Vocational changes due to transformative psychological experiences in meditation. Self-encounter > De-repression of memories > Struggle with pangs of conscience > Deeply transformative experience > affects career path (Anthroposophic Meditation, [u])

MDMA, ingested, [11]: Effects appear between 30-60 min after ingestion with a general peak between 60-120 min ; elation was back to normal after about 240 min, while some subjective effects persisted beyond the 4h mark, such as feeling more loving, which may then be considered as an afterglow, but also physiological effects such as increased arterial pressure

Large-scale

Progressive normalization of previously remarkable new experiences

Experience progressively lost its remarkable character (Transcendental meditation, [d])

Acquired sense of normalcy of [an initially remarkable] experience (Transcendental meditation, [d])

Almost constant state of "depersonalization" that is not unpleasant anymore, with confusing episodes of sometimes shifting out of this (Transcendental meditation, [d])

Intensity

Low to moderate (Transcendental meditation, [d])

Equivalent to psilocybin **(2C-B, Ingested, [14])**

---

[69] This section contains little information, as the publications we reviewed often did not report on these dimensions.

High (in medium to high doses) (Salvia divinorum, inhaled, [15])
(Ibogaine, [6])
Extreme (N,N-DMT, [11]) — Extreme profundity or power of the experience (N,N-DMT, [11])

Duration

Salvia divinorum, inhaled, [15]: Short duration (< 20 min) with a rapid peak

MDMA, ingested, [11]: 4+ hours

2C-B, Ingested, [14]: Subjective effects abated in under 6 hours for 86.3 % of participants
Experiences reportedly associated with meditation had **durations ranging from minutes, tens of minutes, hours, weeks, to years/seemingly becoming permanent**

- A few minutes (Transcendental meditation, [d])
- Derealization gradually disappeared upon the resumption of normal activity (Transcendental meditation, [d])
- The experience of "derealization" lasted for several hours, disappearing gradually (Concentration on visual object, [d])
- The experience of "depersonalization" lasted up to six week after initial onset (Concentration on visual object, [d])
- Depersonalization continued for several weeks (Transcendental meditation, [d])
- Almost constant state of "depersonalization" that is not unpleasant anymore, with confusing episodes of sometimes shifting out of this (Transcendental meditation, [d])

After-effects and impacts

Arousal

Non-specified timelines

Developing a sleeping disorder due to experiments with increasing wakefulness during sleep (Anthroposophic Meditation, [u])

Increased dream activity

Dreams and Lucid Dreams (Buddhist meditation NOS, [e]; Anthroposophic Meditation, [u])

Archetypal dreams relating to emotional processing (Anthroposophic Meditation, [u])
Strange dreams (Buddhist meditation NOS, [e])
Sexual dreams (Buddhist meditation NOS, [e])
Vivid dreams (Buddhist meditation NOS, [e])

Increase in the number of lucid dreams (Anthroposophic Meditation, [u])

Sense of receiving information during dreams (Anthroposophic Meditation, [u])

Sense that one learned meditation techniques in the dream state (Anthroposophic Meditation, [u])
Sense of having either premonitions or experiences relating to past lives when dreaming (Anthroposophic Meditation, [u])
Sense of receiving moral guidance in the dream state (Anthroposophic Meditation, [u])
Sense of encounters with sensed presences (Anthroposophic Meditation, [u])
having intuitions and inspirations related to daily life (Anthroposophic Meditation, [u])

Parasomnias (Buddhist meditation NOS, [p]; Anthroposophic Meditation, [u])

Sense of being strangled by a sensed presence during the night (Anthroposophic Meditation, [u])
Sensing presences in the background while going to sleep (Anthroposophic Meditation, [u])
Sleep Paralysis (Anthroposophic Meditation, [u]; Meditation NOS, [g])

Sense of not being in the body (Anthroposophic Meditation, [u])
hearing a hissing sound (Anthroposophic Meditation, [u])
sensing an evil presence (Anthroposophic Meditation, [u])
strong fear (Anthroposophic Meditation, [u])
unpleasant (Anthroposophic Meditation, [u])

Sense that consciousness generally became more wakeful while falling asleep or during sleep (Anthroposophic Meditation, [u])

Sleep changes (Buddhist meditation NOS, [p])
- **Difficulty sleeping** (Buddhist meditation NOS, [e])
- **Decreased need for sleep** (Anthroposophic Meditation, [u])
- **Increased ability to fall asleep** (Anthroposophic Meditation, [u])
- **Insomnia** (Buddhist meditation NOS, [e])

Afterglow (In the seconds to minutes following)

Tiredness (Jhāna meditation, [t])

Immediate (± 1 day post)

Decreased fatigue (MBSR/Buddhist meditation NOS, [c])
Decreased sleep propensity (MBSR/Buddhist meditation NOS, [c])
Increased energy, alertness and arousal (MBSR/Buddhist meditation NOS, [c])
Insomnia (Meditation NOS, [g]), **Difficulties falling asleep** (Jhāna meditation, [t])

Short-term (< month)

Increased energy, alertness and arousal (MBSR/Buddhist meditation NOS, [c])
Decreased fatigue (MBSR/Buddhist meditation NOS, [c])

Mid-term (>month, <year)

Increased energy, alertness and arousal (MBSR/Buddhist meditation NOS, [c])
Decreased fatigue (MBSR/Buddhist meditation NOS, [c])
Difficulty sleeping (LSD, [10])
Ongoing conscious awareness during sleep (MBSR/Buddhist meditation NOS, [c])
Increased wakefulness and decreased sleep propensity (MBCT, [c])
Reduced total sleep time (Tibetan and Theravada Buddhist meditation, [c])

Behavioral

Non-specified timelines

Avoiding meditation for years after scary meditative experience (Anthroposophic Meditation, [u])

Increased altruistic actions

Using changed attitudes or beliefs in some way to benefit others (Spontaneous NOS, [δ])

Short-term (< month)

Changes in behaviors related to addiction patterns (Ibogaine, [6])

Negative behavior towards self (Meditation NOS, [g])

- **Alcohol Overdose** (Meditation NOS, [g])
- **Self-harm** (Meditation NOS, [g]), Attempts to harm oneself (Classical Psychedelics in lifetime users, [19])
- **Suicide Attempt** (Meditation NOS, [g])
- **Tongue biting** (Meditation NOS, [g])

Negative behavior towards others (Meditation NOS, [g])

- **Aggressive behavior/Aggression** (Meditation NOS, [g]), Attempts to harm others (Classical Psychedelics in lifetime users, [19])
- **Antisocial behavior** (Meditation NOS, [g])
- **Behavior which required physical restraint** (Meditation (various), [l])
- **Interpersonal violence** (Meditation NOS, [g])
- **Social withdrawal** (Meditation NOS, [g])

Cognitive

Non-specified timelines

Calmer mind (Theravadā śamatha and vipassanā Meditation, [h])

Increased ability of meta-cognition (Anthroposophic Meditation, [u])

Increased clarity of mind (Theravadā śamatha and vipassanā Meditation, [h])

Increase in mental sharpness and in levels of penetration (Theravadā śamatha and vipassanā Meditation, [h])

Increased or decreased ability to remember (Anthroposophic Meditation, [u])

Increased mindfulness in daily life (Anthroposophic Meditation, [u])

More peaceful mind (Theravadā śamatha and vipassanā Meditation, [h])

Afterglow (In the seconds to minutes following)

Attentional

Less drift (Jhāna meditation, [t])
Lingering presence (Jhāna meditation, [t])

Greater mindfulness (Buddhist śamatha/vipassanā meditation, [n])

Increased “openness” of mind (Buddhist śamatha/vipassanā meditation, [n])

Increased cognitive flexibility (Buddhist śamatha/vipassanā meditation, [n])

"Mark on the mind" (Jhāna meditation, [t])

Sense of Increased clarity (Buddhist śamatha/vipassanā meditation, [n])

Immediate (± 1 day post)

Attentional

Increased attention (MBSR/Buddhist meditation NOS, [c])

Short-term (< month)

Attentional

Increased attention (MBSR/Buddhist meditation NOS, [c])

Memory

Difficulty remembering what happened: “The experience is like dreaming, and then you have difficulties to remember it” (Salvia divinorum, inhaled, [15])

Mid-term (>month, <year)

Attentional

Increased attention (MBSR/Buddhist meditation NOS, [c])

Poor concentration (LSD, [10])

Memory

Memory problems (LSD, [10])

Emotional

Non-specified timelines

Ability to rapidly evoke desired emotions or moods (Anthroposophic Meditation, [u])
Feeling generally more calm (Anthroposophic Meditation, [u])
Feeling generally much more ready to face life challenges including social confrontations (Anthroposophic Meditation, [u])
Higher levels of positive mood (Meditation NOS, [f])
Increase in one's capacity for self-regulation (Anthroposophic Meditation, [u])
Fear of meditation (Anthroposophic Meditation, [u])
Lower trait anxiety (Meditation NOS, [f])

Afterglow (In the seconds to minutes following)

Contentment (Jhāna meditation, [t])
Feeling more loving (MDMA, ingested, [22])
Less concern for the past or future (Buddhist śamatha/vipassanā meditation, [n])
Openness (Jhāna meditation, [t]; Buddhist śamatha/vipassanā meditation, [n])
Peace (Jhāna meditation, [t])
Satisfaction (Jhāna meditation, [t])

Short-term (< month)

Anxiety (Psilocybin, [7])
Elevation in negative emotion and deterioration in positive emotion (in the week following initial mindfulness training among meditation-native adults) (NOS, [a])
Fear (Psilocybin, [7])
Feeling **Disconnected** from community (Post-truffle retreat, [13])
Sense of having a **Fresh-space to operate from** (MDMA, ingested, [8])
Mood fluctuations (Post-truffle retreat, [13])
'Post-ecstatic blues' (Post-truffle retreat, [13])

Mid-term (>month, <year)

Absence of emotions

No emotions (Transcendental meditation, [d])

Emotional detachment/noninvolvement

Noninvolvement (Transcendental meditation, [d])
Sense of no relation between emotions and "inner being" (Emotional detachment) (Transcendental meditation, [d])
Sense of "evenness of everything" (Transcendental meditation, [d])

Long-term (> year)

Absence of negative emotions (Transcendental meditation, [d])

Baseline affect very contented but not aroused — "not ecstatically joyful, but just a sort of low level of happiness and contentment" (Transcendental meditation, [d])
Even emotional life (Transcendental meditation, [d])
Feeling normal (neither elated nor anything) (Concentration on visual object, [d])
General lack of strong emotions (Transcendental meditation, [d])
Mild pervasive feelings of happiness/contentment/slight positive emotions (Transcendental meditation, [d])
Reduced reactivity to daily life — e.g. being much less affected by monthly period (Concentration on visual object, [d])
Warm overall emotional life (Transcendental meditation, [d])

Existential

Non-specified timelines

Disidentification from mind and body (Theravadā śamatha and vipassanā Meditation, [h])
Diminished or loss of sense of ownership and phenomena, mind, and body (Theravadā śamatha and vipassanā Meditation, [h])
Feeling the relatedness of everything (Theravadā śamatha and vipassanā Meditation, [h])
Lack of connection to everyday reality (Anthroposophic Meditation, [u])
Sense of seeing one's interconnectedness with all phenomena (Theravadā śamatha and vipassanā Meditation, [h])
Sense that mind has gone "beyond ego" (Theravadā śamatha and vipassanā Meditation, [h])
Sense that one has become one with the world (Theravadā śamatha and vipassanā Meditation, [h])
Sense that one cannot label oneself anymore (Theravadā śamatha and vipassanā Meditation, [h])
Sense that there is no doer (Theravadā śamatha and vipassanā Meditation, [h])
Transforming perception based on a subject/object distinction into a holistic one (Theravadā śamatha and vipassanā Meditation, [h])

Afterglow (In the seconds to minutes following)

Less self-centeredness (Buddhist śamatha/vipassanā meditation, [n])

Mid-term (>month, <year)

Sense of being a witness to one's life (Transcendental meditation, [d])
- "like watching a movie where you knew it was a movie" (Transcendental meditation, [d])
- Sense of being a pure impersonal observer watching a movie (Transcendental meditation, [d])

Sense of living a very different kind of living than normal (Transcendental meditation, [d])

Sense of purity (Transcendental meditation, [d])
- "Real pureness" (Transcendental meditation, [d])

Sense of split self — E.g. sense of a real distance between the inner being and the outer being (Transcendental meditation, [d])

Long-term (> year)

Feeling half in the world half out (Concentration on visual object, [d])

Sense of being in a mild state of depersonalization almost constantly (Transcendental meditation, [d])
- "It's like a movie, but a good movie" (Transcendental meditation, [d])

Permanent (± for (most of) the rest of one's life)

Accidental death or suicide (Classical Psychedelics in lifetime users, [19]; Meditation NOS, [g])

Functional

Non-specified timelines

Challenges relating to how one integrates practice in daily life (Anthroposophic Meditation, [u])

Decision Making

Poor insight and judgment (Meditation NOS, [g])
Impulsiveness (Meditation NOS, [g])

Feeling that life is "Insanely more adventurous" (Anthroposophic Meditation, [u])

Increased life competency (Anthroposophic Meditation, [u]; Transcendental meditation, [d])

- **Increased daily function** (Transcendental meditation, [d])
  - "A normal day for me is when everything just goes right" (Transcendental meditation, [d])
- **Increased work efficiency** (Anthroposophic Meditation, [u])
- **Increased awareness for situations in which one can act or intervene beneficially in daily life situations** (Anthroposophic Meditation, [u])
- **Increased capability to making decisions** (Anthroposophic Meditation, [u])
- **Taking action so as to live more in accordance with one's insights and values independently of the opinion of others** (Anthroposophic Meditation, [u])
- **Especially in crisis situations, meditation could be perceived as support** (Anthroposophic Meditation, [u])
- **Sense that meditation can give the necessary detachment to engage with people in a way that involves emotional difficulty** (Anthroposophic Meditation, [u])
- **Challenges related to increase of life competency** (Anthroposophic Meditation, [u])
  - **Feeling strained** (Anthroposophic Meditation, [u])
  - **Sense that one is being attacked during daily life** (Anthroposophic Meditation, [u])
  - **Sense that meditation can lead to a form of detachment that leads to becoming ineffectual** (Anthroposophic Meditation, [u])
  - **Sense that life is harder** (Anthroposophic Meditation, [u])
    - Related with a practice in which one cultivates feeling pain when one encounters untruth (Anthroposophic Meditation, [u])

Occupational impairment (Buddhist meditation NOS, [p]; Spontaneous NOS, [δ]) — *e.g.* Having to give up working for a few days: "I felt the strength of ten men which wore me down so that over the next few days I had to give up working" (Spontaneous NOS, [δ])

Periods of inactivity and reduced functionality in daily life (Anthroposophic Meditation, [u])

Afterglow (In the seconds to minutes following)

Slowness (Jhāna meditation, [t])

Short-term (< month)

Functional Impairment (Classical Psychedelics in lifetime users, [19])

Mid-term (>month, <year)

Functional Impairment (Classical Psychedelics in lifetime users, [19])

Long-term (> year)

[Frequent mild episodes interpreted as of depersonalization/derealization] with **no social or occupational dysfunction** (Transcendental meditation, [d])

Hedonic

Non-specified timelines

Anhedonia (Buddhist meditation NOS, [p])

Mid-term (>month, <year)

Discomfort (because of experience of depersonalization) (Transcendental meditation, [d])

Magical

Non-specified timelines

Apparent "Clairvoyance" or "Precognition" in dreams (NDE, [γ])

Meaningful Connections (synchronicity/synchronicities)

Meaningful connections occurring between one's spiritual practice and daily life events (Anthroposophic Meditation, [u])

Overwhelming amount of meaningful connections, creating a feeling of “complete chaos”, especially if the events are negative (Anthroposophic Meditation, [u])

Medical

Non-specified timelines

Multiple hospitalizations following intense energetic-experiences (Buddhist meditation NOS, [e])

Psychiatric admission following experience (Spontaneous NOS, [δ]) — *e.g.* involving psychiatric injection (Spontaneous NOS, [δ]) or receiving medication. Reports about medical interactions include:

- Interactions with mental health professionals involve little or no meaningful communication (Spontaneous NOS, [δ])
- Sense that the psychiatric treatment was the worst aspects of one's experience (Spontaneous NOS, [δ])
- Sense of being "silenced" by medication — *e.g.*, "In hospital, Jim's attempts to communicate that his 'possession' had ended were silenced with medication" (Spontaneous NOS, [δ])

Short-term (< month)

Seeking subsequent medical attention (Ayahuasca, [3]; Esketamine, [5]; Psilocybin, [7])

Mid-term (>month, <year)

"Hallucinogen-persisting Perception Disorder" diagnosis (LSD, [10])

Seeking subsequent medical attention (Ayahuasca, [3]; LSD, [10]; Classical Psychedelics in lifetime users, [19])

Long-term (> year)

Ketamine bladder syndrome (clinical syndrome associated with chronic usage includes) (Ketamine, [20])

- significantly reduced functional bladder capacity
- very painful bladder
- frequent urination
- Incontinence
- Haematuria
- upper tract obstruction
- papillary necrosis
- ulceration
- active bleeding from most of the urothelium
- Infrequent users may exhibit mild, 'cystitis'-type symptoms

Meta-emergent

Non-specified timelines

Experiences appeared to be significant or meaningful but the meaning were unclear at the time (Spontaneous NOS, [δ])

Feeling a sense of wonder in relation to the notion that meditation practice can have such an effect (Anthroposophic Meditation, [u])

Increased clarity of perception seen as very valuable (Theravadā śamatha and vipassanā Meditation, [h])

Sense that experience was a spontaneous solution to a seemingly unresolvable predicament (Spontaneous NOS, [δ])

Sense that seemingly negative emergent experience was an appropriate reaction to life circumstances — *e.g.*, "In the context of my life I can honestly say “I’m glad I went mad”. You know it was the most sensible thing I could have done, given those circumstances" (Spontaneous NOS, [δ])

Short-term (< month)

Feeling that the **experience was deeply personally beneficial** (Ibogaine, [6])

Feeling that the **experience could be deeply beneficial to others** (Ibogaine, [6])

Motivational

Short-term (< month)

Changed motivations

Feeling a desire to develop more connections with others (Ibogaine, [6])
Feeling a desire to engage in meditative or yogic practice (Ibogaine, [6])
Feeling a desire to study spiritual teachings (Ibogaine, [6])

w

Paradigmatic

Non-specified timelines

Beliefs (general)

"Delusional", "irrational", or "paranormal" beliefs[70] (Buddhist meditation NOS, [p])

Conviction

Conviction that reality is constructed (Theravadā śamatha and vipassanā Meditation, [h])

Gaining a stronger conviction in the fruitfulness of one's spiritual endeavors (Anthroposophic Meditation, [u])

"Can be a consequence of insights" (Anthroposophic Meditation, [u])

Increased lack of conviction or doubt (Anthroposophic Meditation, [u])

"can arise if the practice leads to no specific results or insights" (Anthroposophic Meditation, [u])

Insights

Comprehension of the causal interdependence between worldly perceptions and mental conditions such as mood, personality, interpretation or outlook (Theravadā śamatha and vipassanā Meditation, [h])

Insight that "from the ultimate point of view all things are the same" (Theravadā śamatha and vipassanā Meditation, [h])

Worldviews

Change or transformations in worldviews (Buddhist meditation NOS, [p]; Anthroposophic Meditation, [u])

Belief in a "spiritual world" — E.g. Becoming "as sure that there is a spiritual world as that there is a table that they can touch in front of oneself" (Anthroposophic Meditation, [u])

Non-essentialist worldviews, *e.g.* conviction that "it is all in a process and this process has not any essence of its own" (Theravadā śamatha and vipassanā Meditation, [h])

Seeing everything as impermanent, non-self (Theravadā śamatha and vipassanā Meditation, [h])

Sense that all phenomena are "empty" of intrinsic existence (Theravadā śamatha and vipassanā Meditation, [h])

Short-term (< month)

Changes in worldviews NOS (Ibogaine, [6])

---

70 As we said in the introduction, we do not necessarily endorse terms such as "delusional", "irrational" or "paranormal", which carry ontological and (implicit) value judgments.

Ontological shock (Post-truffle retreat, [13])

Mid-term (>month, <year)

- Changed sense of reality
  - Sense that all emotions or actions are "phoney" from the witnessing perspective ("the inner thing") (Transcendental meditation, [d])
- **Shifts in metaphysical beliefs** away from hard physicalism or materialism and towards panpsychism, dualistic, and fatalistic beliefs (Correlated with improvements in well-being) (Psychedelics NOS, [21])

Long-term (> year)

- Being really passionate about a substance (MDMA, ingested, [8])
- Using psychedelic substances with new purpose in one's life (MDMA, ingested, [8])
- Sense that MDMA is a medicine that need not be taken often (MDMA, ingested, [8])

Perceptual

Non-specified timelines

Ability to “empty consciousness completely” (Anthroposophic Meditation, [u])

Becoming more able to sense certain fine/subtle sensations or atmospheres and even presences (Anthroposophic Meditation, [u])

Integration challenges with being able to **perceive more** or having a **highly sensitized attentional ability as well as having a perceived ability of spiritual observation** (Anthroposophic Meditation, [u])

New NOS perceptual abilities (Anthroposophic Meditation, [u])

Significantly increased "transparency" of phenomena (Theravadā śamatha and vipassanā Meditation, [h])

Significantly increased "vividness" of phenomena (Theravadā śamatha and vipassanā Meditation, [h])

Significantly increased "clarity" of phenomena and perception (Theravadā śamatha and vipassanā Meditation, [h])

Afterglow (In the seconds to minutes following)

"Derealization" (Jhāna meditation, [t])

Mid-term (>month, <year)

Phenomena taking on a slightly warmer and glossier appearance — e.g. "like dew" (Transcendental meditation, [d])

Tactile "hallucinations" (LSD, [10])

Visual "hallucinations" (LSD, [10])

Physiological

Non-specified timelines

General

Addiction (Ketamine, [12])

Fatigue

I felt the strength of ten men - resulted in 'over next few days it wore me down and I had to give up working' (Spontaneous NOS, [δ])

Weight changes (Buddhist meditation NOS, [p])

Vitality and Health

Feeling enlivened, refreshed, and feeling more vital as a result of meditation practice (Anthroposophic Meditation, [u])

Feeling that meditation makes one healthier in general (Anthroposophic Meditation, [u])

Skin

Rash (Meditation NOS, [g])

Hives (Meditation NOS, [g])

Afterglow (In the seconds to minutes following)

Increased energy and vibrancy (Buddhist śamatha/vipassanā meditation, [n])

Immediate (± 1 day post)

Cardiorespiratory

Reduction in heart rate (NOS, [w])

Respiratory suspension (NOS, [w])

Significant increase in 'heart rate deceleration' with respiratory suspension episodes (NOS, [w])

Slowed breathing (NOS, [w])

General

Depleted energy-levels (Ibogaine, [6])

Elimination or attenuation of physiological withdrawal symptoms (Ibogaine, [6])

Pain (Ibogaine, [6]; Buddhist meditation NOS, [i]) — Physical pain which can persist after meditation sessions end (Buddhist meditation NOS, [i]), Bones hurt (Ibogaine, [6])

Muscular

Reduced muscle activity (NOS, [w])

Sensory-motor

Decreased eye activity (NOS, [w])

Mid-term (>month, <year)

Circulatory System

Hemotympanum (Meditation NOS, [g])

Ischaemic attack (Meditation NOS, [g])

Stroke (Meditation NOS, [g])

Gastrointestinal System

Liver problems (Meditation NOS, [g])

Ulcers (Meditation NOS, [g])

Psychological

Non-specified timelines

Attitudes

Changed global attitudes, towards…

- God or religion (NDE, [γ])
- Self (NDE, [γ])
- One's own death (NDE, [γ])
- Death in general (NDE, [γ])
- Life and its meaning (NDE, [γ])

Capacities

Ability to willfully induce an "out of body experience" (Anthroposophic Meditation, [u])

Ability to maintain a series of thoughts on the same subject (Anthroposophic Meditation, [u])

Diminution or absence of attachment (Theravadā śamatha and vipassanā Meditation, [h]), **Significant decrease in attachment/aversion reactions** (Buddhist meditation NOS, [i])

Increased Empathy (Anthroposophic Meditation, [u]; Buddhist meditation NOS, [q])

Increased emotional/psychological sensitivity (to the point of it being painful) (Buddhist meditation NOS, [q])

Much wider perspective (Theravadā śamatha and vipassanā Meditation, [h])

Sense of increased objectivity (Theravadā śamatha and vipassanā Meditation, [h])

Sense that mind has become mature (Theravadā śamatha and vipassanā Meditation, [h])

Difficulties

Depression (Meditation NOS, [g]; Mindfulness, [a]; Buddhist meditation NOS, [i])

Escalation of positive affect into "mania" (Buddhist meditation NOS, [e])

Feeling psychotic (Buddhist meditation NOS, [e]; [i])

Phobia of seeing oneself in the mirror (linked to sense of presence) (Anthroposophic Meditation, [u])

Suicidal crisis (Meditation NOS, [g]), **Suicidality** (Buddhist meditation NOS, [p]; [i])

Integration

Positive experience of integration when insights were accepted (Anthroposophic Meditation, [u])

Insights

The experience provided insight/self knowledge, or a sense of purpose and direction (Spontaneous NOS, [δ])

Personal narratives/Psychological Ego/Character-related changes

Arrogance (Anthroposophic Meditation, [u])
Autonomy (Anthroposophic Meditation, [u])
Egotism (Anthroposophic Meditation, [u])
Increased empathy (Spontaneous NOS, [δ])
Reappraisal of seemingly negative past experiences (Spontaneous NOS, [δ])
Sense that experience had positive impacts on character (Spontaneous NOS, [δ])
Reduced depression (MBSR/Buddhist meditation NOS, [c])
Scrupulosity (Buddhist meditation NOS, [p])
Self-accusation (Anthroposophic Meditation, [u])
Self-determination (Anthroposophic Meditation, [u])
"Self-encounter" (Anthroposophic Meditation, [u])
Selflessness (Anthroposophic Meditation, [u])
Self-strengthening (Anthroposophic Meditation, [u])
- **Increased sense of security**
  - **Increased sense of having a second home** (Anthroposophic Meditation, [u])
- **Inflation of the ego** (Anthroposophic Meditation, [u])**Sense that the self was strengthened, which typically means that**
  - **the sense of personal identity over time increased** (Anthroposophic Meditation, [u])
- **Sense of personal identity came into alignment with one's true life intention** (Anthroposophic Meditation, [u])

Sense that meditation became a source of moral action and an ethical deliberation (Anthroposophic Meditation, [u])
Sense that meditation enabled a connection with the forces of good and the will to serve, to be there for something other than oneself (Anthroposophic Meditation, [u])
Uncertainty (Anthroposophic Meditation, [u])

Afterglow (In the seconds to minutes following)

Less grasping of experience (Buddhist śamatha/vipassanā meditation, [n])

Short-term (< month)

Capacities

Ability to evoke MDMA-like experiences (e.g. sense of newness) in daily life (MDMA, ingested, [8])
Ability to **see one's partner in a new way** (MDMA, ingested, [8])
Feeling unstoppable (Ibogaine, [6])
Reduced or absence of craving (in addicts) (Ibogaine, [6])

Difficulties

Depression (Psilocybin, [7]; Esketamine, [5])
Paranoia (Psilocybin, [7])

Re-experiencing symptoms (Post-truffle retreat, [13])
Spiritual bypass (Post-truffle retreat, [13])
Thoughts of hurting oneself (Classical Psychedelics in lifetime users, [19])

Personal narratives/Psychological Ego/Character-related changes
Decreased anger (Ibogaine, [6])
Decreased self-hatred (Ibogaine, [6])
Decreased self-critical thought patterns (Ibogaine, [6])
Increased sense of self-acceptance and/or **self-pride** (Ibogaine, [6])

Mid-term (>month, <year)

Addiction (Ketamine, [12])

Enduring psychological symptom (Psilocybin, [7])

Improvements in well-being (Psychedelics NOS, [21])

Psychological harm (Esketamine, [5])

Reduced or absence of craving (in addicts) (Ibogaine, [6]) — *e.g.* "About three months after his ibogaine experience, Danny said the following about craving: The ibogaine experience was difficult, but after that, it's been easy ... I haven't had struggles, I haven't had cravings ... or drug dreams, or really no obsessive thoughts. It's just been extremely easy, and the only thing I can attribute that to is ibogaine, for sure, because I've done this countless other times. On my own or with suboxone, cold turkey, or going to rehab, or outpatient, I tried everything. The only thing that I can attribute that to is ibogaine for sure." (Ibogaine, [6])
Flashbacks (LSD, [10])

Long-term (> year)

Increased baseline well-being
"A normal day for me is when everything just goes right" (Transcendental meditation, [d])

Sensate

Afterglow (In the seconds to minutes following)

Increased sensate clarity (Buddhist śamatha/vipassanā meditation, [n])

Mid-term (>month, <year)

Temperature

Sense of Coolness (Transcendental meditation, [d])

Visual

Improvement of color vision in a subject with CVD (Psilocybin mushrooms, ingested, [1])

Increases in vigilance related to **improvements in visual sensitivity** (which persisted at least 3 months after the retreat ended) (Tibetan-style shamatha training, [c])

Social

Non-specified timelines

Agoraphobia (Anthroposophic Meditation, [u])

Sense of **Alienation, Isolation or Disharmony** (Anthroposophic Meditation, [u]; Meditation NOS, [g]; Spontaneous NOS, [δ])

Social alienation (Meditation NOS, [g])
Feeling "completely cut off from everything" (Anthroposophic Meditation, [u])
Sense of social exclusion (Anthroposophic Meditation, [u])
Challenges related to not gaining acceptance for one's own perceived spiritual insights in particular (Anthroposophic Meditation, [u])
Alienation, isolation (Spontaneous NOS, [δ])

Challenges communicating with others about experiences (Spontaneous NOS, [δ]; Anthroposophic Meditation, [u])

Experience is isolating with little or no meaningful discussion with others (Spontaneous NOS, [δ])
Communication "was generally not welcome within the Anthroposophic movement until recent years" (Anthroposophic Meditation, [u])
"I told my wife and I told my parents but it was never really discussed – they kind of didn't know what to say.' (Spontaneous NOS, [δ])
"The subject had their experiences alone and were mostly unable to disclose them to others" (Spontaneous NOS, [δ])
"The subject is unable to disclose his experiences to anyone. They adversely affect his relationships with others, thereby increasing his isolation" (Spontaneous NOS, [δ])

Change in relationship to meditation community (Buddhist meditation NOS, [p])
Increased sense of **Harmony and resonance with the external world** (Anthroposophic Meditation, [u]; Spontaneous NOS, [δ])

Feeling more connected to physical reality and to other human beings (Anthroposophic Meditation, [u])
Sense of community (Anthroposophic Meditation, [u])
Sense of connection to nature and the cosmos as a whole (Anthroposophic Meditation, [u])

Increased sociality (Buddhist meditation NOS, [p])

Integration difficulties following retreat or intensive practice (Buddhist meditation NOS, [p])

Positive response from others — *e.g.* church 'Oh this is fabulous I'm so glad.' He said "You've had a baptism of the holy spirit".' (Spontaneous NOS, [δ])**Social impairment** (Buddhist meditation NOS, [p])

Sense that meditation practice can affect the relationship to other human beings in very complex ways (Anthroposophic Meditation, [u])

Social Anhedonia (Meditation NOS, [g])

Afterglow (In the seconds to minutes following)

Decreased prosocial behavior among those with relatively independent self-construals (Mindfulness, [s])

Increased prosocial behavior among individuals with both interdependent relational and collective self-construals (Mindfulness, [s])

Immediate (± 1 day post)

Being arrested (LSD, [10])

Short-term (< month)

Increased sense of extraversion (Ibogaine, [6])
Sense of disconnection from community (Post-truffle retreat, [13])

Mid-term (>month, <year)

Feeling **Isolated** (LSD, [10])

Perceived **Lack of support** (Post-truffle retreat, [13]; LSD, [10])

Sense of connection

Sense of loss of "any real connection with other people" (Transcendental meditation, [d])

"People that I had known for years - that meant nothing to me" (Transcendental meditation, [d])

Significant social impairment [in the early phase of a long-term depersonalization experience] (Transcendental meditation, [d])

Long-term (> year)

Improved relationships

Sense that MDMA added much depth to intimate relationship (MDMA, ingested, [8])
Sense that the "energetic tether" in all relationships was made much stronger by MDMA (MDMA, ingested, [8])
Sense that MDMA improved both intimate relationships and their connection with family (MDMA, ingested, [8])

Sense that MDMA improved personal communications (MDMA, ingested, [8])
Sense that MDMA helped "tune up" long-term intimate relationship (MDMA, ingested, [8])

Vocational[71]

Non-specified timelines

Changes in career path (Anthroposophic Meditation, [u]; Spontaneous NOS, [δ])

- Formation of psychic workshop (Spontaneous NOS, [δ])
- Volunteer work (Spontaneous NOS, [δ])
- Trained as a psychotherapist (Spontaneous NOS, [δ])
- Writing a book (Spontaneous NOS, [δ])
- Sense that experience changed one's life trajectory (Spontaneous NOS, [δ])

Questioning daily work setting because of spiritual experience about the nature of one's life purpose (Anthroposophic Meditation, [u])

Sense of experience being extremely impactful, **influential** or **life changing** (N,N-DMT, [11])

Long-term (> year)

Changes to life trajectory

- Sense that MDMA changed the course of one's life (MDMA, ingested, [8])

[71] Effects on our livelihoods, careers, education, and the like.

Volitional

Non-specified timelines

Feeling that actions that were typically voluntary now felt involuntary and beyond one's control (Buddhist meditation NOS, [q])

Increased ability to make decisions in daily life (Anthroposophic Meditation, [u])

Wakeful

Afterglow (In the seconds to minutes following)

Profound "reset" of consciousness following intentionally-induced "cessation" (Nirodha-samāpatti) of consciousness that is characterized by:

Cognitive

- **Greater mindfulness** (Buddhist śamatha/vipassanā meditation, [n])
- **Increased "openness" of mind** (Buddhist śamatha/vipassanā meditation, [n])
- **Increased cognitive flexibility** (Buddhist śamatha/vipassanā meditation, [n])
- **Sense of Increased clarity** (Buddhist śamatha/vipassanā meditation, [n])

Emotional

- **Increased "openness" of [...] emotions** (Buddhist śamatha/vipassanā meditation, [n])
- **Less concern for the past or future** (Buddhist śamatha/vipassanā meditation, [n])

Existential

- **Less self-centeredness** (Buddhist śamatha/vipassanā meditation, [n])

Psychological

- **Less grasping of experience** (Buddhist śamatha/vipassanā meditation, [n])

Physiological

- **Increased energy and vibrancy** (Buddhist śamatha/vipassanā meditation, [n])

Sensate

- **Sense of increased clarity** (Buddhist śamatha/vipassanā meditation, [n])

Immediate (± 1 day post)

Conscious awareness during sleep (MBSR/Buddhist meditation NOS, [c])

Additional References

Bickley, L. S., Szilagyi, P. G., and Hoffman, R. M. (2017). *Bates' guide to physical examination and history taking* (Twelfth edition). Wolters Kluwer.

Bitbol, M. (2019). Consciousness, Being and Life: Phenomenological Approaches to Mindfulness. *Journal of Phenomenological Psychology*, *50*(2), 127–161. https://doi.org/10.1163/15691624-12341360

Castillo, R. J. (1990). Depersonalization and Meditation. *Psychiatry*, 53(2), 158–168. https://doi.org/10.1080/00332747.1990.11024497

Fernandez, A. V. (2019). Phenomenology and Dimensional Approaches to Psychiatric Research and Classification. *Philosophy, Psychiatry, and Psychology*, *26*(1), 65–75. https://doi.org/10.1353/ppp.2019.0004

Horváth, L., Szummer, C., & Szabo, A. (2018). Weak phantasy and visionary phantasy: the phenomenological significance of altered states of consciousness. *Phenomenology and the Cognitive Science*s, 17(1), 117–129. https://doi.org/10.1007/s11097-016-9497-4

Kant, I. (2007). *Critique of pure reason* (M. Weigelt, Ed.; F. M. Müller, Trans.). Penguin Books.

Lindahl, J. R., & Britton, W. B. (2019). "I have this feeling of not really being here": Buddhist meditation and changes in sense of self. *Journal of Consciousness Studies*, 26(7–8), 157–183. https://www.imprint.co.uk/wp-content/uploads/2021/03/Lindahl_Open_Access.pdf

Lindahl, J. R., Britton, W. B., and Cooper, D. J. (2022b). Fear and Terror in Buddhist Meditation: A Cognitive Model for Meditation-Related Changes in Arousal and Affect. *Journal of Cognitive Historiography*, 7(1–2). https://doi.org/10.1558/jch.22807

Olivares, F. A., Vargas, E., Fuentes, C., Martínez-Pernía, D., and Canales-Johnson, A. (2015). Neurophenomenology revisited: second-person methods for the study of human consciousness. *Frontiers in Psychology*, *6*. https://www.frontiersin.org/articles/10.3389/fpsyg.2015.00673

Parnas, J. (2012). The core gestalt of schizophrenia. *World Psychiatry*, 11(2), 67–69.)

Petitmengin, C. (2001). *L'expérience intuitive [The Intuitive Experience]*. L'Harmattan.

Petitmengin, C. (2006). Describing one's subjective experience in the second person: An interview method for the science of consciousness. *Phenomenology and the Cognitive Sciences*, 5(3–4), 229–269. https://doi.org/10.1007/s11097-006-9022-2

Petitmengin, C. (2007). Towards the Source of Thoughts. The Gestural and Transmodal Dimension of Lived Experience. *Journal of Consciousness Studies*, *14*(3), 54–82. https://clairepetitmengin.fr/AArticles%20versions%20finales/JCS%20-%20Source.pdf

Petitmengin, C., van Beek, M., Bitbol, M., Nissou, J.-M., and Roepstorff, A. (2017). What is it like to meditate?: Methods and issues for a micro-phenomenological description of meditative experience. *Journal of Consciousness Studies*, 24(5-6), 170–198.

Petitmengin, C., Remillieux, A., & Valenzuela-Moguillansky, C. (2019). Discovering the structures of lived experience: Towards a micro-phenomenological analysis method. *Phenomenology and the Cognitive Sciences*, 18(4), 691–730. https://doi.org/10.1007/s11097-018-9597-4

Sparby, T. (2022). What Stands in the Way Becomes the Way: Dual and Non-Dual Approaches to Meditation Hindrances in Buddhist Traditions and Contemplative Science. *Religions*, 13(9), 840. https://doi.org/10.3390/rel13090840

Timmermann, C., Watts, R., & Dupuis, D. (2022). Towards psychedelic apprenticeship: Developing a gentle touch for the mediation and validation of psychedelic-induced insights and revelations. *Transcultural Psychiatry*, 59(5), 691–704. https://doi.org/10.1177/13634615221082796

Winkelman, M. J. (2018). An ontology of psychedelic entity experiences in evolutionary psychology and neurophenomenology. *Journal of Psychedelic Studies*, 2(1), 5–23. https://doi.org/10.1556/2054.2018.002

Yaden, D. B., & Newberg, A. (2022). *The Varieties of Spiritual Experience: 21st Century Research and Perspectives*. Oxford University Press.
